# A TBC1D9-Rab29 axis controls homeostatic NF-κB signaling and selective IL-6 production in epithelial cells

**DOI:** 10.3389/fcimb.2025.1688013

**Published:** 2025-11-11

**Authors:** Xin Hu, Takashi Nozawa, Atsuko Minowa-Nozawa, Kazunori Murase, Ichiro Nakagawa

**Affiliations:** Department of Microbiology, Graduate School of Medicine, Kyoto University, Kyoto, Japan

**Keywords:** interleukin-6 (IL-6), TBC1D9, Rab29, cytokine regulation, NF-κB signaling

## Abstract

**Background:**

Interleukin-6 (IL-6) plays a crucial role in inflammation and immune defense; however, its intracellular trafficking and the mechanisms regulating its expression remain poorly understood.

**Methods:**

We investigated epithelial cell responses to poly(dA:dT) stimulation and group A *Streptococcus* infection, using depletion and overexpression assays, NF-κB readouts, proteomics, co-immunoprecipitation, immunofluorescence imaging, and analysis of necrotizing soft tissue infection transcriptomes.

**Results:**

TBC1D9, a Rab GTPase-activating protein, selectively regulates IL-6: its depletion reduced IL-6 mRNA and protein levels without broadly affecting other pro-inflammatory cytokines. TBC1D9 maintained basal p65 phosphorylation but was dispensable for stimulus-induced NF-κB activation, supporting the idea that homeostatic NF-κB signaling is necessary for constitutive IL-6 transcription. Proteomics identified Rab29 as a TBC1D9 partner; co-immunoprecipitation showed preferential interaction with GTP-dependent Rab29, and the two proteins co-localized following stimulation and infection. Rab29 overexpression inhibited NF-κB activation and IL-6 production, while Rab29 deficiency increased both, opposing TBC1D9’s effect. Necrotizing soft tissue infection patients’ datasets showed upregulation of TBC1D9 and IL-6-related pathways, revealing their clinical relevance.

**Conclusion:**

The TBC1D9–Rab29 axis connects GTPase signaling and membrane trafficking to specifically regulate IL-6 in epithelial cells, revealing a non-traditional mechanism for modulating inflammation and a potential target in IL-6-driven diseases.

## 1 Introduction

Interleukin-6 (IL-6) is a crucial cytokine activated during bacterial infections, coordinating the inflammatory response (Hunter and Jones, 2015). This multifunctional cytokine is rapidly produced by various cell types, including myeloid lineage cells (macrophages, dendritic cells), polymorphonuclear leukocytes, and non-hematopoietic cells (epithelial and endothelial cells), in response to microbial invasion and tissue damage (Krueger et al., 1991). Epithelial cells form the initial barrier against microbial invasion and actively influence innate immune responses. IL-6 enhances host defense by promoting neutrophil recruitment, T-cell differentiation, and acute-phase protein production. However, excessive IL-6 expression can provoke hyperinflammation, leading to immune-mediated pathologies (Hirano, 2020). Therefore, strict regulation of IL-6 expression is vital to balance effective immune defense with the prevention of immunopathology. The IL-6 promoter contains multiple *cis*-regulatory elements that integrate signals from various transcription factors like NF-κB, NF-IL6 (C/EBPβ), AP-1, and others (Dendorfer et al., 1994). Among these, NF-κB occupies a central role in mediating the rapid induction of cytokines following innate immune activation (Rahman and McFadden, 2011). Following recognition of pathogen-associated molecular patterns (PAMPs), the IκB kinase (IKK) complex is activated, leading to the degradation of IκB proteins and subsequent nuclear translocation of NF-κB (p65/p50) to initiate cytokine transcription (Liu et al., 2017; Ni et al., 2018). Meanwhile, the cytosolic DNA sensor cGAS detects bacterial or damaged organelle-derived dsDNA, activating the cGAS–STING pathway (Chen et al., 2016; Estrela et al., 2019). Activated STING translocates to the Golgi apparatus, recruiting TANK-binding kinase 1 (TBK1), which activates IKK complexes to further promote NF-κB nuclear translocation and cytokine production (Hu et al., 2021). Recent studies suggest that TBK1 and its paralog IKKϵ may enhance NF-κB signaling in response to specific stimuli, such as cytosolic DNA or pathogen invasion, potentially connecting cytosolic DNA sensing or pathogen invasion to cytokine transcription (Balka et al., 2020; Yu and Liu, 2021). Additionally, cytokine expression is tightly regulated at the transcriptional level by elements in the 5′ and 3′untranslated regions (UTRs) of mRNA. The 5′ UTR influences translation initiation, while the 3′ UTR affects mRNA stability (Tanaka et al., 2014). RNA-binding proteins (RBPs), such as AT-rich interactive domain-containing protein 5A (Arid5a), bind to the 3′ UTRs of IL-6 mRNA to enhance the stability of mRNA transcripts and prevent their degradation (Gingerich et al., 2004; Masuda et al., 2013). In contrast, the endoribonuclease Regnase-1 promotes the decay of IL-6 mRNA, serving as a negative regulator (Mao et al., 2017). This balance ensures IL-6 is produced properly in response to immune stimuli, preventing excessive inflammation. Therefore, we asked whether pathogen-triggered innate sensing, such as cGAS‐STING, activates NF-κB to regulate IL-6 responses, and whether targeted regulation of IL-6, instead of broad cytokine suppression, could reduce the risk of excessive inflammation.

Group A *Streptococcus* (GAS, *Streptococcus pyogenes*) is a versatile human pathogen that causes infections from superficial pharyngitis to severe invasive diseases such as necrotizing fasciitis and toxic shock syndrome. Its pathogenicity involves inducing hyperinflammatory responses, leading to cytokine storms (Lappin and Ferguson, 2009; Wilde et al., 2021). Clinical observations show that patients with invasive GAS infections have significantly elevated levels of key cytokines, including IL-6 and other pro-inflammatory cytokines (Nakagawa et al., 2004; Su et al., 2024). Understanding the regulation of IL-6 in GAS infections is thus critical for developing targeted therapeutic strategies.

Recent studies show Tre-2/Bub2/Cdc16 (TBC) domain-containing protein family modulate immune signaling beyond their classical role as GTPase-activating proteins (GAPs) for Rab GTPases. In particular, TBC1D15 inhibits cytosolic DNA sensing by inhibiting the STING pathway (Kwon et al., 2017). while TBC1D23 has been shown to regulate cytokine production in macrophages upon stimulation through Toll-like receptors (TLRs) and lipopolysaccharide (LPS) exposure (De Arras et al., 2012). Although Rab GTPases are involved in immune responses, their specific functions in cytokine regulation remain unclear. Our previous work showed that TBC1D9 is involved in calcium-dependent activation of TBK1 and promotes xenophagy during GAS infection (Nozawa et al., 2020). However, the mechanisms by which TBC1D9 influences innate immune signaling pathways, particularly IL-6 regulation, require further exploration.

In this study, we used poly(dA:dT), a synthetic cytosolic DNA analog that mimics PAMP recognition (Liu et al., 2024), and the non-invasive GAS strain JRS4 (serotype M6; hereafter referred to as GAS) to investigate TBC1D9’s role in innate immunity, specifically regarding IL-6 expression. HeLa cells served as the primary model, with key findings confirmed in A549 epithelial cells. We found that TBC1D9 specifically regulates IL-6 through homeostatic NF-κB, while Rab29 suppresses this pathway by inhibiting NF-κB and IL-6 in a GTP-dependent manner. Despite their opposing regulatory functions, TBC1D9 physically interacts with Rab29, and this interaction is essential for IL-6 production, which reveals a functionally coordinated mechanism within the Rab GTPase signaling network. We also integrate transcriptomic profiling from necrotizing soft tissue infection (NSTI) samples to position TBC1D9 within IL-6-related inflammatory pathways, providing clinical relevance to our mechanistic findings. Together, our data identify a TBC1D9-Rab29 axis that couples signaling with spatial regulation to modulate IL-6: it maintains the homeostatic NF-κB and organizes signaling complexes in specific subcellular compartments.

## 2 Methods

### 2.1 Cell culture and treatments

HeLa, HEK293T, and A549 cells (American Type Culture Collection) were cultured in Dulbecco’s Modified Eagle Medium (DMEM; Nacalai Tesque) with 10% fetal bovine serum (FBS; Thermo Fisher Scientific) and 50 μg/mL gentamicin (Nacalai Tesque) at 37°C in a humidified 5% CO_2_ incubator. Cells were passaged at 80-90% confluence. HeLa cells served as the primary experimental model for infection-induced IL-6 regulation, and key findings were confirmed in A549 cells; HEK293T cells were used only for protein-protein interaction assays, including mass spectrometry.

### 2.2 Bacterial culture

The bacterial strains used in this study are listed in **Supplementary Table S1**.

GAS strains JRS4 (serotype M6) and SSI-1 (serotype M3) were cultured in Todd-Hewitt broth supplemented with 0.2% yeast extract (THY) at 37°C under aerobic conditions. *S. aureus* was grown in tryptic soy broth containing 0.2% yeast extract (TSY), and *S. enterica* in Luria-Bertani (LB) broth, also at 37°C under aerobic conditions. Overnight cultures were reinoculated into fresh medium, grown to the exponential phase (optical density at 600 nm [OD600] of 0.7-0.8), harvested by centrifugation, and diluted in cell culture medium before infection.

For infection, host cells were incubated with bacteria at a multiplicity of infection (MOI) of 100 in antibiotic-free medium for 1 hour. After infection, cells were washed once with PBS and treated with gentamicin (100 µg/mL) for 3 hours to eliminate extracellular bacteria. At 4 hours post-infection, cell lysates and culture supernatants were collected for downstream analyses, including qRT-PCR, ELISA, and others.

### 2.3 Generation of KO cells

*TBC1D9*-KO and *TBK1*-KO HeLa cells were previously established in our lab (Nozawa et al., 2020). *TBC1D9*-KO A549 and *Rab29*-KO HeLa cells were generated using the CRISPR-Cas9 gene editing system. sgRNA sequences targeting human TBC1D9 (sgRNA: 5’-AACCCGGAGGAGGTGTTGC-3’) and Rab29 (sgRNA: 5’-GCACACTACCCAATGGAGAGC -3’) were cloned into the pSpCas9(BB)-2A-Puro (px459) V2.0 vector. HeLa and A549 cells were transfected with the respective plasmids and selected in medium containing 2 μg/mL puromycin for 48 hours. Single-cell clones were isolated and expanded in 24-well plates. Knockout efficiency was confirmed by immunoblotting.

### 2.4 Plasmid construction, gene KD and cell transfection

Human TBC1D9 (Nozawa et al., 2020), Rab1a (Toh et al., 2020), Rab5a, Rab5b, Rab5c, Rab7a, Rab8a, Rab10, Rab11a, Rab14, Rab21, and Rab39b (Minowa-Nozawa et al., 2025) plasmids were previously generated in our lab. Rab2b, Rab6a, Rab6b, Rab12, Rab28, Rab29, Rab42, and Arid5a cDNAs were amplified by PCR from HEK293T cDNA, and cloned into a pENTR/D‐TOPO vector using a pENTR Directional TOPO cloning kit (Invitrogen, Cat# K2400-20) or pENTR1A vector (Invitrogen, Cat# A10462) using the *Escherichia coli* iVEC3. The cDNAs in pENTR1A were transferred to: pcDNA-6.2/N-EmGFP-DEST, pcDNA-6.2/N-3xFLAG-DEST, pcDNA-6.2/N-mCherry-DEST, and pGEX-6P-1-DEST (Invitrogen) using Gateway-compatible vectors (LR clonase). All constructs encode N-terminal tagged proteins. The TBC1D9 mutant (R566K) (Nozawa et al., 2020) was generated previously, while Rab29 mutants (Q67L and T21N) were made using site-directed mutagenesis with the PrimeSTAR Mutagenesis Basal Kit (Takara). Primers used in this study are listed in the **Supplementary Table S2**. Plasmids were transfected with Lipofectamine 3000 (Invitrogen).

### 2.5 Poly(dA:dT) stimulation

Cells were transfected with 1 μg/mL poly(dA:dT) (InvivoGen) using Lipofectamine 3000 (Invitrogen) for 6 hours. Cell lysates and culture supernatants were collected for downstream analyses, including qRT-PCR, ELISA, and others.

### 2.6 Quantitative reverse transcription-PCR

Total RNA was extracted and reverse-transcribed using PrimeScript II (Takara) according to the manufacturer’s instructions. Quantitative PCR (q-PCR) used SsoAdvanced SYBR Green Supermix (Bio-Rad) on a CFX Connect system (Bio-Rad). Primers were designed with Primer3 and listed in **Supplementary Table S2**. PCR conditions: initial denaturation at 95°C for 40 seconds, then 40 cycles at 95°C for 15 seconds and 63°C for 45 seconds. Melting curve analysis confirmed specificity. Gene expression was normalized to GAPDH and calculated via 2^–ΔΔCt^ method.

### 2.7 ELISA

IL-6 levels in cell culture supernatants were measured using a human IL-6 ELISA kit (Proteintech) according to the manufacturer’s instructions.

### 2.8 Luciferase reporter assay

HeLa cells in 96-well plates were co-transfected with the pNL3.2-NF-κB reporter plasmid and the internal control plasmid pGL4.53 [luc2/PGK] (Promega), along with Rab proteins or control plasmids. After 24 hours, luciferase activity was measured in cell lysates using the Dual-Luciferase Reporter Assay System (#E1910, Promega), according to the manufacturer’s instructions.

### 2.9 Actinomycin D chase assays

HeLa-wild-type (WT) and *TBC1D9*-KO cells were stimulated with poly(dA:dT) for 6 hours. Transcription was stopped with 10 μg/ml actinomycin D (Nacalai Tesque) without changing the medium. RNA was extracted at 0, 1, 2, 4, 6 hours. IL-6 mRNA levels were normalized to GAPDH. Relative transcript levels at each time point were calculated using the 2^–ΔΔCt^ method, with 0 hours as 100%.

### 2.10 Immunoblotting

Cells were lysed in 1× SDS sample buffer (50 mM Tris-HCl, pH6.8, 2% SDS, 10% Glycerol, 6% 2-Mercaptoethanol), boiled 5 minutes, and proteins resolved by SDS-PAGE then transferred to PVDF membranes. Membranes were blocked with 5% skim milk in TBS-T for 1 hour at room temperature, followed by overnight incubation with primary antibodies at 4°C. After washing, membranes were incubated with HRP secondary antibodies for 1 hour at room temperature. Signals were developed with Chemi-Lumi One Super and imaged with a Fusion Solo S system. Antibodies are listed in **Supplementary Table S3**.

### 2.11 Co-immunoprecipitation

HeLa cells were co-transfected with FLAG-TBC1D9 and GFP-Rab29 and mutants. After 48 hours, stimulated as indicated, cells were lysed on ice in buffer (50 mM Tris-HCl pH 8.0, 120 mM NaCl, 1% NP-40, 50 mM NaF) with protease inhibitors. Lysates were cleared and incubated with GFP-Trap (ChromoTek) beads for 2 hours at 4°C, then washed five times and analyzed by immunoblotting.

### 2.12 Confocal microscopy

Cells co-expressing mCherry-tagged TBC1D9 and GFP-tagged Rab29 were fixed in 4% paraformaldehyde for 15 minutes, washed with PBS, and stained with DAPI to visualize DNA. For immunofluorescence, cells were permeabilized with 0.1% Triton X-100 for 10 minutes, blocked in 2% BSA for 1 hour, and incubated with primary antibodies overnight at 4°C. After PBS washes, secondary antibodies were incubated for 1 hour followed by DAPI staining. Antibodies listed in **Supplementary Table S3**. Images were acquired using an LSM900 confocal microscope (Zeiss) with a 63× oil objective.

### 2.13 NF-κB inhibition assay

To assess the contribution of NF-κB signaling to IL-6 expression, HeLa cells were pretreated with the NF-κB inhibitor BAY 11-7082 (Apollo Scientific) at final concentrations of 2.5 μM and 5 μM for 1 hour. Following inhibitor removal, cells were washed once with PBS and subsequently stimulated with poly(dA:dT) for 6 hours or infected with GAS (MOI = 100) for 4 hours. Culture supernatants or RNA samples were harvested immediately after stimulation. Cells treated with DMSO served as vehicle controls. All conditions were performed in biological triplicate.

### 2.14 GTPase-Glo assay

GST-tagged TBC1D9 and Rab29 proteins were expressed in *Escherichia coli* BL21 (DE3) using pGEX-6P-1 constructs (GE Healthcare). Bacteria were cultured in LB medium supplemented with 100 μg/mL ampicillin at 37°C and induced with 0.3 mM IPTG (Nacalai Tesque) for 3 hours. Cells were harvested, washed with PBS, and lysed in buffer (40 mM Tris-HCl pH 7.5, 5 mM EDTA, 0.5% Triton X-100). Lysates were sonicated and cleared by centrifugation. The supernatants were incubated with Glutathione Sepharose 4 Fast Flow beads (Cytiva) for 2 hours at 4°C. Beads were washed, and purified proteins were validated by immunoblot and prepared for activity assays.

*In vitro* GTP hydrolysis was measured using the GTPase-Glo Assay Kit (Promega) according to the manufacturer’s instructions. Immobilized GST-TBC1D9 and GST-Rab29 proteins were incubated with GTP at 37°C for 2 hours. Luminescence was recorded as an indicator of GTPase activity.

### 2.15 Mass spectrometry

HEK293T cells were transfected with GFP, GFP-TBC1D9, or GFP-TBC1D9 R566K constructs. After 48 hours, cell lysates were precipitated with GFP-Trap beads as described above. Eluted proteins were denatured at 100°C for 5 min and subjected to acetone precipitation.

Purified tryptic peptides were dried and resuspended in 0.1% formic acid. Samples were analyzed by liquid chromatography-tandem mass spectrometry using an Easy-nLC 1000 system (Thermo Scientific) coupled to a Q-Exactive mass spectrometer (Thermo Scientific). Peptides were separated at a flow rate of 300 NL/min using a trap column (Acclaim PepMap, Thermo Scientific) and an analytical capillary column (Nikkyo Technos). The mobile phases consisted of 0.1% formic acid in water (solvent A) and 0.1% formic acid in acetonitrile (solvent B). Peptides were eluted using a gradient from 1% to 35% solvent B. The elution gradient was programmed as follows: 0–20% B for 0–80 min, 2–45% B for 80–110 min, 45–95% B for 110–111 min, and 95% B for 111–120 min. Mass spectrometry was performed on a Q-Exactive spectrometer (Thermo Scientific) in the electrospray ionization-positive mode. Protein identification using MS/MS data was performed using Proteome Discoverer software (ver.2.5.0, Thermo Scientific) with the UniProtKB/Swiss-Prot database.

### 2.16 Transcriptomic analysis of TBC1D9 expression in NSTIs

Publicly available RNA-seq datasets from patients with NSTIs (PRJNA479582) and healthy controls (GSE249027) were retrieved for comparative transcriptomic analysis. Sequencing reads were aligned to the Homo sapiens reference genome (GRCh37.p13) using STAR aligner (version STAR 2.7.10b). Transcript abundance was quantified using featureCounts (v2.0.6). Normalized transcript expression values (TPM) were used for visualization purposes only. Differential gene expression analysis was performed using the DESeq2 package (version1.46.0) in R (version 4.4.2). Genes with *p<*0.05 and |log_2_FC|≥2 were considered differentially expressed genes (DEGs). Hierarchical clustering analysis was performed to assess global gene expression patterns, and volcano plots were generated to visualize the distribution of DEGs. TBC1D9 was identified as a significantly upregulated gene in NSTI samples. Gene set enrichment analysis (GSEA) was performed in Standard GSEA mode using the Broad Institute GSEA software (version 4.4.0) to identify enriched pathways, among which the IL-6 signaling pathway was prominently represented.

### 2.17 Statistical analysis

Western blot and immunoprecipitation were independently repeated at least three times, with representative blots shown. Band intensities were quantified using ImageJ software (NIH). For immunofluorescence quantification, a minimum of 50 cells per condition were analyzed across three independent experiments. p65 was detected and quantified in nuclear and perinuclear cytoplasmic ROIs in Fiji/ImageJ; nuclear-to-cytoplasmic (Nuc/Cyt) intensity ratios were calculated from background-subtracted means. Pearson’s correlation coefficients were calculated using the JACoP plugin in ImageJ with manual thresholding. Nonlinear regression (one-phase exponential decay) was used for curve fitting to evaluate differences in mRNA half-lives.

All data are presented as mean ± SEM from at least three independent experiments. Normality (Shapiro-Wilk) and homoscedasticity (Levene) were assessed. Data were tested by two-tailed Student’s t test. Significance in figures: ns (*p*≥ 0.05), * (*p<*0.05), ** (*p<* 0.01), *** (*p<*0.001), **** (*p<* 1×10^-4^). All statistical analyses were conducted using GraphPad Prism 10.

## 3 Results

### 3.1 TBC1D9 selectively regulates IL−6 production in epithelial cells during cytosolic DNA sensing and bacterial infection

**Figure 1:**
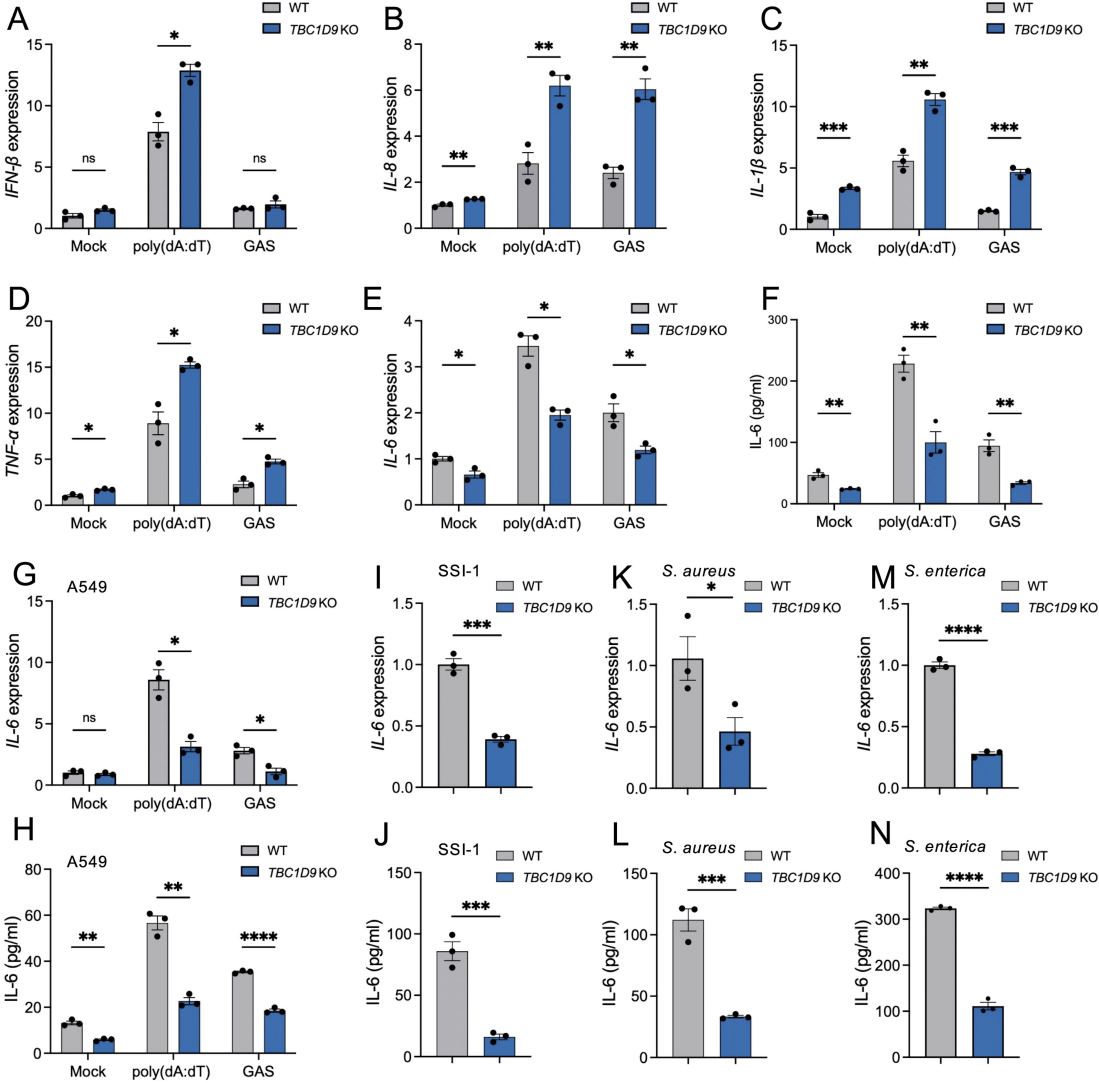
TBC1D9 selectively regulates IL-6 expression in response to cytosolic DNA and bacterial infections. HeLa or A549 cells (WT or *TBC1D9*-KO) were mock-treated, stimulated with 1 μg/mL poly(dA:dT) for 6 hours, or infected with GAS (JRS4), GAS SSI-1, *S. aureus*, or *S. enterica* (MOI 100) for 4 hours. qRT-PCR data were normalized to GAPDH and expressed as fold changes relative to mock-treated WT cells. **(A–D)** mRNA expression in HeLa cells: IFN-β **(A)**, IL-8 **(B)**, IL-1β **(C)**, TNF-α **(D)**. **(E, F)** IL-6 mRNA expression **(E)** and secretion **(F)** in HeLa cells. **(G-H)** IL-6 mRNA expression **(G)** and secretion **(H)** in A549 cells. **(I, J)** IL-6 mRNA expression **(I)** and secretion **(J)** in HeLa cells infected with GAS SSI-1. **(K, L)** IL-6 mRNA expression **(K)** and secretion **(L)** in HeLa cells infected with *S. aureus*. **(M, N)** IL-6 mRNA expression **(M)** and secretion **(N)** in HeLa cells infected with S*. enterica*. Data are shown as mean ± SEM (n=3 independent experiments). Significance: ns (*p* ≥ 0.05), * (*p* < 0.05), ** (*p* < 0.01), *** (*p* < 0.001), **** (*p* < 1×10^-4^), determined by two-tailed Student’s t-test.

To investigate TBC1D9’s role in innate immune responses, we utilized TBC1D9 knockout HeLa cells (*TBC1D9*-KO) and treated them with poly(dA:dT) for 6 hours and GAS infection for 4 hours. Loss of TBC1D9 significantly increased mRNA levels of several pro-inflammatory cytokines, including IFN-β, IL-8, IL-1β, and TNF-α (**Figures 1A–D**). In contrast, IL-6 mRNA expression was significantly suppressed, with reductions of 1.77-fold following poly(dA:dT) stimulation and 1.68-fold after GAS infection (*p<*0.05, **Figure 1E**). Consistent with this transcriptional suppression, IL-6 protein secretion was also decreased in *TBC1D9*-KO cells (**Figure 1F**), suggesting that TBC1D9 regulates IL-6 expression at both transcription and secretion levels. The selective reduction of IL-6, despite increased expression of other cytokines, indicates that TBC1D9 plays a specific role in modulating innate immune signaling.

In our study, HeLa cells served as the primary model for mechanistic studies because they have stable gene editing and detailed signaling analysis. Key observations were independently validated in A549 alveolar epithelial cells. To evaluate whether TBC1D9’s conservation across epithelial cells, we examined IL-6 expression in WT and *TBC1D9*-KO A549 cells (**Supplementary Figure S1A**). Consistent with our HeLa cell observations, poly(dA:dT) stimulation or GAS infection in *TBC1D9*-KO A549 cells significantly reduced IL-6 expression (*p* < 0.05; **Figures 1G, H**), underscoring the broader relevance of TBC1D9 in epithelial immune responses. To determine whether TBC1D9’s regulatory effect on IL-6 extends to other bacterial pathogens, we infected HeLa *TBC1D9*-KO cells with a hypervirulent GAS strain (SSI-1, serotype M3), *Staphylococcus aureus* (*S. aureus*), or *Salmonella enterica* (*S. enterica*). Across these infections, IL-6 mRNA expression and protein secretion were consistently reduced in *TBC1D9*-KO cells compared to WT controls (*p* < 0.05; **Figures 1I–N**), indicating that TBC1D9 regulates IL-6 responses to various bacterial pathogens. To determine if TBC1D9’s role during hypervirulent infection extends beyond IL-6, we infected epithelial cells with GAS SSI-1 (M3) and measured the expression of IFN-β, TNF-α, IL-1β, and IL-8. In both HeLa (**Supplementary Figures S2A-D**) and A549 (**Supplementary Figures S3A-D**) cells, these cytokines were not reduced by TBC1D9 knockout. Therefore, during the SSI-1 infection, TBC1D9 does not significantly increase IFN-β, TNF-α, IL-1β, or IL-8, indicating its specific role in regulating IL-6 within epithelial environments.

Together, these findings identify TBC1D9 as an essential and selective regulator of IL-6 expression in response to both cytosolic DNA sensing and diverse bacterial infection.

### 3.2 TBC1D9 sustains homeostatic NF-κB activity but is dispensable for stimulus-induced NF-κB activation

**Figure 2:**
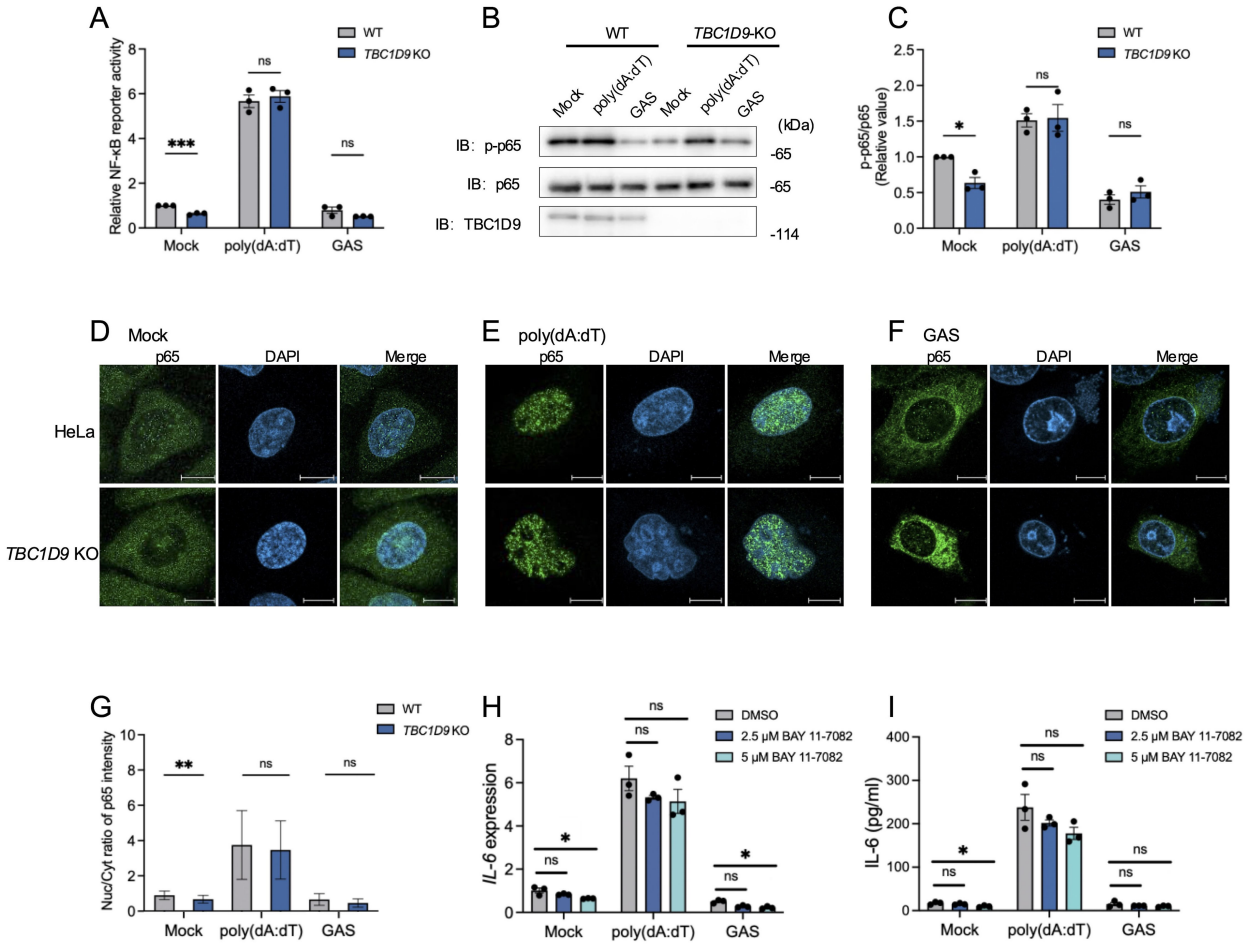
TBC1D9 sustains homeostatic NF-κB activity but is dispensable for stimulus-induced NF-κB activation. HeLa cells (WT or *TBC1D9*-KO) were mock-treated, stimulated with 1 μg/mL poly(dA:dT) for 6 hours, or infected with GAS (JRS4) for 4 hours. **(A)** NF-κB luciferase reporter activity, normalized to mock-treated WT cells. **(B, C)** Western blot of p-p65 and p65 **(B)** and quantification of p-p65/p65 ratio, normalized to mock-treated WT cells **(C)**. **(D–G)** Representative confocal images of NF-κB p65 (green) and DAPI-stained nuclei (blue) in mock-treated **(D)**, poly(dA:dT)-stimulated **(E)**, or GAS-infected **(F)** cells. Scale bar: 10 μm; Nuclear-to-cytoplasmic (Nuc/Cyt) of p65 fluorescence ratios **(G)**. **(H, I)** IL-6 mRNA expression **(H)** and secretion **(I)** in HeLa cells pretreated with 2.5 or 5 μM BAY 11–7082 for 1 hour, then stimulated with poly(dA:dT) or infected with GAS. qRT-PCR data were normalized to GAPDH and presented as fold changes relative to DMSO-treated controls. Data are shown as mean ± SEM (n=3 independent experiments). Nuc/Cyt p65 fluorescence ratio was quantified from >50 cells per condition **(D)**. Significance: ns (*p* ≥ 0.05), * (*p* < 0.05), ** (*p* < 0.01), *** (*p* < 0.001), determined by two-tailed Student’s t-test.

Given the crucial role of NF-κB in IL-6 production, we measured NF-κB activity via luciferase reporter assays in HeLa WT and *TBC1D9*-KO cells. Under basal conditions (Mock), *TBC1D9*-KO cells showed significantly reduced NF-κB activity compared to WT; However, following poly(dA:dT) stimulation, both cell types showed strong NF-κB activation without significant differences; Similarly, GAS infection also suppressed NF-κB activity to comparable levels in both genotypes (**Figure 2A**). To further investigate this process’s molecular regulation, we examined phosphorylation of the NF-κB subunit p65, a central component of the canonical NF-κB pathway. Total p65 levels remained unchanged between WT and *TBC1D9*-KO cells. However, phosphorylated p65 (p-p65) levels were significantly lower in *TBC1D9*-KO cells under basal conditions, indicating impaired constitutive p65 activation. Consistent with the reporter assay, poly(dA:dT) stimulation induced similar p-p65 levels in both genotypes, while GAS infection reduced p-p65 levels similarly in WT and *TBC1D9*-KO cells (**Figures 2B, C**). Moreover, nuclear translocation of p65, assessed by immunofluorescence, was reduced in *TBC1D9*-KO cells under basal conditions but showed no significant differences after poly(dA:dT) stimulation or GAS infection (**Figures 2D-G**), indicating an impairment of homeostatic NF-κB activity. Furthermore, to confirm how homeostatic NF-κB in IL-6 expression, we pretreated WT HeLa cells with the IκBα phosphorylation inhibitor BAY 11-7082 (2.5 μM or 5 μM) for 1 hour, then removed the inhibitor and stimulated with poly(dA:dT) or GAS. The results showed that BAY 11–7082 treatment alone significantly reduced IL-6 mRNA levels and secretion under basal conditions (*p<*0.05, **Figures 2H, I**), confirming that NF-κB activity is necessary to sustain basal IL-6 expression.

These findings show that TBC1D9 is required for sustaining constitutive p65 phosphorylation and homeostatic NF-κB activity, thereby promoting IL-6 expression. In contrast, NF-κB activation in response to exogenous stimuli appears independent of TBC1D9.

### 3.3 TBC1D9–Rab29 axis regulates IL-6 production via GAP activity–dependent modulation of NF-κB signaling

**Figure 3:**
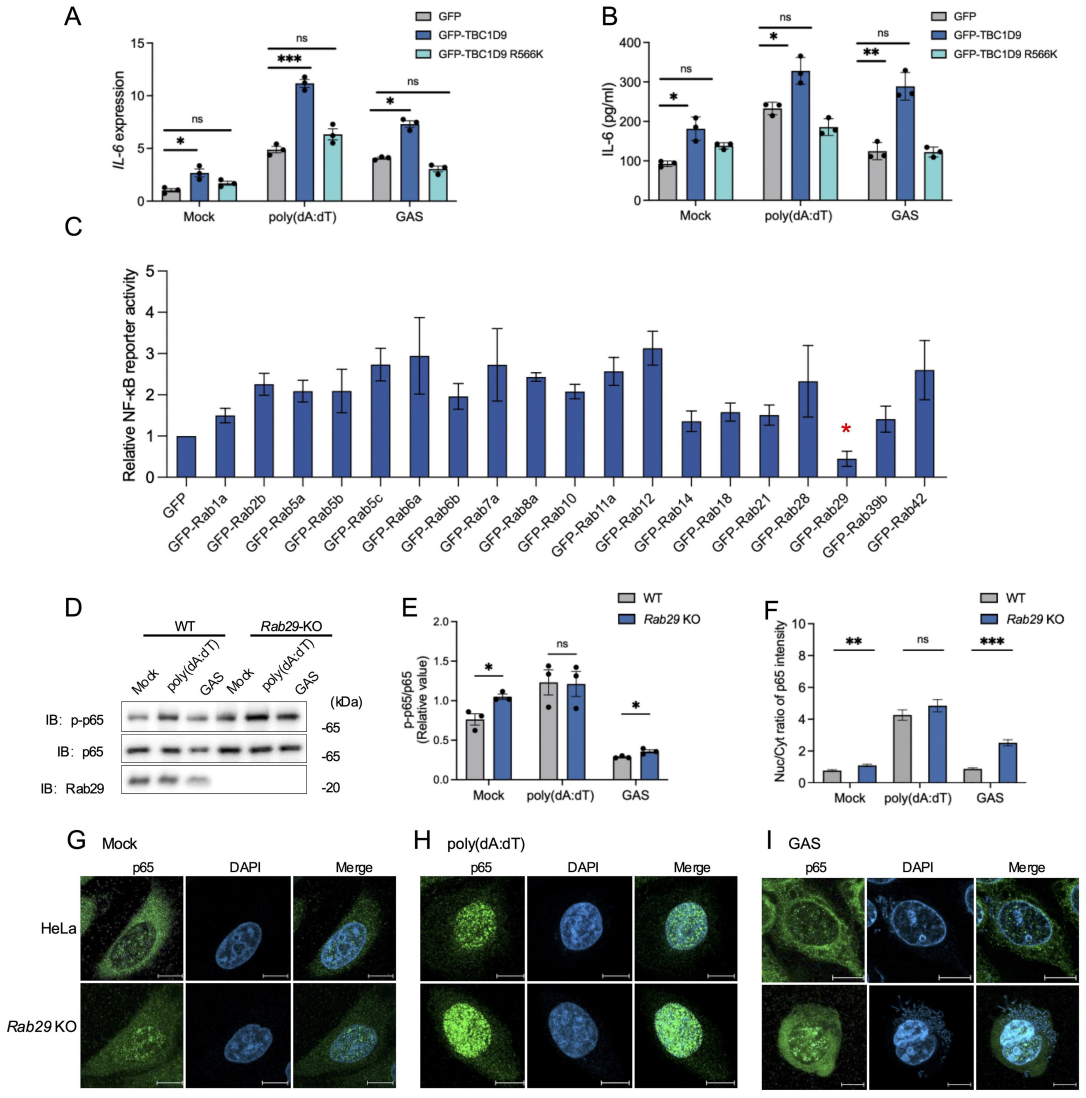
TBC1D9–Rab29 axis regulates IL-6 production via GAP activity–dependent modulation of NF-κB signaling. HeLa cells (WT, *TBC1D9*-KO, or *Rab29*-KO) were mock-treated, stimulated with 1 μg/mL poly(dA:dT) for 6 hours, or infected with GAS (JRS4) for 4 hours. **(A, B)** IL-6 mRNA expression **(A)** and secretion **(B)** in *TBC1D9*-KO cells expressing GFP, GFP-TBC1D9, or GFP-TBC1D9 R566K (GAP-deficient). qRT-PCR data were normalized to GAPDH and expressed as fold changes relative to mock-treated *TBC1D9*-KO cells expressing GFP. **(C)** NF-κB luciferase reporter activity in HeLa cells overexpressing Rab GTPases, normalized to GFP controls. **(D, E)** Western blot of p-p65 and p65 in WT and *Rab29*-KO cells **(D)** and quantification of p-p65/p65 ratio, normalized to mock-treated WT cells **(E)**. **(F–I)** Nuclear-to-cytoplasmic (Nuc/Cyt) of p65 fluorescence ratios **(F)**; representative confocal images of NF-κB p65 (green) and DAPI-stained nuclei (blue) in WT and *Rab29*-KO cells: mock-treated **(G)**, poly(dA:dT)-stimulated **(H)**, or GAS-infected **(I)**. Scale bar: 10 μm. Data are shown as mean ± SEM (n=3 independent experiments). Nuc/Cyt p65 fluorescence ratio was quantified from >50 cells per condition **(F)**. Significance: ns (*p* ≥ 0.05), * (*p* < 0.05), ** (*p* < 0.01), *** (*p* < 0.001), determined by two-tailed Student’s t-test.

To clarify the molecular mechanism by which TBC1D9 regulates IL-6 expression, we examined how its functional domains: the GRAM domain, EF-hands motif, and the TBC domain (amino acids 515–702), which confers GAP activity toward Rab GTPases (Nozawa et al., 2020). To assess the role of GAP activity functions, we generated a GAP-deficient mutant (R566K), and overexpressed a GFP vector control, GFP-tagged TBC1D9 WT, and GFP-tagged TBC1D9 R566K in *TBC1D9*-KO HeLa cells. The results showed that only TBC1D9-WT rescued IL-6 mRNA and protein levels under basal or stimulated conditions (**Figures 3A, B**), indicating that TBC1D9’s GAP activity is essential for regulating IL-6 production.

To investigate how TBC1D9 affects cellular homeostasis and immune signaling, we conducted mass spectrometry-based proteomics on GFP-tagged TBC1D9 WT and TBC1D9 R566K mutant in HEK293T cells under basal conditions, due to these cells’ high transfection efficiency and robust expression of tagged constructs. Proteomics analysis revealed extensive proteome remodeling following TBC1D9 WT overexpression, with fewer changes observed for the TBC1D9 R566K mutant (Data S1), indicating the significance of GAP activity. Considering that Rab GTPase is known to act as a substrate for TBC-domain-containing proteins, we investigated their presence in TBC1D9-associated proteomic profiles. We then evaluated their impact on NF-κB signaling using a luciferase reporter assay. Interestingly, most Rab GTPases increased NF-κB reporter activity, whereas Rab29 uniquely inhibited it (**Figure 3C**, **Supplementary Figures S4A, B**). To further investigate Rab29’s regulatory role, we then generated Rab29-knockout HeLa cells (*Rab29*-KO) using the CRISPR-Cas9 system (**Supplementary Figure S1B**). *Rab29*-KO cells exhibited increased p-p65 levels under basal conditions and after GAS infection, but not following poly(dA:dT) stimulation (**Figures 3D, E**). Consistently, enhanced p65 nuclear translocation was observed in *Rab29*-KO cells under both basal and GAS-infected conditions, indicating that Rab29 acts as a negative regulator of NF-κB activation during bacterial infection and basal conditions, potentially counteracting the NF-κB-promoting effects of TBC1D9 (**Figures 3F–I**). These findings indicate that TBC1D9 enhances IL-6 production via GAP-dependent NF-κB activation, while Rab29 opposes this effect by suppressing NF-κB signaling. Together, our data support a model in which the TBC1D9 regulates IL-6 expression through a Rab29-mediated pathway, balancing immune responses during bacterial infection.

### 3.4 Rab29 inhibits IL-6 expression in various bacterial infections

**Figure 4:**
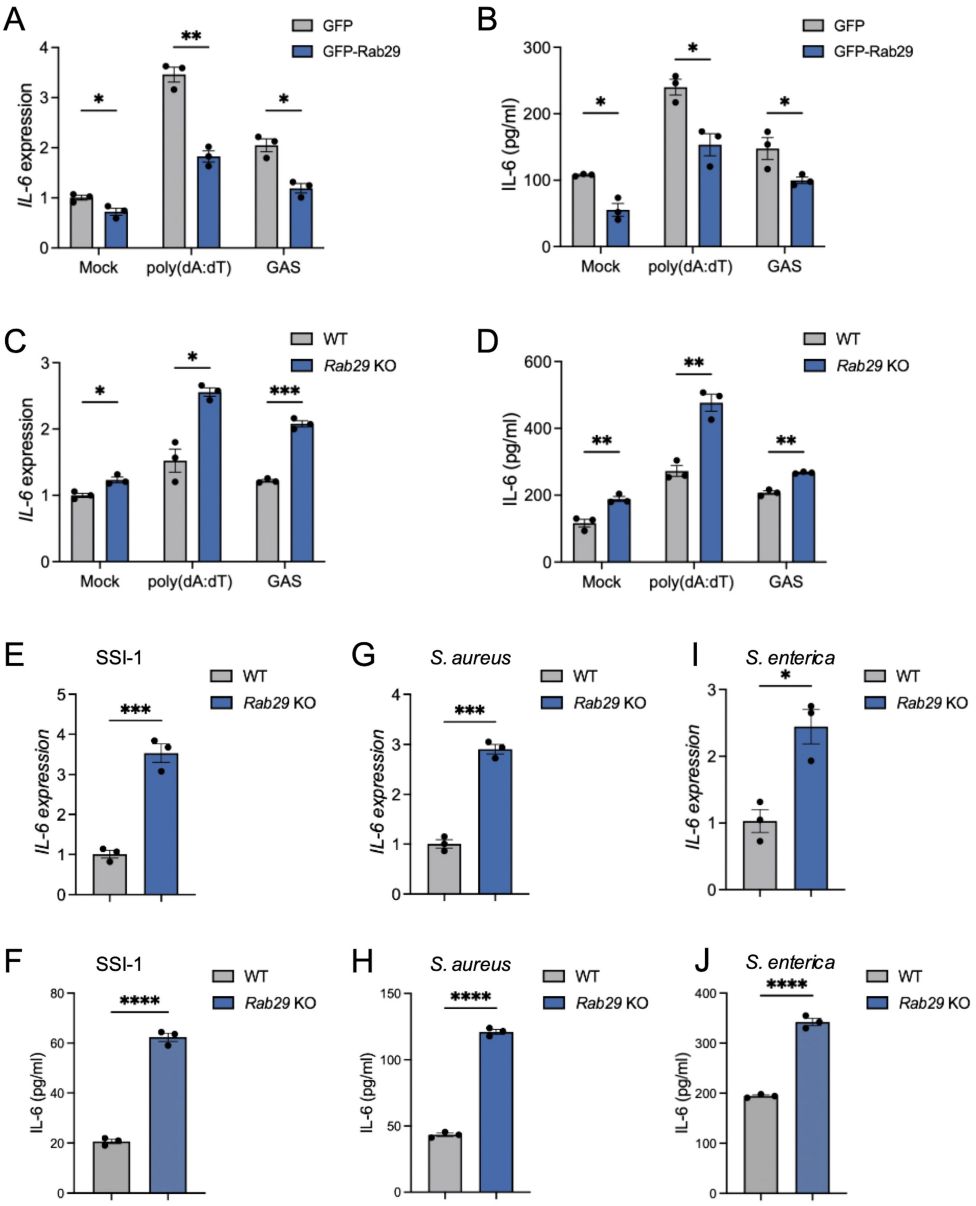
Rab29 inhibits IL-6 expression in various bacterial infections. HeLa cells (WT or *Rab29*-KO) were mock-treated, stimulated with 1 μg/mL poly(dA:dT) for 6 hours, or infected with GAS (JRS4), GAS SSI-1, *S. aureus*, or *S. enterica* (MOI 100) for 4 hours. qRT-PCR data were normalized to GAPDH and expressed as fold changes relative to mock-treated WT cells expressing GFP **(A, B)** or WT cells **(C–L)**. **(A-B)** IL-6 mRNA expression **(A)** and secretion **(B)** in cells overexpressing GFP or GFP-Rab29. **(C, D)** IL-6 mRNA expression **(C)** and secretion **(D)** in WT and *Rab29*-KO cells. **(E–J)** IL-6 mRNA expression and secretion in WT and *Rab29*-KO cells infected with GAS SSI-1 **(E, F)**, *S. aureus***(G, H)**, or *S. enterica***(I, J)**. Data are shown as mean ± SEM (n=3 independent experiments). Significance: ns (*p* ≥ 0.05), * (*p* < 0.05), ** (*p* < 0.01), *** (*p* < 0.001), **** (*p* < 1×10^-4^), determined by two-tailed Student’s t-test.

Building on how the TBC1D9-Rab29 axis in IL-6 regulation, we examined whether Rab29 acts as a negative regulator of IL-6 expression. We overexpressed GFP-tagged Rab29 in HeLa cells (**Supplementary Figure S1C**) and measured IL-6 levels after stimulation with poly(dA:dT) or GAS infection. Rab29 overexpression decreased IL-6 mRNA levels by 1.89-fold after poly(dA:dT) stimulation and 1.72-fold after GAS infection (*p* < 0.05; **Figure 4A**). Similarly, IL-6 protein secretion decreased by 40% and 42% under these conditions, respectively, compared to WT controls (*p* < 0.05; **Figure 4B**). Conversely, *Rab29*-KO cells exhibited a 1.6-fold increase in IL-6 mRNA following poly(dA:dT) stimulation, and GAS infection resulted in a 1.72-fold enhancement in IL-6 transcription (*p<*0.05, **Figure 4C**). Additionally, IL-6 levels increased subsequent to both poly(dA:dT) stimulation and GAS infection (**Figure 4D**). These results confirm that Rab29 suppresses IL-6 production in response to poly(dA:dT) stimulation and GAS infection.

To determine whether Rab29’s negative regulation also applies to other bacterial pathogens, we infected *Rab29*-KO HeLa cells with SSI-1, *S. aureus*, or *S. enterica*. In each case, *Rab29*-KO cells exhibited significantly higher IL-6 mRNA and protein levels compared to WT controls (*p* < 0.05; **Figures 4E–J**), suggesting that Rab29 has a broad regulatory effect in reducing IL-6 expression across various bacterial infections.

Together, these results indicate that Rab29 functions as a key negative regulator of IL-6 production, reducing both mRNA and protein levels in response to cytosolic DNA and various bacterial pathogens.

### 3.5 TBC1D9 selectively interacts with GTP-Rab29 and colocalizes in specialized subcellular compartments

**Figure 5:**
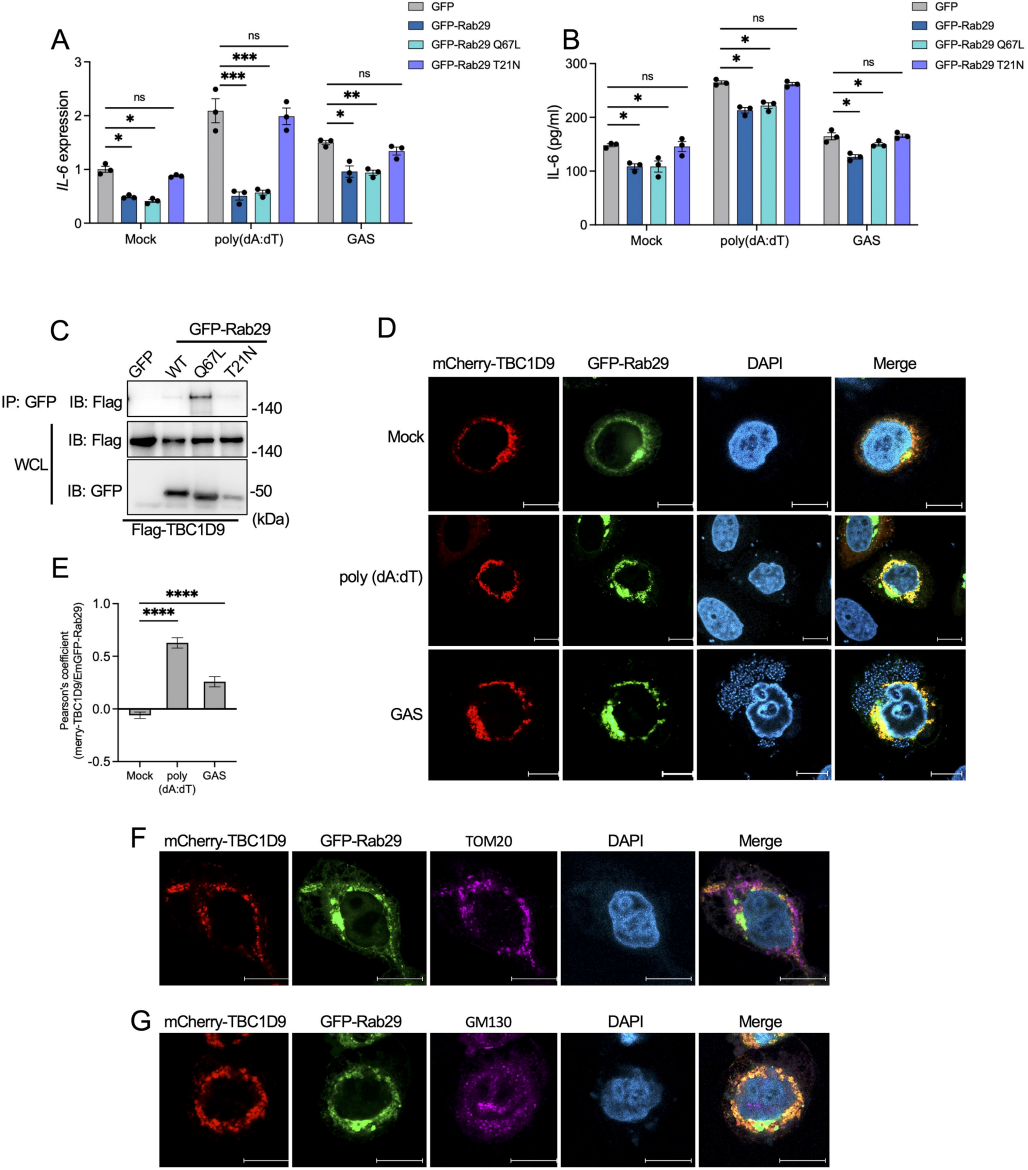
TBC1D9 selectively interacts with GTP-bound form Rab29 to modulate IL-6 production. *Rab29*-KO HeLa cells expressing GFP vector, GFP-Rab29, GFP-Rab29 Q67L, or GFP-Rab29 T21N were mock-treated, stimulated with 1 μg/mL poly(dA:dT) for 6 hours, or infected with GAS (JRS4) for 4 hours. **(A, B)** IL-6 mRNA expression **(A)** and secretion **(B)**. qRT-PCR data were normalized to GAPDH and expressed as fold changes relative to mock-treated *Rab29*-KO cells expressing GFP. **(C)** Co-immunoprecipitation (IP) of Flag-tagged TBC1D9 with GFP-tagged Rab29 mutants (GFP vector, WT, Q67L, T21N) in HeLa cells under baseline conditions. Input and IP samples were probed with GFP-trap beads and anti-Flag antibodies. **(D)** Representative confocal microscopy images showing the distribution of mCherry-TBC1D9 (red) and GFP-Rab29 (green). Scale bar: 10 µm. **(E)** Pearson’s correlation coefficient between mCherry-TBC1D9 and GFP-Rab29. **(F)** Representative confocal microscopy images showing the distribution of mCherry-TBC1D9 (red), GFP-Rab29 (green), and TOM20 (purple). Scale bar: 10 µm. **(G)** Representative confocal microscopy images showing the distribution of mCherry-TBC1D9 (red), GFP-Rab29 (green), and GM130 (purple). Scale bar: 10 µm. Data are shown as mean ± SEM (n=3 independent experiments). Pearson’s correlation coefficient was quantified from >20 images per condition **(E)**. Significance: ns (*p* ≥ 0.05), * (*p* < 0.05), ** (*p* < 0.01), *** (*p* < 0.001), **** (*p* < 1×10^-4^), determined by two-tailed Student’s t-test.

To assess whether Rab29 controls IL-6 production based on its GTP activity, we performed complementary experiments in *Rab29*-KO cells with GFP-Rab29 WT and two mutants: Q67L (constitutively active GTP-bound) and T21N (dominant-negative GDP-bound) (Zhu et al., 2023). Complementation with Rab29 WT reduced IL-6 mRNA levels under basal conditions (*p* < 0.05; **Figure 5A**). The Rab29 Q67L further decreased IL-6 mRNA by 3.67-fold, while Rab29 T21N had little effect (*p* < 0.05; **Figure 5A**). Similarly, IL-6 protein secretion followed this pattern, with Rab29 Q67L showing the strongest suppression compared to Rab29 WT and T21N under basal conditions (*p* < 0.05; **Figure 5B**). However, *in vitro* GTPase assays using recombinant GST-Rab29 and GST-TBC1D9 showed no significant increase in GTP hydrolysis upon co-incubation (**Supplementary Figure S5A**), suggesting TBC1D9 does not act as a GAP for Rab29. These data suggest that Rab29 regulates IL-6 production in a GTP-dependent manner, but this regulation is independent of TBC1D9’s GAP activity.

To investigate the TBC1D9-Rab29 interaction, we performed co-immunoprecipitation (co-IP) experiments in HeLa cells co-expressing Flag-tagged TBC1D9 and GFP-tagged Rab29 (GFP vector, WT, Q67L, or T21N). TBC1D9 exhibited a strong binding affinity for GTP-bound Rab29 Q67L, with weaker interactions with Rab29 WT and minimal binding to T21N under basal conditions (**Figure 5C**). Similarly, both poly(dA:dT) stimulation and GAS infection enhanced TBC1D9-Rab29 co-precipitation, with the highest increase observed for Rab29 Q67L (**Supplementary Figure S6A-B**). Furthermore, confocal microscopy of HeLa cells co-transfected with mCherry-tagged TBC1D9 and GFP-tagged Rab29 revealed significant colocalization after stimuli, as showed by Pearson’s correlation coefficient (**Figures 5D, E**). These data indicate that TBC1D9 preferentially binds to the GTP-bound form of Rab29, enhancing their interaction under inflammatory stimuli to regulate IL-6 production through a non-canonical mechanism.

Given the roles of Rab GTPases and TBC proteins in membrane trafficking, we hypothesized that the TBC1D9-Rab29 axis may coordinates IL-6 regulation within specific subcellular compartments. TBC1D9 is implicated in mitophagy and associates with mitochondria (Nozawa et al., 2020), while both TBC1D9 and Rab29 have been observed at the Golgi apparatus (Nakamura et al., 2015; Purlyte et al., 2018). Due to GAS infection disrupting Golgi structure (Nozawa et al., 2021), we assessed the subcellular localization of TBC1D9 and Rab29 following poly(dA:dT) stimulation. Colocalization observations revealed minimal overlap with neither mitochondria (**Figure 5F**) nor Golgi (**Figure 5G**), suggesting that TBC1D9-Rab29 interaction occurs within transient or specialized subcellular compartments, potentially facilitating the assembly of IL-6 regulatory complexes.

Together, these findings suggest that TBC1D9 specifically binds GTP-bound Rab29 to regulate IL-6 production by assembling signaling complexes in specialized subcellular compartments, independent of GAP catalysis. This localization-dependent process complements the homeostatic regulation of NF-κB and supports a physical association between TBC1D9 and Rab29, but does not specify the binding interface.

### 3.6 TBC1D9 regulates IL-6 production independently of Arid5a and mRNA stability

**Figure 6:**
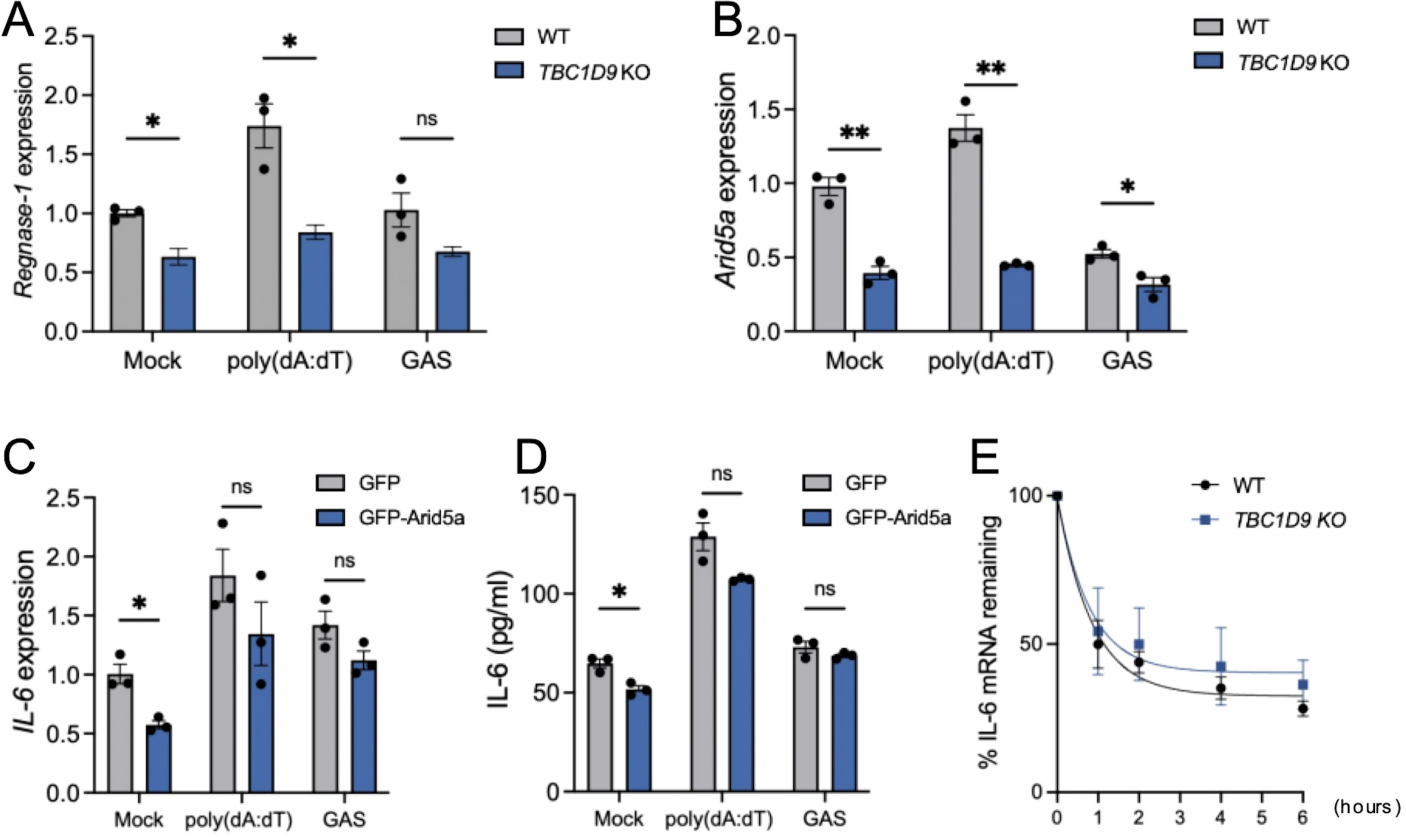
TBC1D9 regulates IL-6 production independently of Arid5a and mRNA stability. HeLa cells (WT or *TBC1D9*-KO) were mock-treated, stimulated with 1 μg/mL poly(dA:dT) for 6 hours, or infected with GAS (JRS4) for 4 hours. qRT-PCR data were normalized to GAPDH and expressed as fold changes relative to mock-treated WT cells **(A, B)** or *TBC1D9*-KO cells expressing GFP **(C, D)**. **(A, B)** mRNA expression of Regnase-1 **(A)** and Arid5a **(B)**. **(C, D)** IL-6 mRNA expression **(C)** and secretion **(D)** in *TBC1D9*-KO cells expressing GFP or GFP-Arid5a. **(E)** IL-6 mRNA levels in WT and *TBC1D9*-KO cells after actinomycin D treatment, normalized to GAPDH and expressed relative to 0 hour as 100%. Data are shown as mean ± SEM (n=3 independent experiments). Significance: ns (*p* ≥ 0.05), * (p < 0.05), ** (p < 0.01), determined by two-tailed Student’s t-test.

Given the specifically reduced IL-6 expression in *TBC1D9*-KO cells despite unchanged stimulus-induced NF-κB activity, we hypothesized that TBC1D9 regulates IL-6 at the post-transcriptional level. We examined key IL-6 mRNA regulatory factors and found that Regnase-1 mRNA levels were significantly decreased in *TBC1D9*-KO cells under basal conditions, after poly(dA:dT) stimulation, and during GAS infection (*p* < 0.05, **Figure 6A**). Since Regnase-1 promotes degradation of IL-6 mRNA, this decrease indicates that TBC1D9 does not increase IL-6 expression by inhibiting Regnase-1. However, Arid5a mRNA, which stabilizes IL-6 mRNA, was reduced in *TBC1D9*-KO cells by 3.05-fold after poly(dA:dT) stimulation and 1.65-fold after GAS infection (*p<*0.05; **Figure 6B**). To determine whether Arid5a mediates the effect of TBC1D9 on IL-6, we expressed GFP-Arid5a in *TBC1D9*-KO cells. However, Arid5a overexpression failed to restore IL-6 mRNA or protein levels after poly(dA:dT) stimulation or GAS infection (*p* > 0.05, **Figures 6C, D**), suggesting that Arid5a is not a primary effector of TBC1D9-mediated IL-6 regulation. To evaluate post-transcriptional regulation, we measured IL-6 mRNA stability using actinomycin D (Act. D) chase assays. The half-life of IL-6 mRNA (t_½_) was about 1 hour in both wild-type and *TBC1D9*-KO cells (**Figure 6E**), indicating that TBC1D9 does not directly affect mRNA stability. These results suggest that TBC1D9 controls IL-6 expression through a distinct mechanism, independent of Arid5a and mRNA stability, implying the involvement of other unidentified factors within a broader regulatory network.

### 3.7 Transcriptomic profiling connects TBC1D9 to IL-6–mediated inflammation in NSTIs

Given that TBC1D9 selectively regulates IL-6 expression in epithelial cells during cytosolic DNA sensing and bacterial infection, we then aimed to see if this regulatory pathway is also relevant in human disease. NSTI is a severe, rapidly progressing bacterial infection. Its microbial cause can be either monomicrobial or polymicrobial. In cases caused by a single pathogen, GAS is the main bacteria involved, while other bacteria are less commonly reported (Thänert et al., 2019). To determine whether the TBC1D9-IL-6 axis identified in our cell-based experiments is also relevant in human disease, we examined publicly available RNA-seq datasets from NSTI patients (PRJNA479582) (Thänert et al., 2019) and healthy controls (GSE249027). Differential expression analysis identified 29,138 genes with significantly altered profiles (*p* < 0.05, |log_2_FC| ≥ 2). Among the upregulated genes, TBC1D9 was significantly elevated in NSTI tissue (**Supplementary Figure S7A**). Gene set enrichment analysis (GSEA) further demonstrated the enrichment of acute inflammatory pathways, with IL-6 signaling prominently featured (**Supplementary Figures S7B, C**). Notably, IL-6 and its downstream chemokines were markedly expressed in NSTI samples. These data suggest that the TBC1D9-IL-6 regulatory axis identified *in vitro* is also active in the pathophysiological context of NSTI, reinforcing GAS as both a mechanistic driver in our studies and the main clinical pathogen causing severe soft tissue infections.

Together, TBC1D9 enhances IL-6 expression in epithelial cells during cytosolic DNA sensing and various bacterial infections, sustaining homeostatic NF-κB activity but not stimulus-induced activation. Conversely, Rab29 suppresses IL-6 production and NF-κB signaling in a GTP-dependent manner. TBC1D9 selectively binds GTP-bound Rab29 to regulate IL-6 through signaling complex assembly in specialized subcellular compartments. The TBC1D9-Rab29 axis defines a regulatory mechanism and a potential therapeutic target for reducing excessive inflammation in bacterial infections and inflammatory diseases.

## 4 Discussion

**Figure 7:**
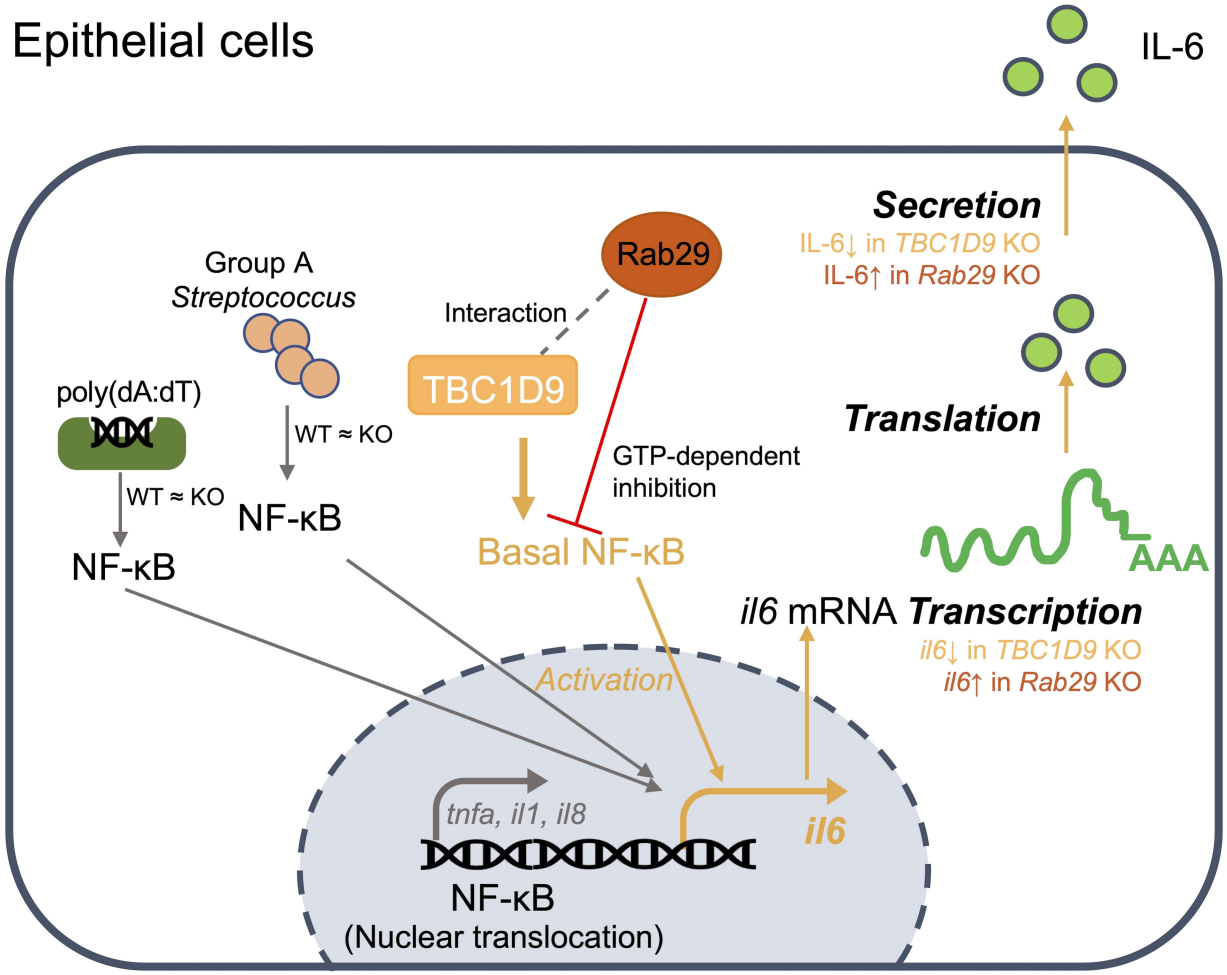
Model of the TBC1D9-Rab29 axis in epithelial IL-6 regulation: In epithelial cells, TBC1D9 interacts with Rab29 (gray dashed line; MS and co-IP) and maintains basal NF-κB in the nucleus, thereby promoting IL6 transcription and subsequent translation and secretion (orange path). Stimulus-induced NF-κB nuclear translocation by poly(dA:dT) or GAS is unchanged between WT and TBC1D9-KO (“WT ≈ KO”). Rab29 provides a GTP-dependent inhibition of this axis (red ⊥). Pro-inflammatory genes such as TNF-α/IL-1β/IL-8 are not positively regulated by TBC1D9 (gray branch). TBC1D9 KO would decrease IL6 production, while Rab29 KO would increase IL6 production at the mRNA and secretion levels.

In cytosolic DNA sensing and bacterial infection, selective IL-6 production in epithelial cells is controlled by complementary mechanisms. At the signaling level, TBC1D9 maintains homeostatic NF-κB activity to specifically regulate IL-6 production, while GTP-bound Rab29 counteracts this process. In parallel, these two proteins co-localize and organize signaling complexes within specialized subcellular compartments, creating a localization-dependent process that further activates NF-κB signaling. At the host-pathogen interface, this integrated regulation controls selective IL-6 production, which may limit hyperinflammation without weakening antibacterial defenses (**Figure 7**).

Previous studies suggest that TBC1D9 regulates TBK1 activation during xenophagy and mitophagy by ubiquitin recognition (Nozawa et al., 2020). However, its contribution to cytokine regulation remained unexplored. Our study identifies TBC1D9 as a selective regulator of IL-6 expression in epithelial cells during innate immune responses to cytosolic DNA and bacterial infections. Specifically, TBC1D9 deficiency caused a marked reduction in IL-6 production in response to both poly(dA:dT) stimulation and GAS infection, while the other pro-inflammatory cytokines such as IFN-β, IL-8, IL-1β, and TNF-α, was increased. Furthermore, this selective suppression was consistent across epithelial cell types (HeLa and A549) and various bacterial pathogens (*S. aureus*, *S. enterica*, hypervirulent GAS SSI-1). These observations establish TBC1D9 as a conserved and functionally specific regulator of IL-6 expression in epithelial immunity.

TBC1D9 selectively maintains homeostatic NF-κB in epithelial cells to enhance IL-6 production. Consistent with stimulus- and cell type-specific transcriptional control among IL-6 family members, this setup directs the response toward IL-6 without causing a broad cytokine surge. Depletion of TBC1D9 decreases p-p65 under unstimulated conditions, paralleling the reduction in IL-6, supporting a model in which TBC1D9 maintains a homeostatic NF-κB level that primarily enables IL-6 expression. However, NF-κB activation remained stable after poly(dA:dT) stimulation or GAS infection, and IL-6 induction was unaffected by NF-κB inhibition under these conditions, suggesting that other pathways, such as AP-1 or C/EBPβ, likely compensate for the loss of NF-κB during immune activation. Previous studies have shown that p-p65 levels increase significantly in response to poly(dA:dT) stimulation, activating multiple downstream signaling pathways (Qiao et al., 2024). However, in our study, GAS infection triggered weaker NF-κB activation and lower p-p65 levels compared to poly(dA:dT) stimulation. The weakened response may result from various GAS components interacting with different immune pathways, along with previous findings suggesting limited p65 nuclear translocation and preferential activation of non-canonical NF-κB signaling in macrophages (Wu et al., 2016). Furthermore, since the phosphorylation of p65 at Ser536 is transient, measuring p-p65 at 4–6 hours (Kang et al., 2020), consistent with the detection of IL-6 levels, captures ongoing phosphorylation rather than initial events. IL-6 indicates the overall signaling timeline, which explains why there is a divergence despite similar levels of p-p65 at later time points. Although TBC1D9 activates TBK1 signaling and TBK1 could promote non-canonical NF-κB signaling via TRAF2 complexes to produce IL-6, TBK1 was not essential for TBC1D9-mediated IL-6 production in our study (**Supplementary Figures S8A, B**), indicating a TBK1-independent mechanism (Balka et al., 2020). Our data show that TBC1D9 maintains IL-6 expression by supporting homeostatic NF-κB activity and facilitating promoter binding at the IL-6 locus. When this baseline is reduced (*TBC1D9*-KO), the transcriptional output per unit of inducible NF-κB signal diminishes, so IL-6 stays low even after stimulation, while strongly inducible promoters like IL-1β and TNF-α remain mostly unaffected and continue to increase (Bhatt and Ghosh, 2014). Consistently, brief BAY 11–7082 pretreatment followed by washout selectively and sustainably suppresses resting IL-6 while preserving the ability to induce IL-6 upon subsequent stimulation (**Figures 2H, I**), indicating that homeostatic NF-κB activity is required to maintain IL-6 at rest. Post-transcriptional regulation of IL-6 typically involves Arid5a and Regnase-1 (Metwally et al., 2020). Although *TBC1D9*-KO cells showed reduced Arid5a expression, Arid5a overexpression failed to restore IL-6 levels. Furthermore, IL-6 mRNA stability remained unchanged between WT and *TBC1D9*-KO cells. These findings eliminate Arid5a and mRNA stability as the main factors.

Mass spectrometry analysis of Rab GTPases in WT and GAP-deficient (R566K) TBC1D9-overexpressing HEK293T cells identified Rab29 as a unique suppressor of NF-κB activity, unlike other Rab GTPases that enhance NF-κB signaling and IL-6 expression. For instance, Rab7b has been showed to enhance NF-κB signaling and IL-6 expression (He et al., 2011), while Rab5a deficiency reduces NF-κB activation and diminishes IL-6 production in macrophages (Chen et al., 2020). Studies have shown that the vesicle transport function of Rab29 is involved in T cell activation and that T cells lacking Rab29 display a defective tyrosine phosphorylation cascade, resulting in reduced IL-2 expression (Onnis et al., 2015). While, in our study, Rab29 inhibited IL-6 production in a GTP-dependent manner, with the GTP-bound Q67L mutant showing stronger suppression than Rab29 WT. These studies show Rab GTPase diversity in innate immunity and identify Rab29 as an unusual suppressor that may counteract pro-inflammatory signaling within the TBC1D9 regulation axis. The canonical GAP substrates of TBC1D9, remain unknown; nevertheless, it has been shown to interact with Rab7 and Rab9 (Metwally et al., 2020). Although TBC1D9 lacks GAP activity towards Rab29, the GTP-bound form of Rab29 binds to TBC1D9 and increases IL-6 production, indicating a non-catalytic functional interaction. The GTP-dependent co-precipitation (Q67L≫WT≈T21N) shows a preference for binding to GTP-bound Rab29 under both basal and stimulus conditions. We refine our conclusion and describe a “TBC1D9-Rab29 axis” as a potential target for anti-inflammation. This TBC1D9-Rab29 axis, with opposing effects on NF-κB signaling, connects GTPase signaling and membrane trafficking to cytokine regulation in epithelial innate immunity. Our results enhance understanding of Rab GTPase networks in inflammatory signaling, underscoring the TBC1D9-Rab29 axis as a key link between membrane trafficking and immune regulation. Further research is necessary to clarify the molecular details and organization of this pathway during inflammation.

NSTIs are life-threatening bacterial infections characterized by rapidly spreading necrosis of the skin and subcutaneous tissues. The microbial etiology of NSTIs can be monomicrobial or polymicrobial. Among monomicrobial cases, GAS is the predominant pathogen, whereas other streptococcal species (*S. dysgalactiae*, *S. agalactiae*) and *S. aureus* are reported less frequently. In contrast, polymicrobial NSTIs often involve mixed aerobic and anaerobic bacteria, including Enterobacteriaceae, *Bacteroides* spp., and *Clostridium* spp (Thänert et al., 2019). Our transcriptomic analysis of NSTIs patient biopsies provides direct clinical evidence supporting the mechanistic findings from epithelial cell models. TBC1D9 was significantly increased in NSTIs and was associated with activation of IL-6-related inflammatory pathways, emphasizing its role in promoting hyperinflammation. Because GAS is the predominant cause of monomicrobial NSTIs, our GAS infection model closely reflects the primary clinical presentation of this disease. The consistency between mechanistic data and patient transcriptomics identifies TBC1D9 as a specific regulator of IL-6 production in the pathophysiological context of GAS-associated NSTIs, extending our study from cellular models to human diseases.

There are some limitations in our study. The use of HeLa and A549 cells as primary models and the limited range of pathogens tested may inadequately reflect the intricate complexity of *in vivo* immune responses. Additionally, although our data clearly shows that the TBC1D9-Rab29 axis is involved in IL-6 regulation, the exact molecular mechanisms still need further clarification. Furthermore, the way TBC1D9-Rab29 regulates IL-6 remains to be determined, and identifying the interface will be necessary. Also, the clinical relevance was evaluated using bulk transcriptomic data from NSTIs tissue, which does not differentiate the cell-type-specific contributions of TBC1D9. In future studies, we plan to clarify these mechanisms by validating key phenotypes in primary epithelial cultures and targeted *in vivo* models, as well as expanding to include viral and non-bacterial innate immune stimuli.

In conclusion, our study identifies a new TBC1D9-Rab29 axis that manages homeostatic NF-κB activity and specifically controls IL-6 production in epithelial cells. This axis connects GTPase signaling with cytokine regulation, indicating a coordinated mechanism driving epithelial innate immunity. Instead of changing the overall level of immune activation, this axis adjusts specific parts of the response, reducing excessive inflammation while maintaining antimicrobial defense, and provides a potential target for precise immunomodulation during infection.

## Data Availability

The datasets presented in this study can be found in online repositories. The datasets presented in this study can be found in online repositories. The MS proteomics data used here were deposited in jPOSTrepo (a ProteomeXchange consortium repository) with dataset ID JPST004116: https://repository.jpostdb.org/entry/JPST004116. All other study data are included in the article and/or **Supplementary Material**.
