# Supplementary material for: A TBC1D9-Rab29 axis controls homeostatic NF-κB signaling and selective IL-6 production in epithelial cells
Source: Front Cell Infect Microbiol. 2025 Nov 11;15:1688013. doi: 10.3389/fcimb.2025.1688013 (PMC12643979; doi:10.3389/fcimb.2025.1688013)
Supplement: Supplementary file 3 [file DataSheet3.docx]

***Supplementary Material for***

A TBC1D9-Rab29 axis controls homeostatic NF-κB signaling and selective IL-6 production in epithelial cells

Xin Hu^a^, Takashi Nozawa^a*^, Atsuko Minowa-Nozawa^a^, Kazunori Murase^a^, Ichiro Nakagawa^a*^

*Correspondence:

nozawa.takashi.4r@kyoto-u.ac.jp (T.N.);

nakagawa.ichiro.7w@kyoto-u.ac.jp (I.N.)

This file includes:

1. Figures S1 to S8
2. Tables S1 to S3
3. Other supporting materials for this manuscript include the following:
4. Data S1 to S2
5. Raw WB images
6. **Supplementary Figures**


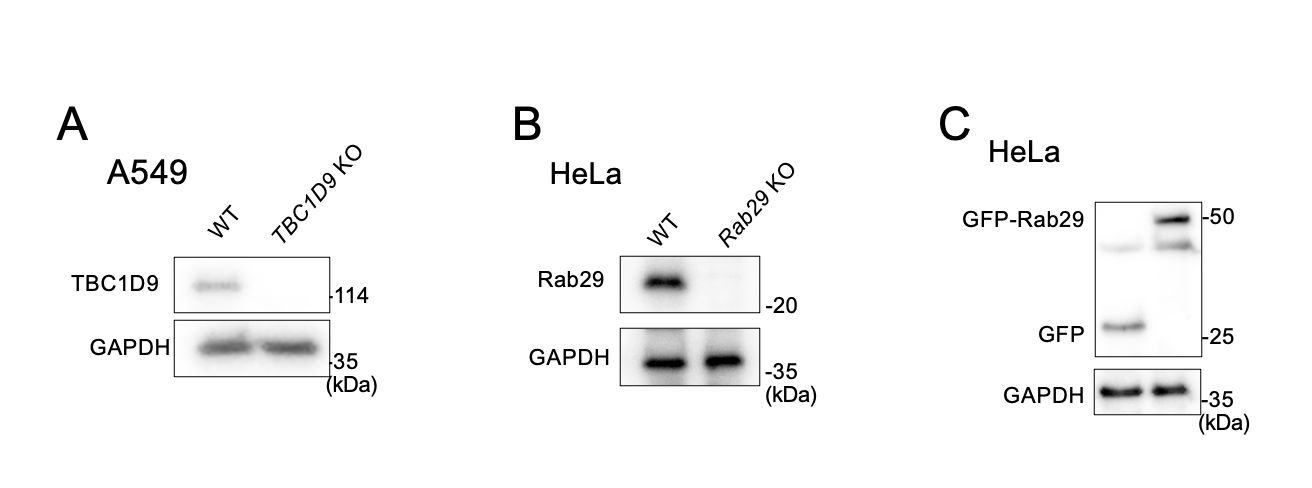


Figure S1: Validation of KO cell lines. (A) TBC1D9-KO in A549 cells by Western blot. GAPDH serves as a loading control. (B) Rab29-KO in HeLa cells by Western blot. GAPDH serves as a loading control. (C) GFP–Rab29 validation by Western blot using the same lysates as in the functional assays. GAPDH serves as a loading control.


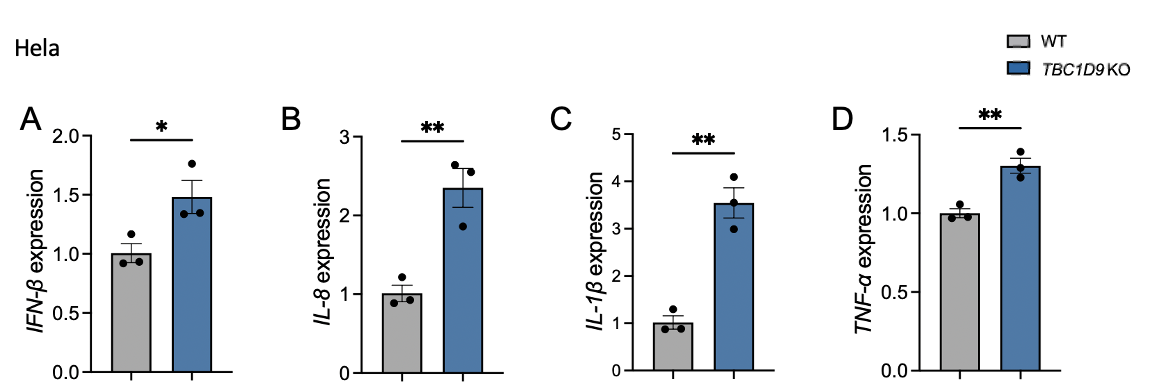


Figure S2: Hypervirulent GAS (SSI-1) does not significantly increase pro-inflammatory cytokines in HeLa cells in a TBC1D9-dependent manner: HeLa cells (WT or TBC1D9-KO) were infected with GAS SSI-1 (M3; MOI 100) for 4 hours. qRT–PCR was performed for IFN-β (A), IL8 (B), IL1-β (C), and TNF-α (D).

Data are shown as mean ± SEM (n=3 independent experiments). Significance: ns (p ≥ 0.05), * (p < 0.05), **** (p < 1×10⁻⁴), determined by two-tailed Student’s t-test.


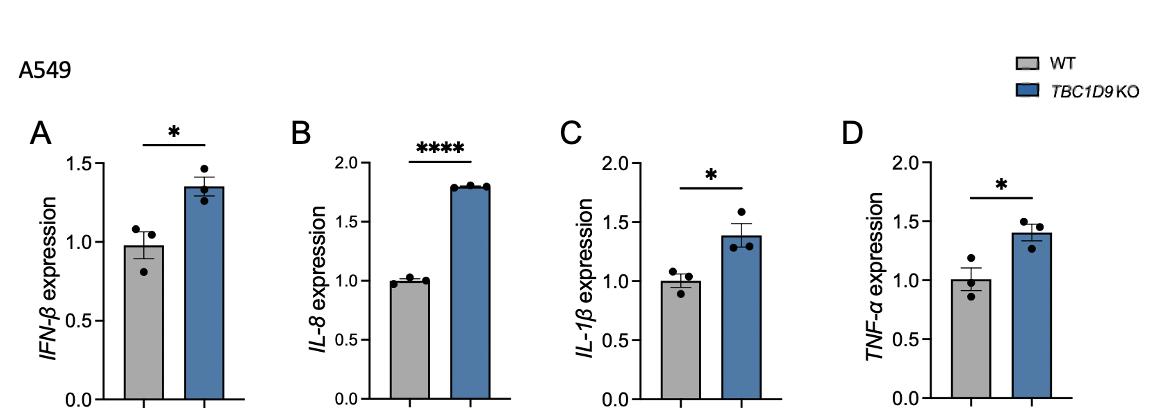


Figure S3: Hypervirulent GAS (SSI-1) does not significantly increase pro-inflammatory cytokines in A549 cells in a TBC1D9-dependent manner: A549 cells (WT or TBC1D9-KO) were infected with GAS SSI-1 (M3; MOI 100) for 4 hours. qRT–PCR was performed for IFN-β (A), IL8 (B), IL1-β (C), and TNF-α (D).

Data are shown as mean ± SEM (n=3 independent experiments). Significance: ns (p ≥ 0.05), * (p < 0.05), **** (p < 1×10⁻⁴), determined by two-tailed Student’s t-test.


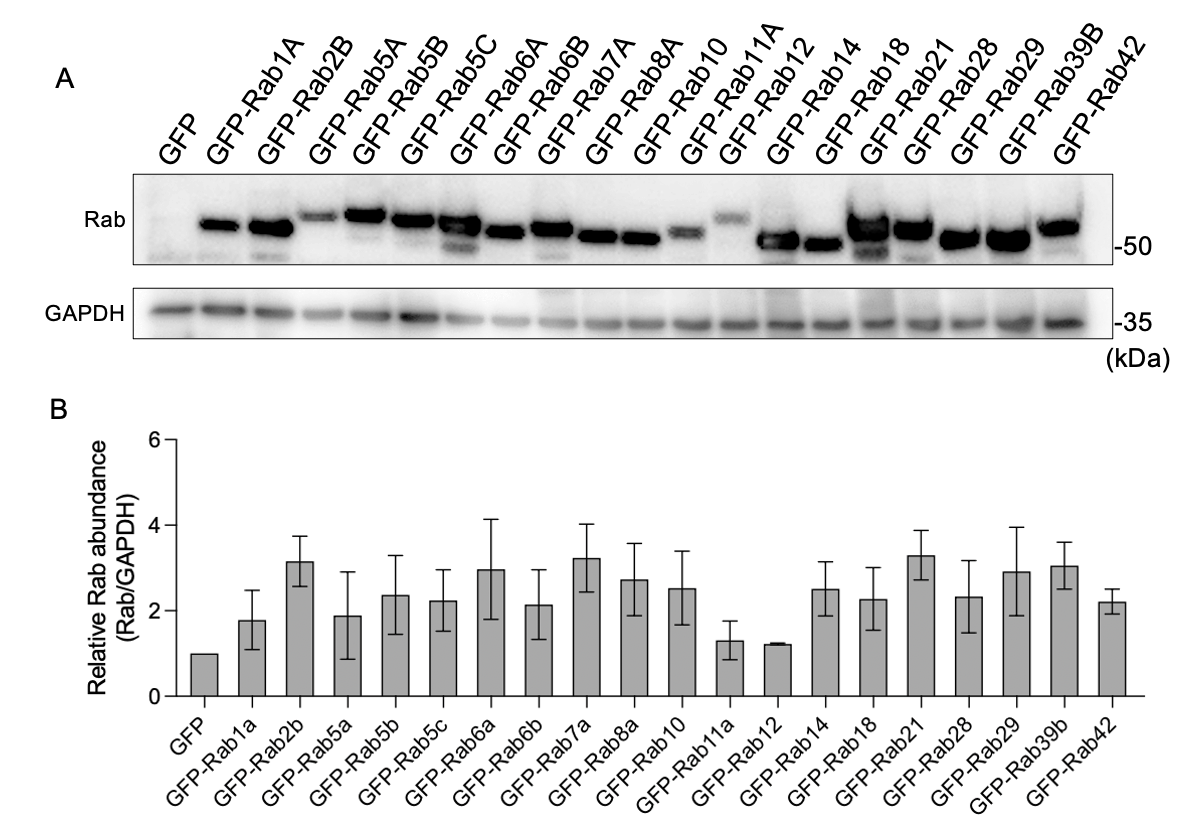


Figure S4: Quality control for Rab expression.

(A) GFP-Rab validation by Western blot using the same lysates as in the functional assays; GAPDH serves as a loading control; (B) Densitometric quantification of Rab abundance (Rab/GAPDH, normalized to GFP control); data are mean ± SEM from n = 3 independent experiments.


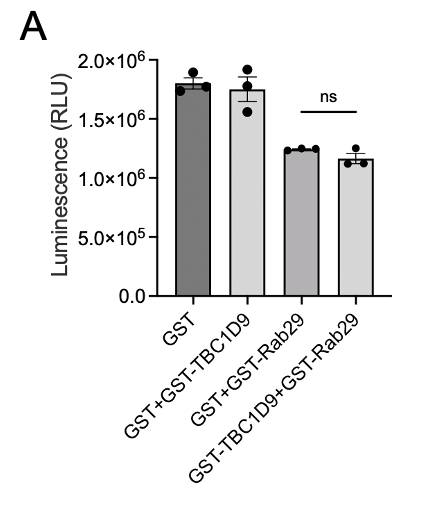


Figure S5 (A) GTP hydrolyzing activity between GST-TBC1D9 and GST-Rab29. Data are shown as mean ± SEM (n=3 independent experiments). Significance: ns (p ≥ 0.05), determined by two-tailed Student’s t-test.


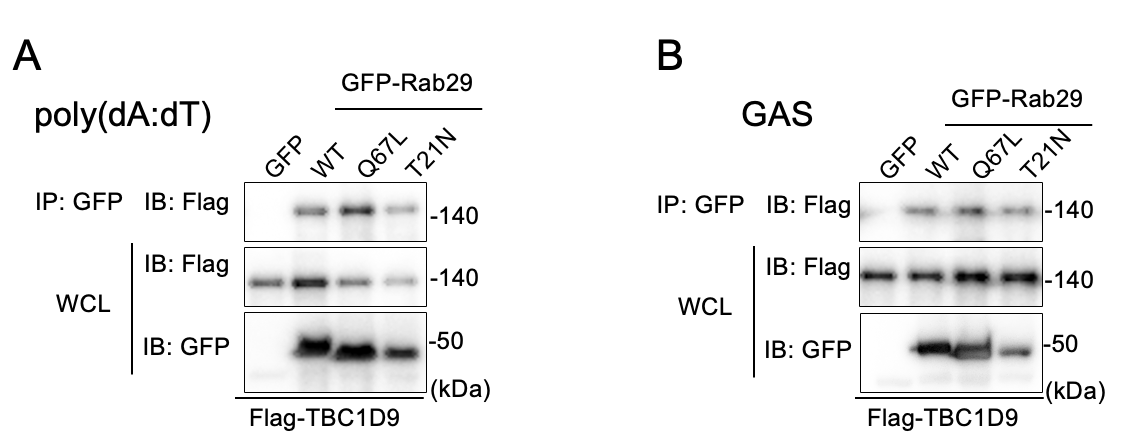


Figure S6: Stimulus- and infection-enhanced TBC1D9-Rab29 interaction.

HeLa cells co-expressing Flag-TBC1D9 and GFP-Rab29 mutants (GFP vector, WT, Q67L, T21N) were stimulated with poly(dA:dT) (1 µg/mL, 6 h) (A) or infected with GAS (MOI 100, 4 h) (B).


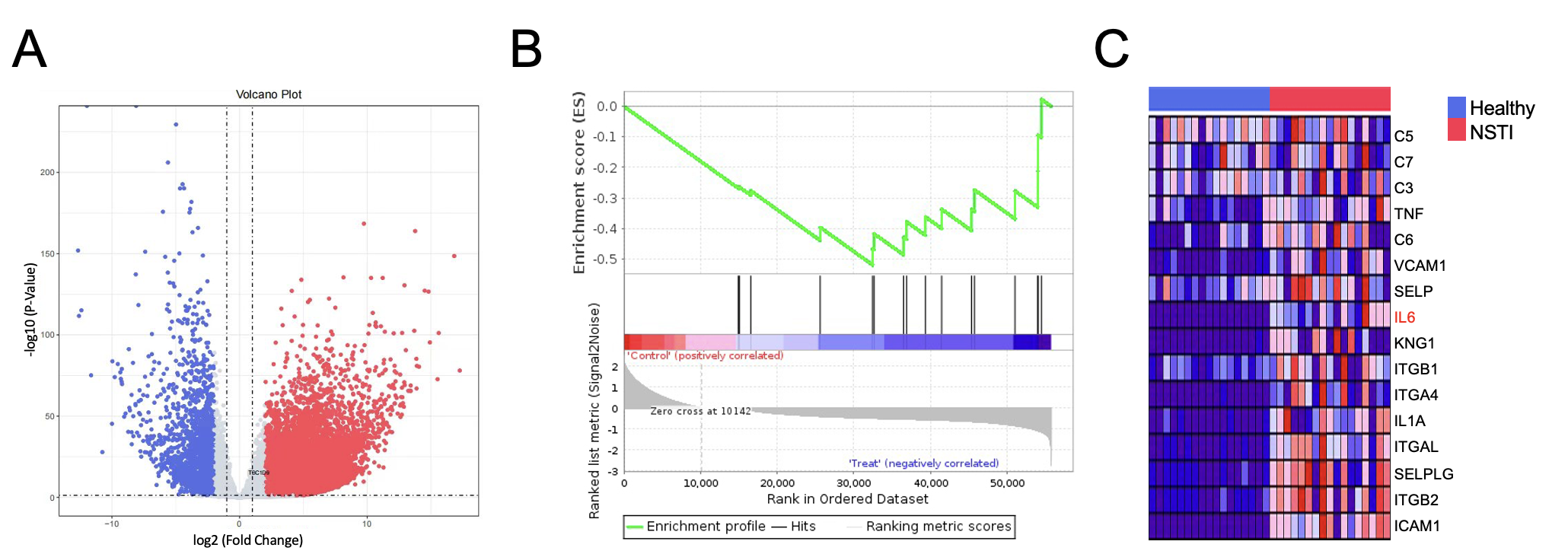


Figure S7. Transcriptomic profiling of NSTIs patients.

(A) Volcano plot showing differentially expressed genes between healthy controls and NSTIs patients. Red and blue dots indicate significantly upregulated (log₂FC ≥ 2, p < 0.05) and downregulated (log₂FC ≤ −2, p < 0.05) genes.

(B) Running enrichment score (green) for the gene set WP_CELLS_AND_MOLECULES_INVOLVED_IN_LOCAL_ACUTE_INFLAMMATORY_RESPONSE; black ticks mark gene positions, and the segment at the ES peak denotes the leading-edge subset (blue = high expression in NSTIs patients; red = high expression in healthy controls; scale indicated).

(C) Heatmap of leading-edge gene expression from (B). Columns are grouped by condition; genes are ordered by their rank in the enrichment analysis (rank in gene list) within the leading-edge subset. IL6 is highlighted in red to underscore its role in enrichment.


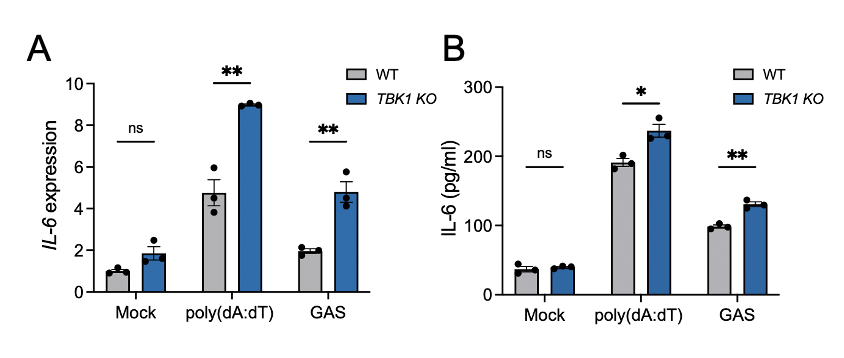


Figure S8: TBC1D9 regulates IL-6 production independently TBK1 pathway.

(A-B) IL-6 mRNA expression(A) and IL-6 levels(B) in HeLa cells (WT, *TBK1*-KO) were mock-treated, stimulated with poly(dA:dT) for 6 hours, or infected with GAS for 4 hours. qRT-PCR data were normalised to GAPDH and expressed as fold changes relative to mock-treated WT cells.

Data are shown as mean ± SEM (n=3 independent experiments). Significance: ns (p ≥ 0.05), * (p < 0.05), ** (p < 0.01), determined by two-tailed Student’s t-test.

1. **Supplementary Tables**

| Table S1. Bacterial strains and plasmids used in this study. | |  |
| --- | --- | --- |
| Stains | Relevant genotype | Source |
| *Streptococcus pyogenes* | |  |
| JRS4 | *Streptococcus pyogenes* str. JRS4, serotype M6 | Laboratory stock |
| SSI-1 | *Streptococcus pyogenes* str. SSI-1, serotype M3 | Laboratory stock |
| *Staphylococcus aureus* | |  |
| KUH180129 | *Staphylococcus aureus* str. KUH180129 | PMCID: PMC6872886 |
|  |  |  |
| *Salmonella enterica* |  |  |
| LT2 | *Salmonella enterica Typhimurium* str. LT2 | PMID: 30428163 |
|  |  |  |
| *Escherichia coli* | |  |
| DH10B | *Escherichia coli* str. DH10B | Invitrogen, Cat# EC0113 |
| iVEC3 | *Escherichia coli* str. SN1187 (iVEC3) | NBRP, Cat# ME9806 |
| DE3 | *Escherichia coli* str. BL21(DE3) | PMID: 32034138 |

| \| Table S2. Primers used in this study \| \| --- \| | | | |  |
| --- | --- | --- | --- | --- | --- |
| Gene | Primer Sequence | | | Reference |
| For construction Recombinant DNA | | | | |
| Rab2b | Forward: 5’- CACCATGACTTATGCTTATCTCTTCAAGTATATCAT -3’ | | | This study |
|  | Reverse: 5’- ATGTTCAGCAGCAGCCAGAGTTGGACCCTATGTCAC -3’ | | |  |
| Rab6a | Forward: 5’- CACCATGTCCACGGGCGGAGACTTCGGGAATCCGCT -3’ | | | This study |
|  | Reverse: 5’- GAGATTAGCAGGAACAGCCTCCTTCACTGACTGGTT -3’ | | |  |
| Rab6b | Forward: 5’- CACCATGTCCGCAGGGGGAGATTTTGGGAATCCACT -3’ | | | This study |
|  | Reverse: 5’- TGCATTAGCAGGAGCAGCCGCCCTCGCTGGCCGGGG -3’ | | |  |
| Rab12 | Forward: 5’- CACCATGGATCCGGGCGCCGCGCTGCAGAGGCGGGC -3’ | | | This study |
|  | Reverse: 5’- GAAATCAACAGCATCGGACATGTGGTC TTGGTGGAG -3’ | | |  |
| Rab28 | Forward: 5’- CACCATGTCGGACTCTGAGGAGGAGAGCCAGGACCG -3’ | | | This study |
|  | Reverse: 5’-GCGCTCACTGAACTGCACACATAGAGCTTCTAGGAG -3’ | | |  |
| Rab29 | Forward: 5’- CACCATGGGCAGCCGCGACCACCTGTTCAAAGTGCT -3’ | | | This study |
|  | Reverse: 5’- ACTACTAGCAGCAGGACCAGCTGGAGGACTTGGTTT-3’ | | |  |
| Rab42 | Forward: 5’- CACCATGGAGGCCGAGGGCTGCCGCTACCAATTTCG -3’ | | | This study |
|  | Reverse: 5’- TCAACACTGGCATGGGCCTGAGTGCTGCTTCCTGCT-3’ | | |  |
| Arid5a | Forward: 5’- GGAACCAATTCAGTCGACTGGATGGCAGCCCCTGTCAAA -3’ | | | This study |
|  | Reverse: 5’- GAAAGCTGGGTCTAGATATCTCAGCTTGGTGTTGAGGTGG -3’ | | |  |
|  | | |  |  |
| For construction of Rab29 point mutant | | | |  |
| Rab29 Q67L | | Forward: 5’- CAGGGCTGGAGCGCTTCA -3’ | | This study |
|  | | Reverse: 5’- CGCTCCAGCCCTGCAATA -3’ | |  |
| Rab29 T21N | | Forward: 5’- TGGGCAAGAATTCGCTGGTGCAGCGATATTCC -3’ | | This study |
|  | | Reverse: 5’-GACGCCGCAGGGGACCAGCGAATTCTTGCCCA -3’ | |  |
|  | |  | |  |
| FOR qRT-PCR | | | |  |
| IL-6 | | Forward: 5’- AGACAGCCACTCACCTCTTCAG -3’ | | This study |
|  | | Reverse: 5’- TTCTGCCAGTGCCTCTTTGCTG -3’ | |  |
| IFN-β | | Forward: 5’- CTTGGATTCCTACAAAGAAGCAGC -3’ | | This study |
|  | | Reverse: 5’- TCCTCCTTCTGGAACTGCTGCA -3’ | |  |
| IL-8 | | Forward: 5’- GAGAGTGATTGAGAGTGGACCAC -3’ | | This study |
|  | | Reverse: 5’- CACAACCCTCTGCACCCAGTTT -3’ | |  |
| IL-1β | | Forward: 5’- CCACAGACCTTCCAGGAGAATG -3’ | | This study |
|  | | Reverse: 5’- GTGCAGTTCAGTGATCGTACAGG -3’ | |  |
| TNF-α | | Forward: 5’- CTCTTCTGCCTGCTGCACTTTG -3’ | | This study |
|  | | Reverse: 5’-ATGGGCTACAGGCTTGTCACTC -3’ | |  |
| Regnase-1 | | Forward: 5’- TCCTGCGTAAGAAGCCACTCAC -3’ | | This study |
|  | | Reverse: 5’- GGTGGAAGAATCGGCACTTGATC -3’ | |  |
| Arid5a | | Forward: 5’- TGGCAAGCAGAACGGAATCCAG -3’ | | This study |
|  | | Reverse: 5’-CTTGTAGAGGCTGACCAGGAAG -3’ | |  |
| GAPDH | | Forward: 5’- GTCTCCTCTGACTTCAACAGCG -3’ | | This study |
|  | | Reverse: 5’- ACCACCCTGTTGCTGTAGCCAA -3’ | |  |

| Table S3. Antibodies used in this study. | |  | |  |
| --- | --- | --- | --- | --- |
| Antibodies | Source | | Identifier | Dilution |
| Anti-GAPDH | Santa Cruz Biotechnology | | Cat# sc-47724, RRID: AB_627678 | 1:1000 |
| Anti-TBC1D9 | Thermo Fisher Scientific | | Cat# A301-028A, RRID: AB_2199400 | 1:1000 |
| Anti-Rab29 | Abcam | | Cat# ab256526, RRID: AB_2884877 | 1:1000 |
| Anti-NF-kappaB p65 | Cell Signaling Technology | | Cat #8242, RRID: AB_10859369 | 1:1000 |
| Anti-Phospho-NF-κB p65 | Cell Signaling Technology | | Cat #3033, RRID: AB_331284 | 1:1000 |
| Anti-FLAG | Sigma-Aldrich | | Cat# F3165, RRID: AB_259529 | 1:1000 |
| Anti-GFP | Nacalai Tesque | | Cat# 04363-24, RRID: AB_3675836 | 1:1000 |
| Anti-GST | Nacalai Tesque | | Cat# 04435-84, RRID: AB_3697019 | 1:1000 |
| Anti-GM130 | BD Transduction Laboratories | | Cat# 610822, RRID: AB_398141 | 1:100 |
| Anti-TOM20 | Santa Cruz Biotechnology | | Cat# sc-17764, RRID: AB_628381 | 1:100 |
| Anti-Rabbit IgG, Alexa Fluor Plus 488 | Invitrogen | | Cat# A32790, RRID:AB_2762833 | 1:100 |
| Anti-Mouse IgG, Alexa Fluor 647 | Jackson ImmunoResearch Labs | | Cat# 115-605-003,  RRID: AB_2338902 | 1:200 |
| Peroxidase-Anti-Mouse IgG | Jackson ImmunoResearch Labs | | Cat# 715-035-151,  RRID: AB_2340771 | 1:5000 |
| Peroxidase-Anti-Rabbit IgG | Jackson ImmunoResearch Labs | | Cat# 711-035-152,  RRID: AB_10015282 | 1:5000 |
