# Supplementary figures and images for: A TBC1D9-Rab29 axis controls homeostatic NF-κB signaling and selective IL-6 production in epithelial cells
Source: Front Cell Infect Microbiol. 2025 Nov 11;15:1688013. doi: 10.3389/fcimb.2025.1688013 (PMC12643979; doi:10.3389/fcimb.2025.1688013)

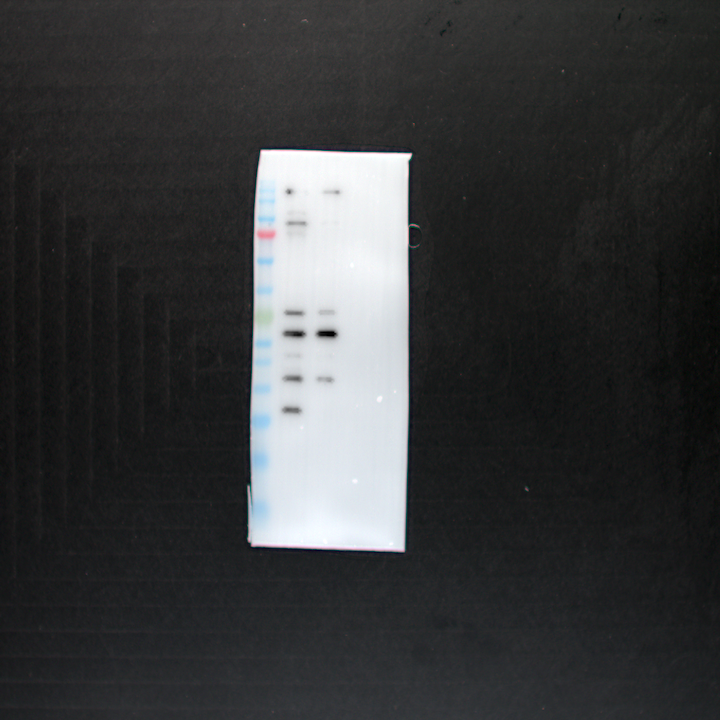

Supplement: Supplementary file 4 [file DataSheet4.zip › Figure S1/FigureS1B_Rab29_WB_raw_maker.Tif]

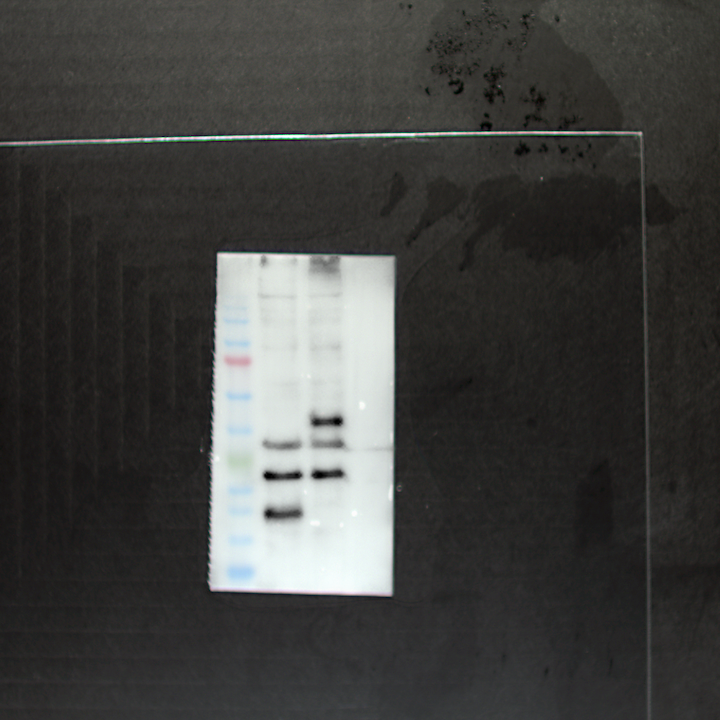

Supplement: Supplementary file 4 [file DataSheet4.zip › Figure S1/FigureS1C_GFP-Rab29_GAPDH_WB_raw_maker.Tif]

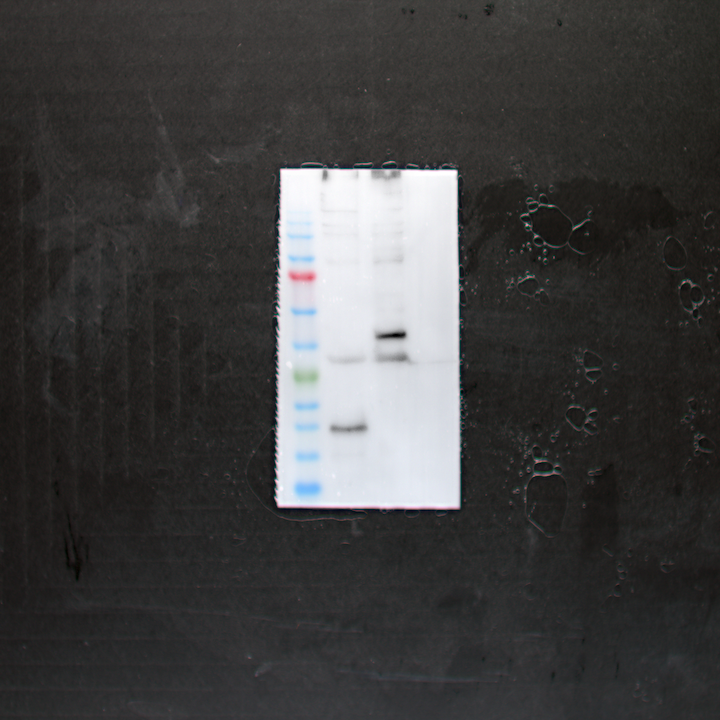

Supplement: Supplementary file 4 [file DataSheet4.zip › Figure S1/FigureS1C_GFP-Rab29_WB_raw_maker.Tif]

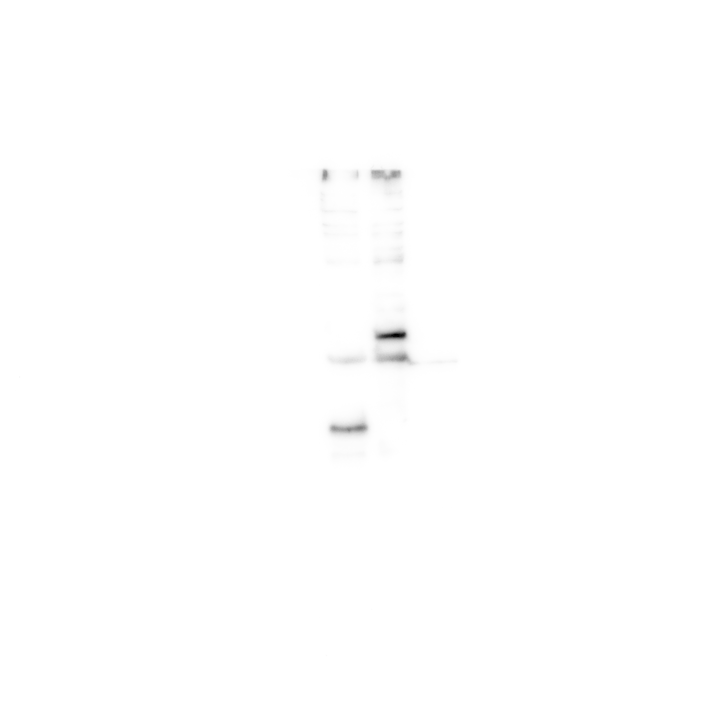

Supplement: Supplementary file 4 [file DataSheet4.zip › Figure S1/FigureS1C_GFPRab29_WB_raw_exposure.Tif]

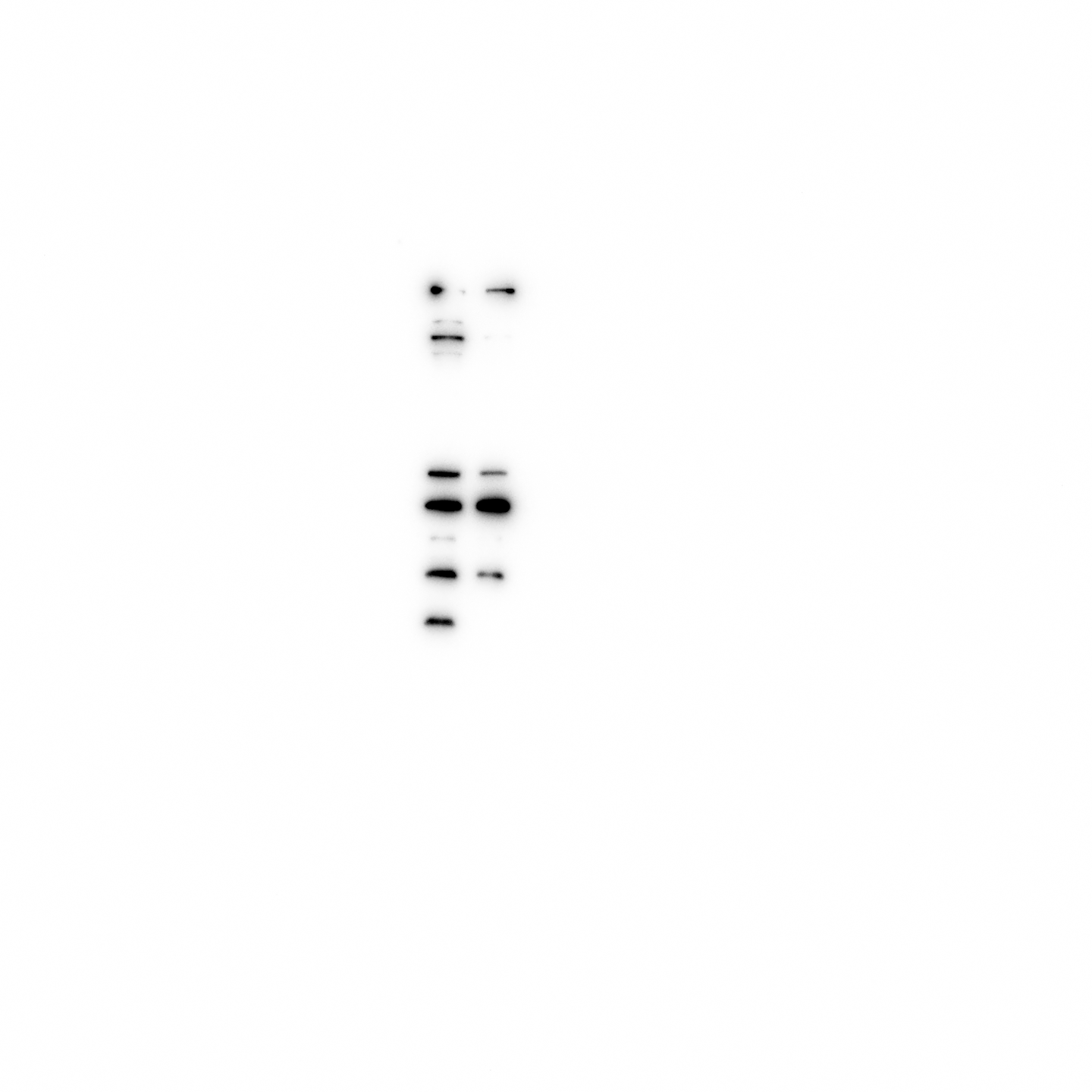

Supplement: Supplementary file 4 [file DataSheet4.zip › Figure S1/FigureS1B_Rab29_WB_raw_exposure.Tif]

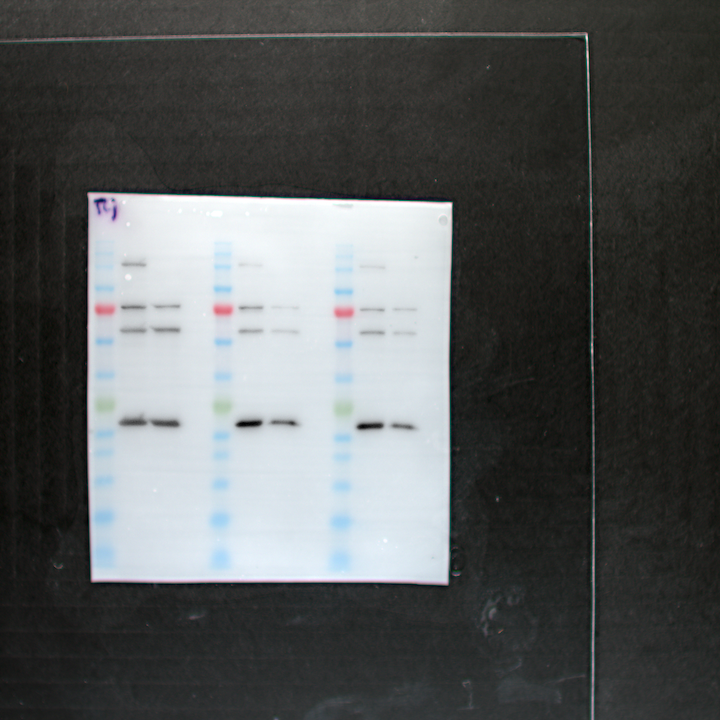

Supplement: Supplementary file 4 [file DataSheet4.zip › Figure S1/FigureS1A_TBC1D9_WB_raw_maker.Tif]

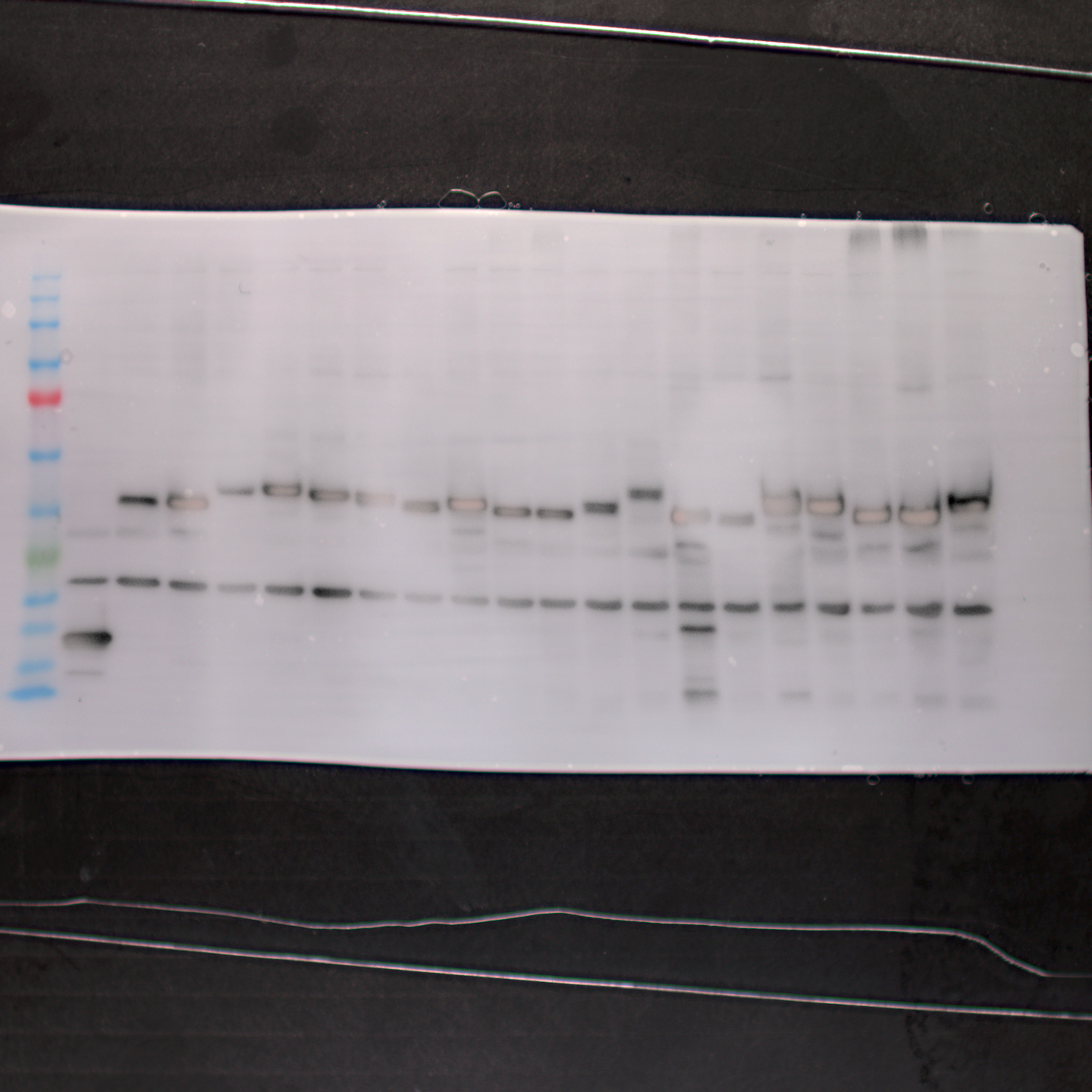

Supplement: Supplementary file 5 [file DataSheet5.zip › Figure S4A/FigureS4A_GFP-Rabs_antigapdh_WB_raw_maker.Tif]

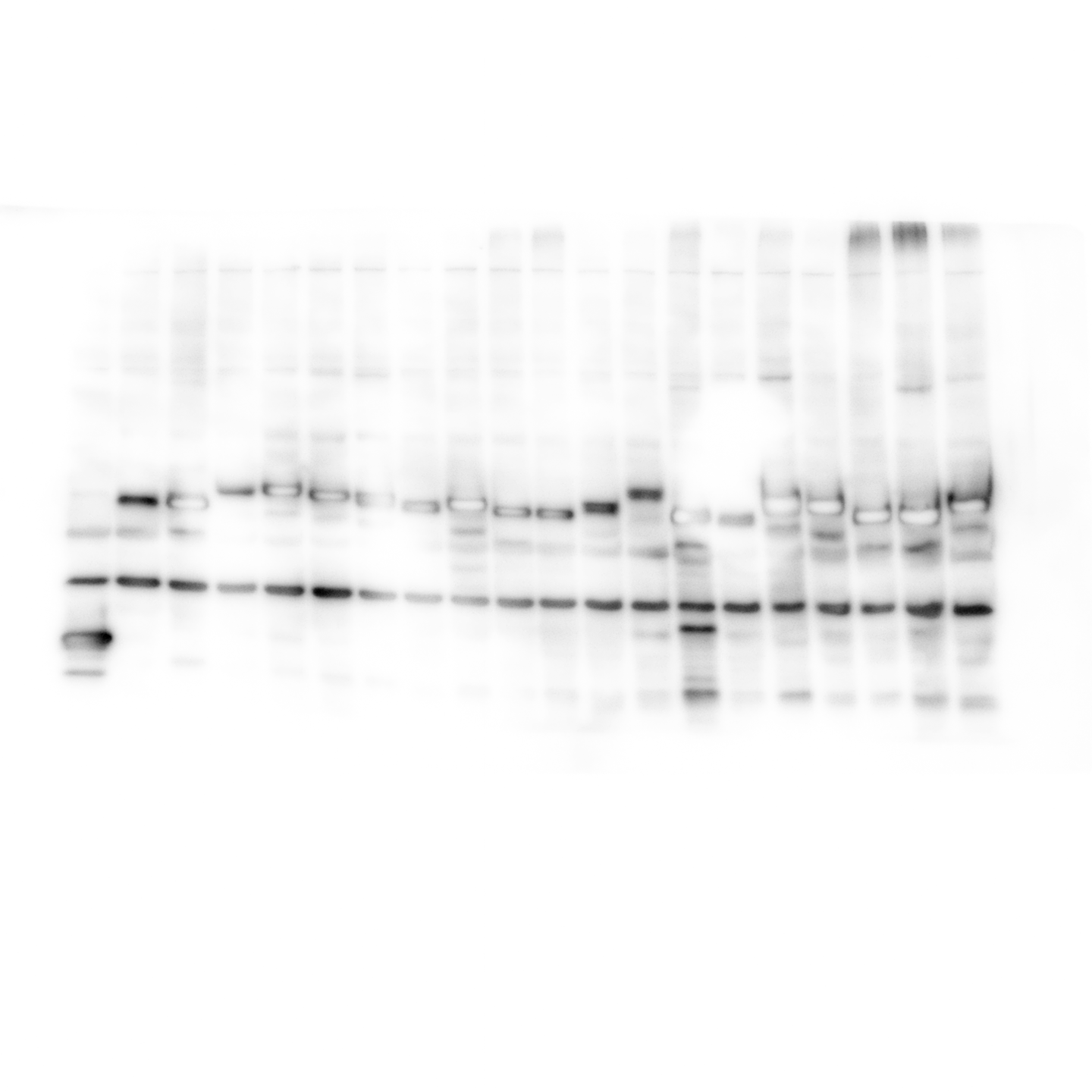

Supplement: Supplementary file 5 [file DataSheet5.zip › Figure S4A/FigureS4A_GFP-Rabs_antigapdh_WB_raw_exposure.Tif]

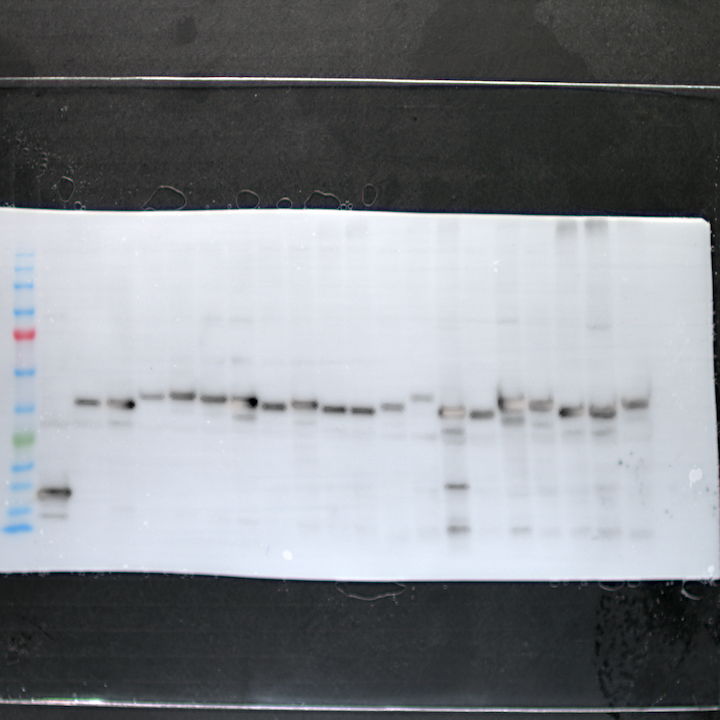

Supplement: Supplementary file 5 [file DataSheet5.zip › Figure S4A/FigureS4A_GFP-Rabs_WB_raw_maker.Tif]

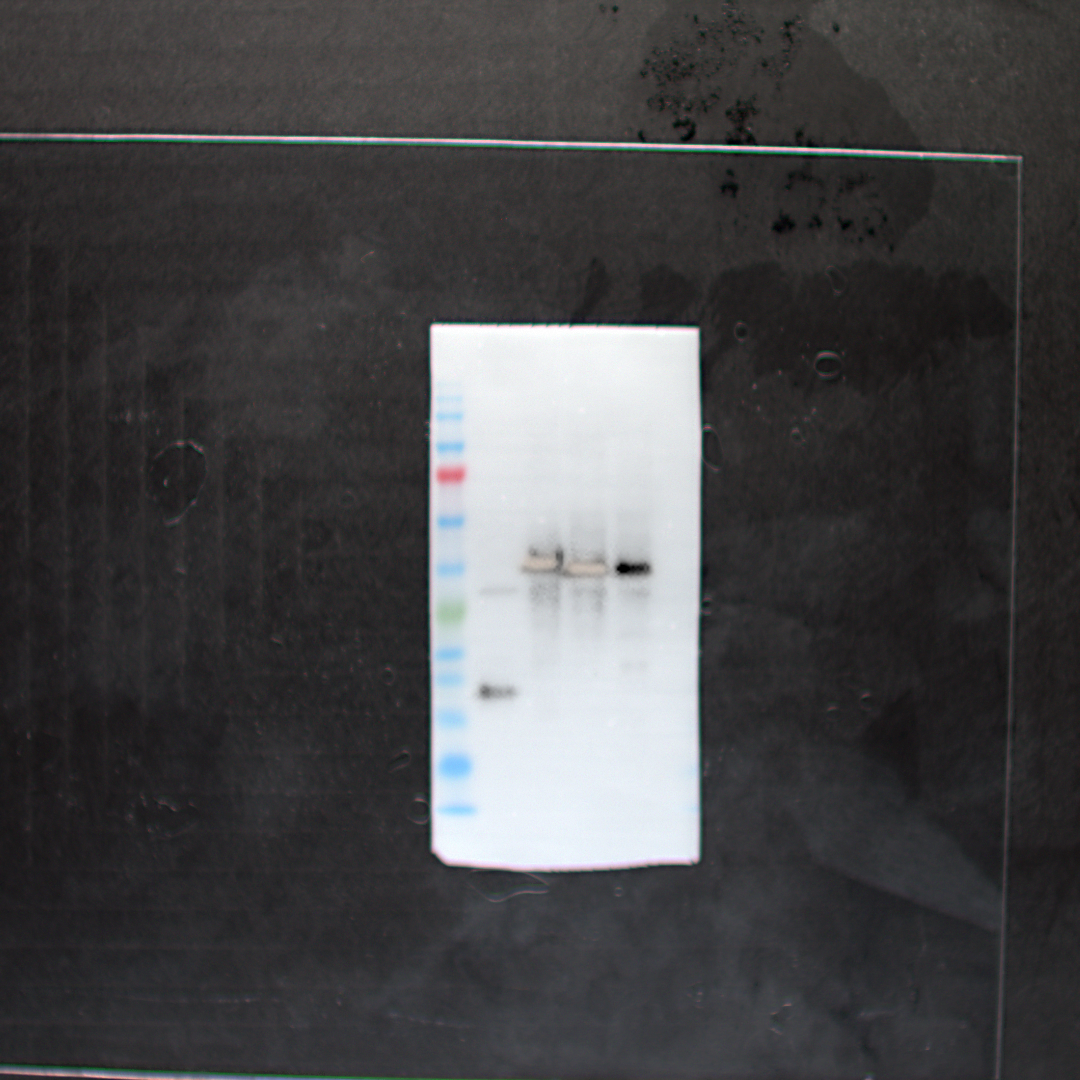

Supplement: Supplementary file 6 [file DataSheet6.zip › Figure S6A/FigureS6A_GFP_WB_raw_maker.Tif]

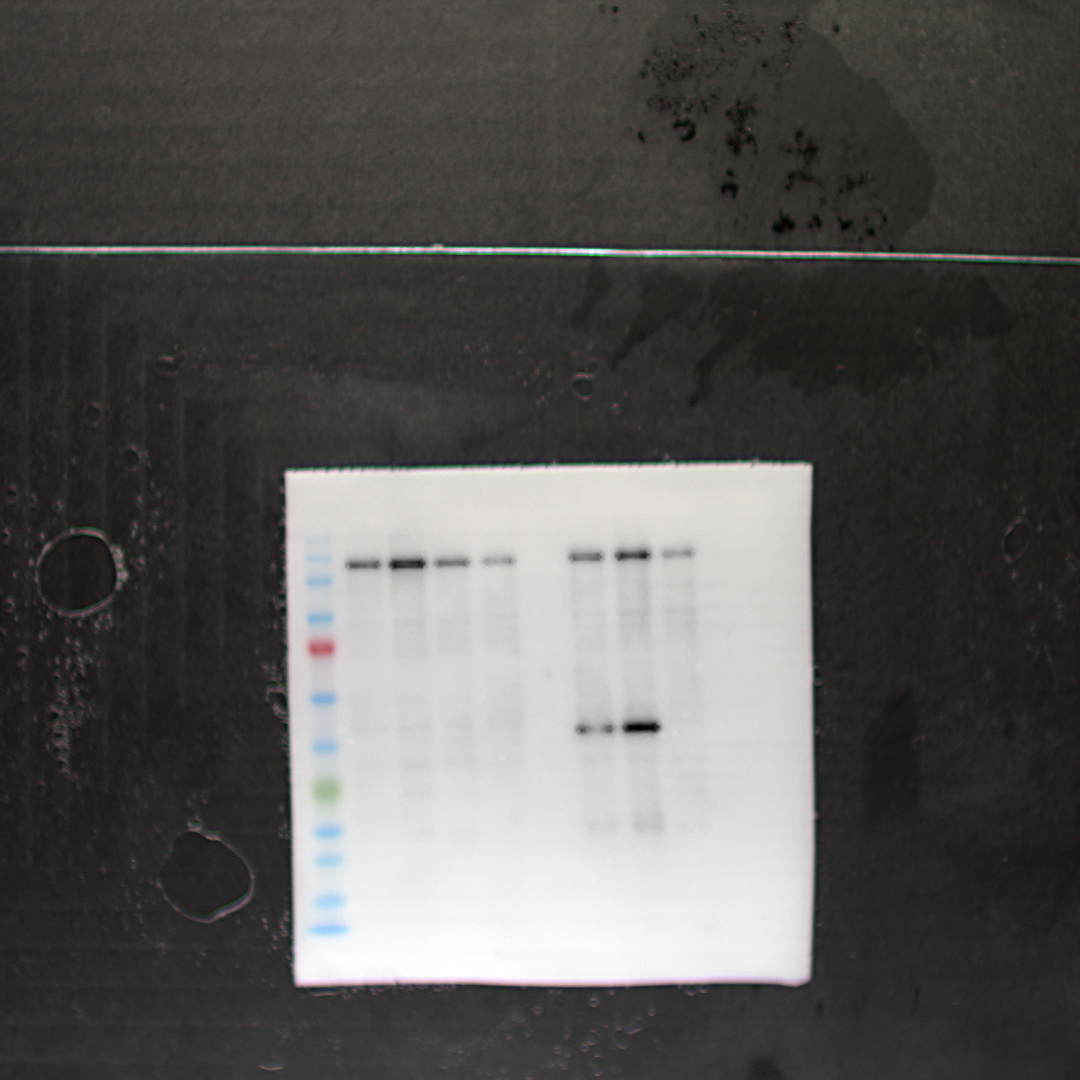

Supplement: Supplementary file 6 [file DataSheet6.zip › Figure S6A/FigureS6A_Flag_WB_raw_maker.Tif]

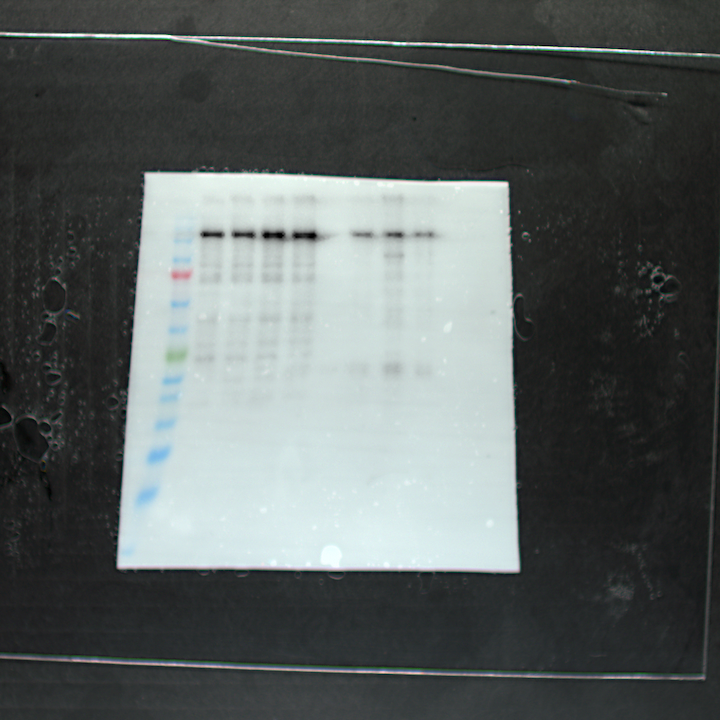

Supplement: Supplementary file 7 [file DataSheet7.zip › Figure S6B/FigureS6B_Flag_WB_raw_maker.Tif]

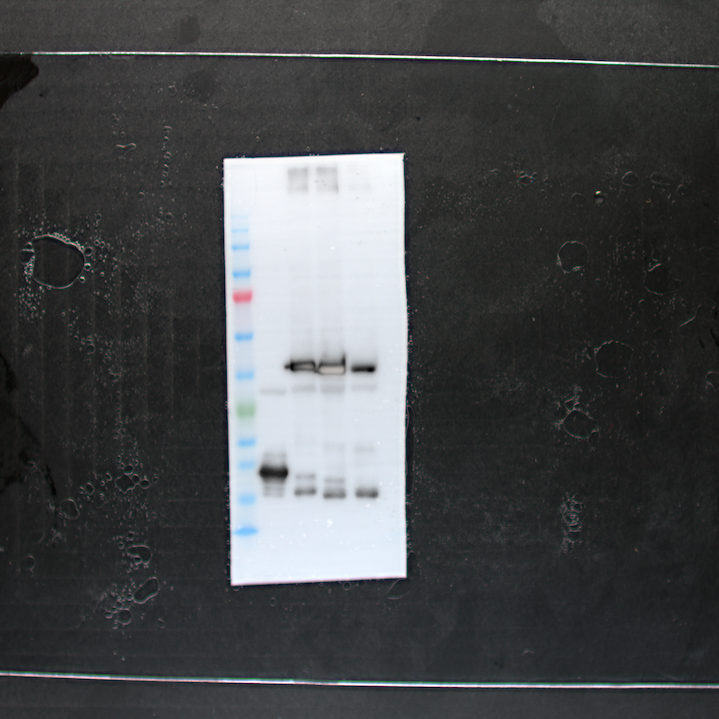

Supplement: Supplementary file 7 [file DataSheet7.zip › Figure S6B/FigureS6B_GFP_WB_raw_maker.Tif]

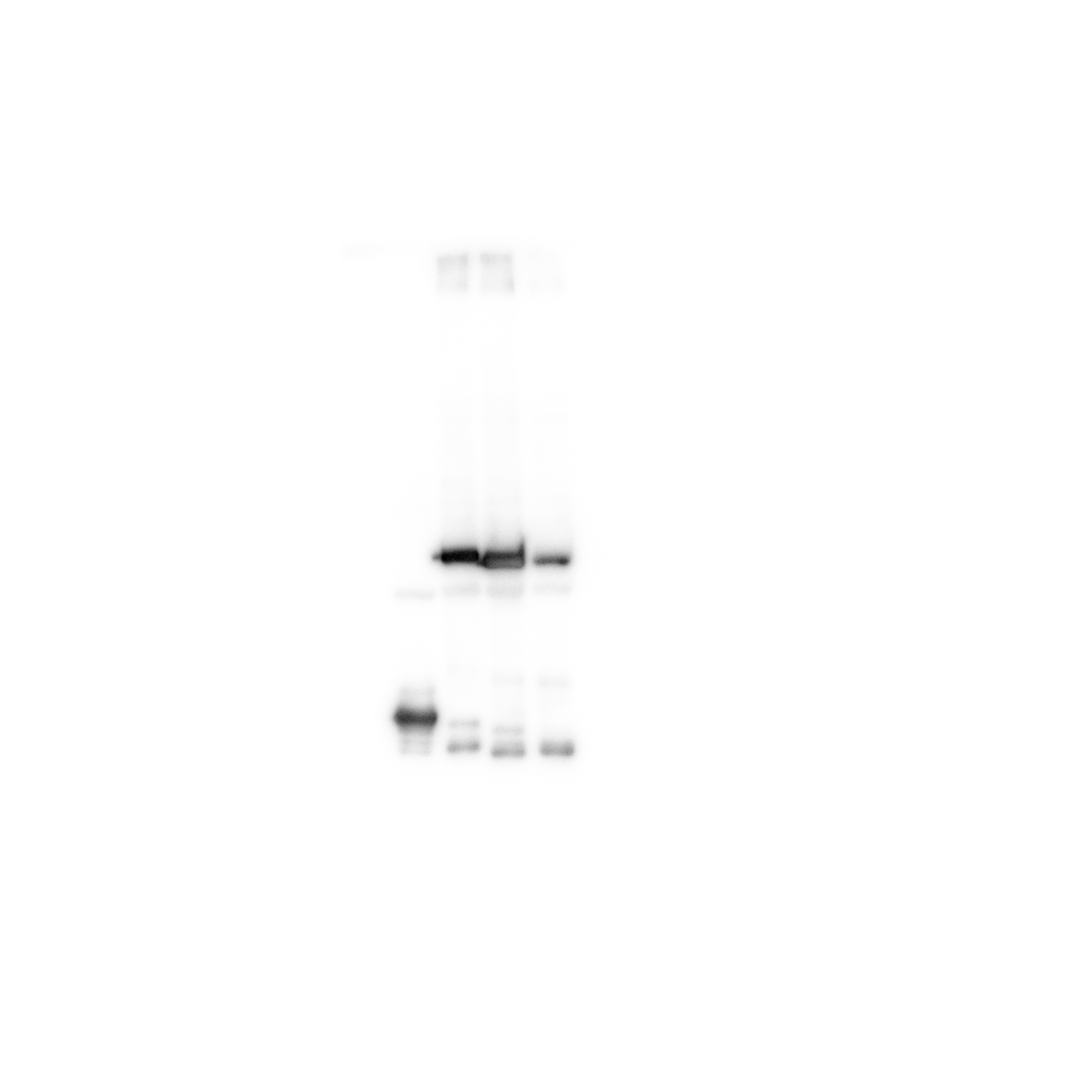

Supplement: Supplementary file 7 [file DataSheet7.zip › Figure S6B/FigureS6B_GFP_WB_raw_exposure.Tif]
