# Supplementary material for: A TBC1D9-Rab29 axis controls homeostatic NF-κB signaling and selective IL-6 production in epithelial cells
Source: Front Cell Infect Microbiol. 2025 Nov 11;15:1688013. doi: 10.3389/fcimb.2025.1688013 (PMC12643979; doi:10.3389/fcimb.2025.1688013)
Supplement: Supplementary file 8 [file DataSheet8.pdf]

Supplementary Figure S9

Figure 2B (main text)

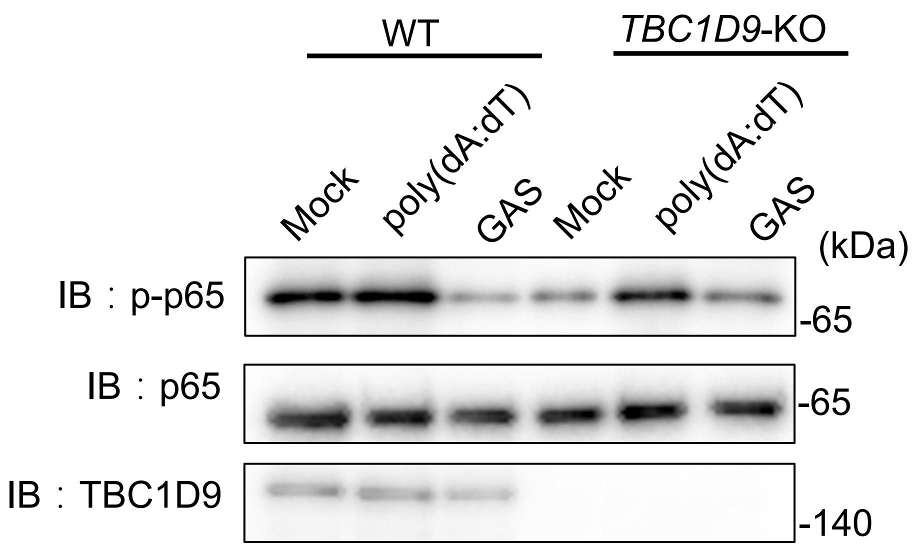

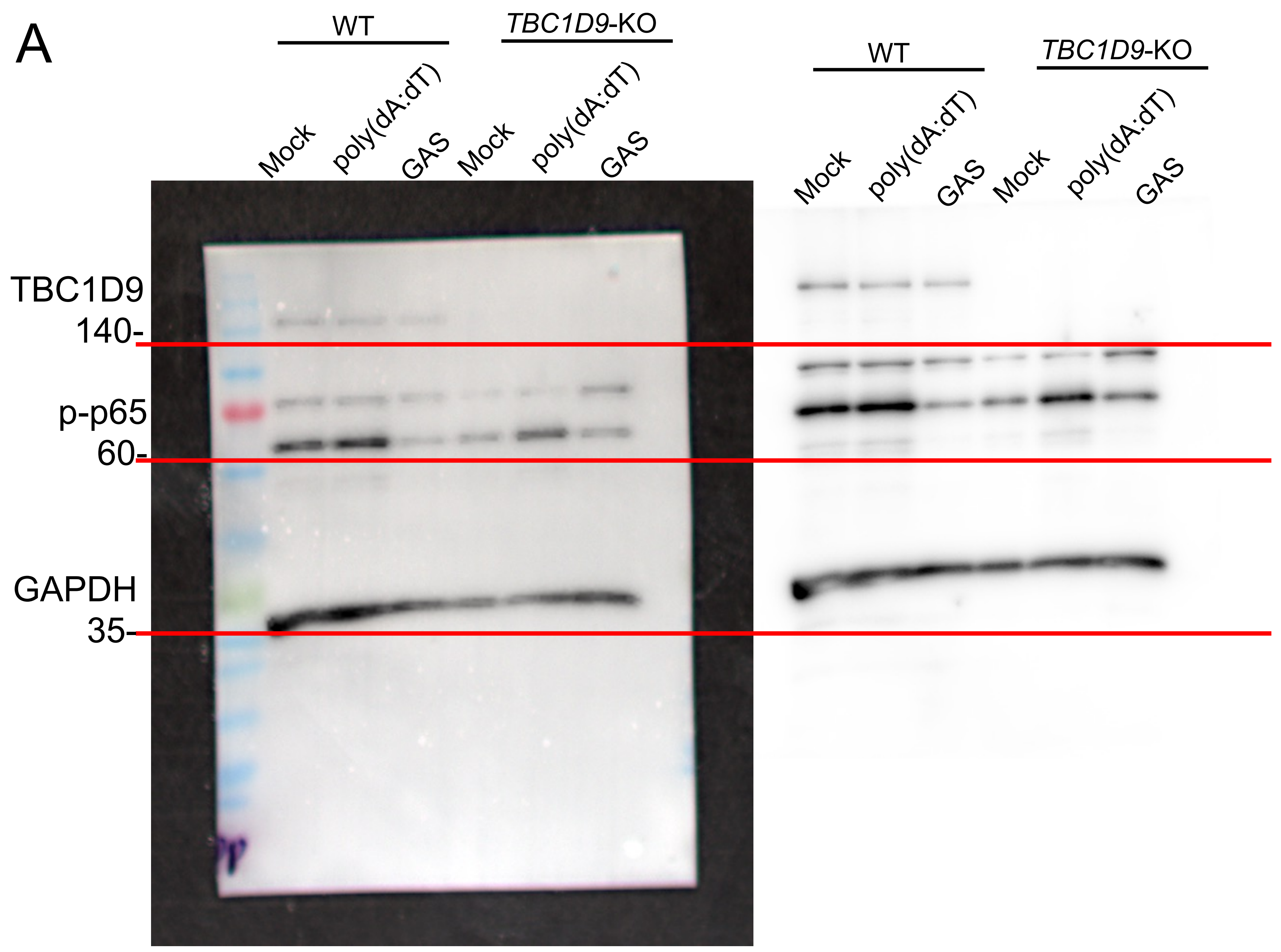

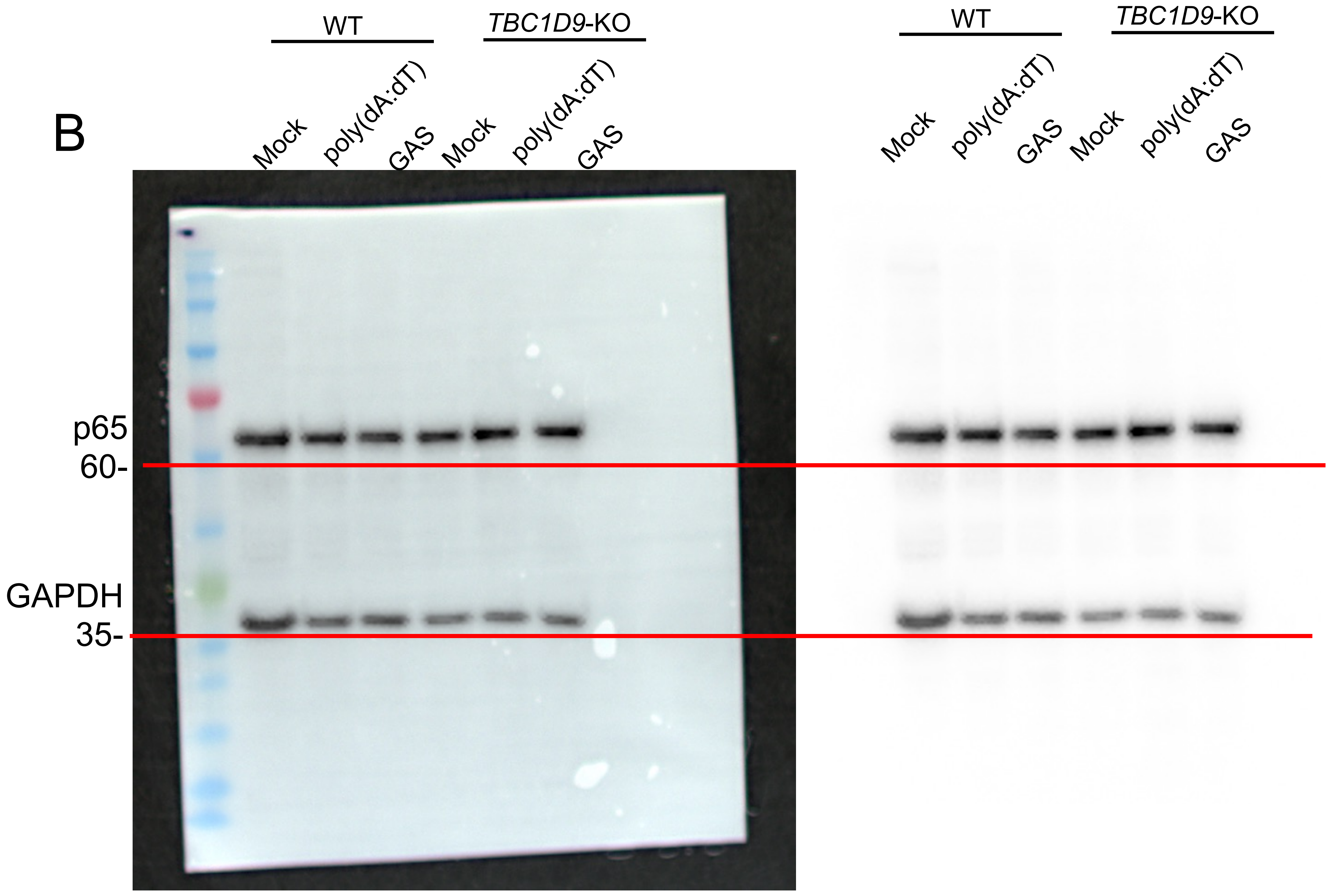

Supplementary Figure S4. Full-length blots for Figure 2B (main text).

Uncropped original Western blot images corresponding to Figure 2B. For each blot, the left panel shows the colorimetric scan with molecular weight marker, and the right panel shows the chemiluminescent exposure used for quantification.

Panel A: TBC1D9, p-p65, and GAPDH blot, original membrane.

Panel B: p65 and GAPDH blot, original membrane.

Supplementary Figure S10

Figure 3D (main text)

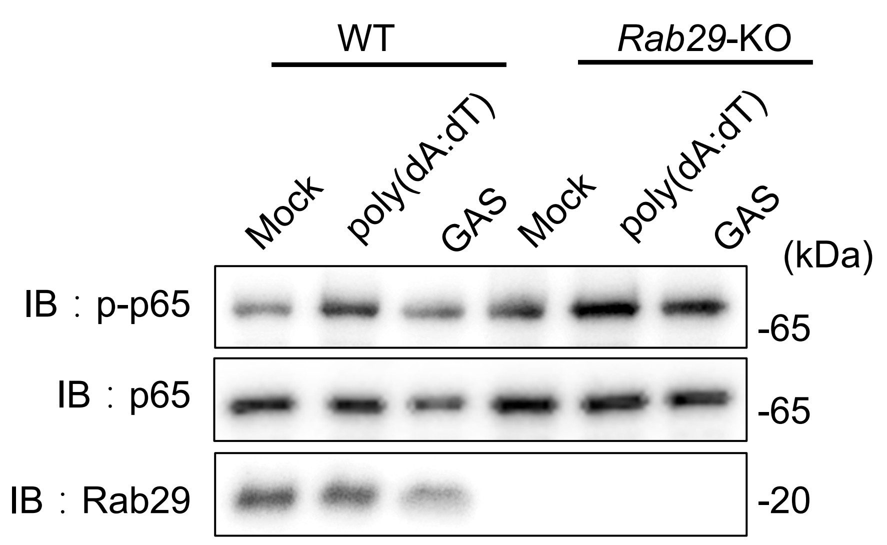

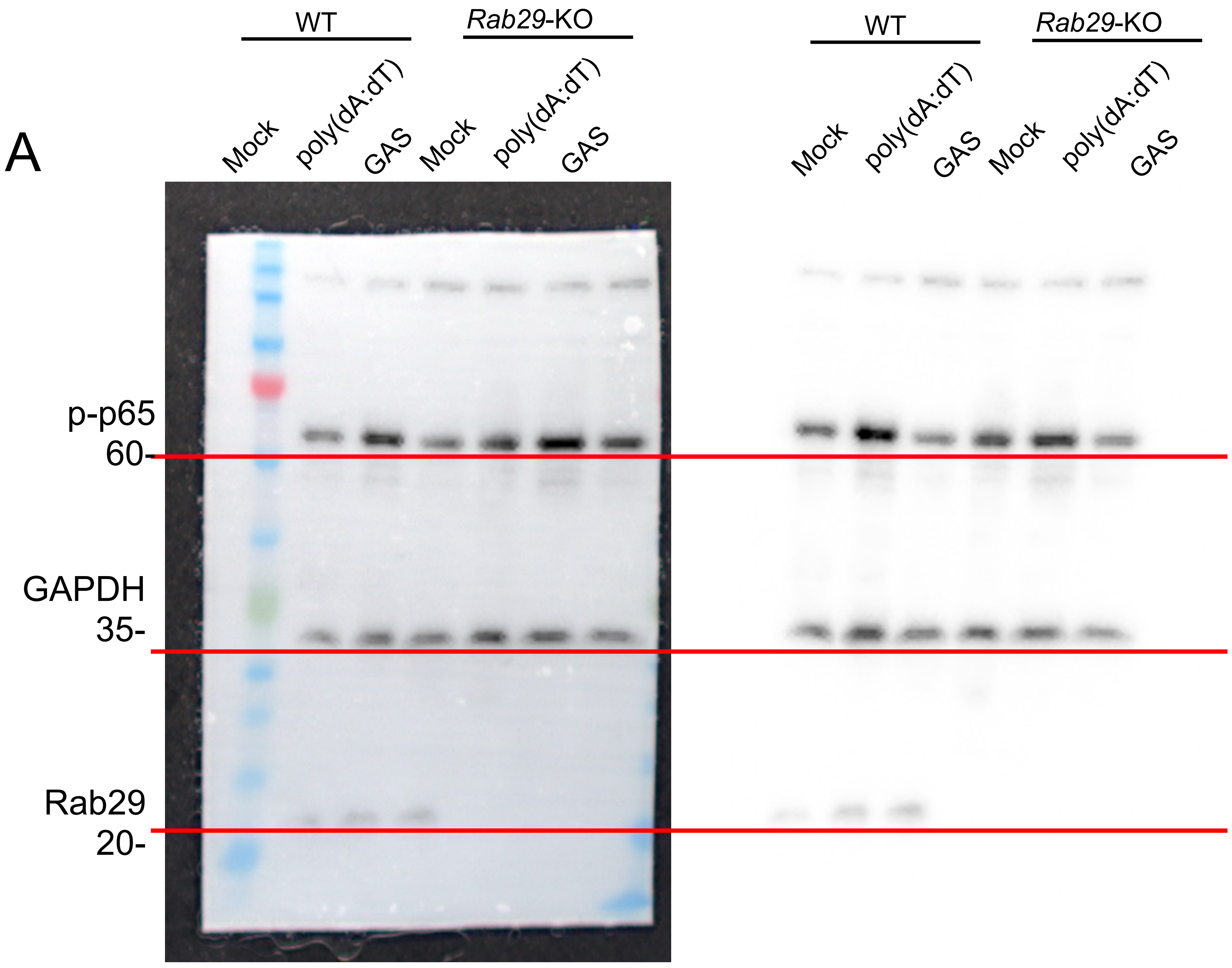

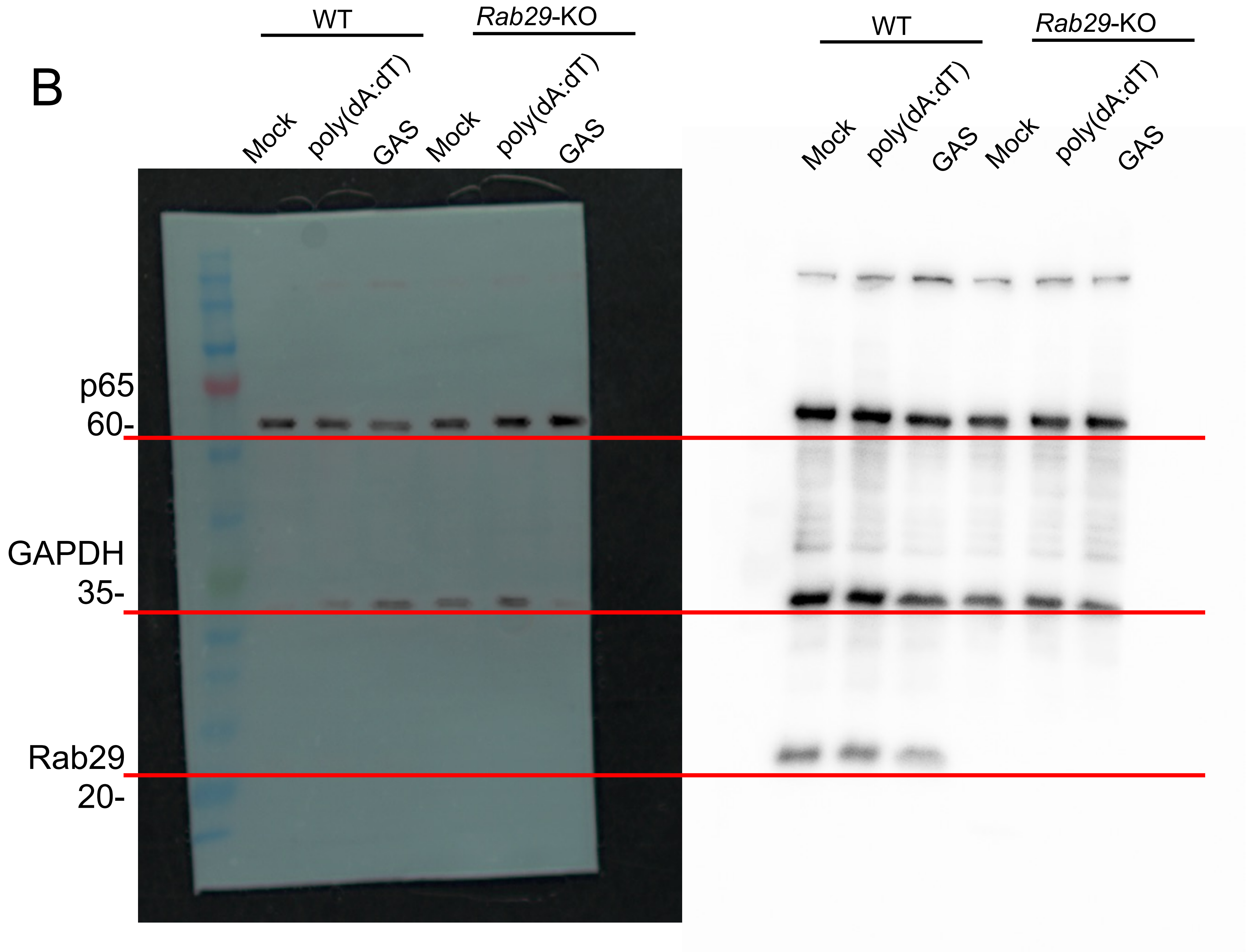

Supplementary Figure S5. Full-length blots for Figure 3D (main text).

Uncropped original Western blot images corresponding to Figure 3D. For each blot, the left panel shows the colorimetric scan with molecular weight marker, and the right panel shows the chemiluminescent exposure used for quantification.

Panel A: p-p65, GAPDH, and Rab29 blot, original membrane.

Panel B: p65, GAPDH, and Rab29 blot, original membrane.

Supplementary Figure S11

Figure 5C (main text)

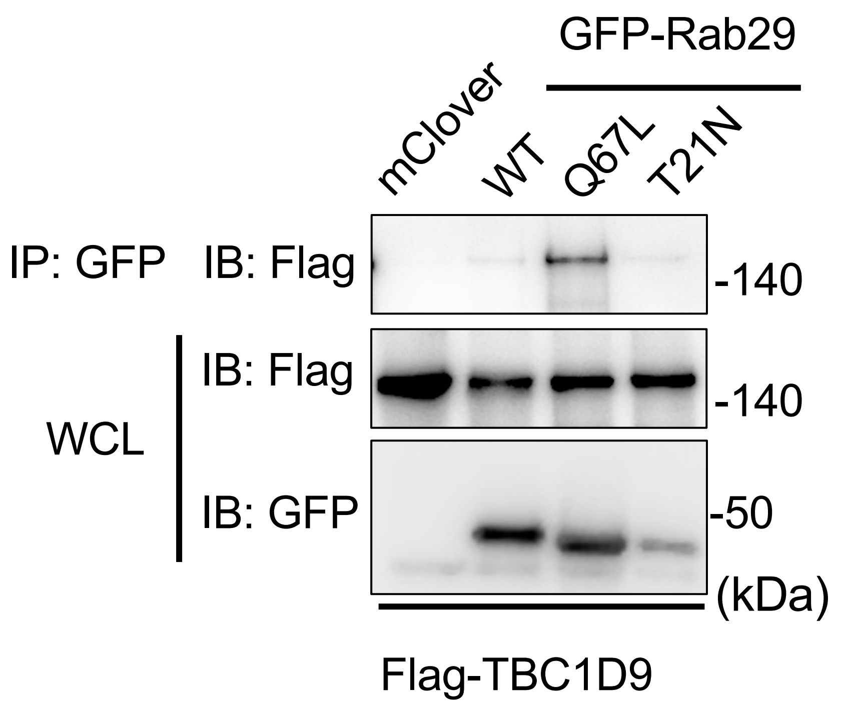

A

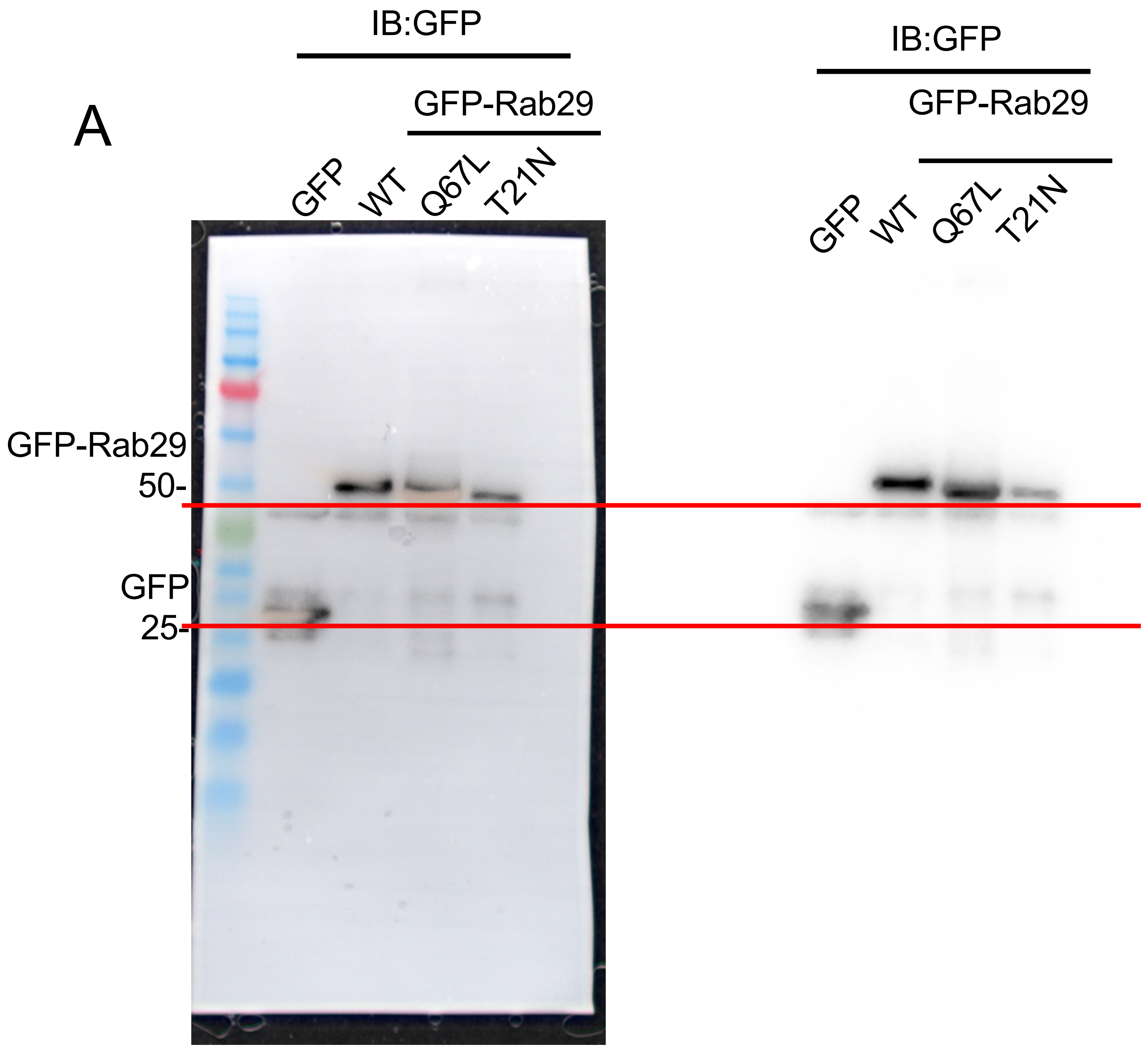

B

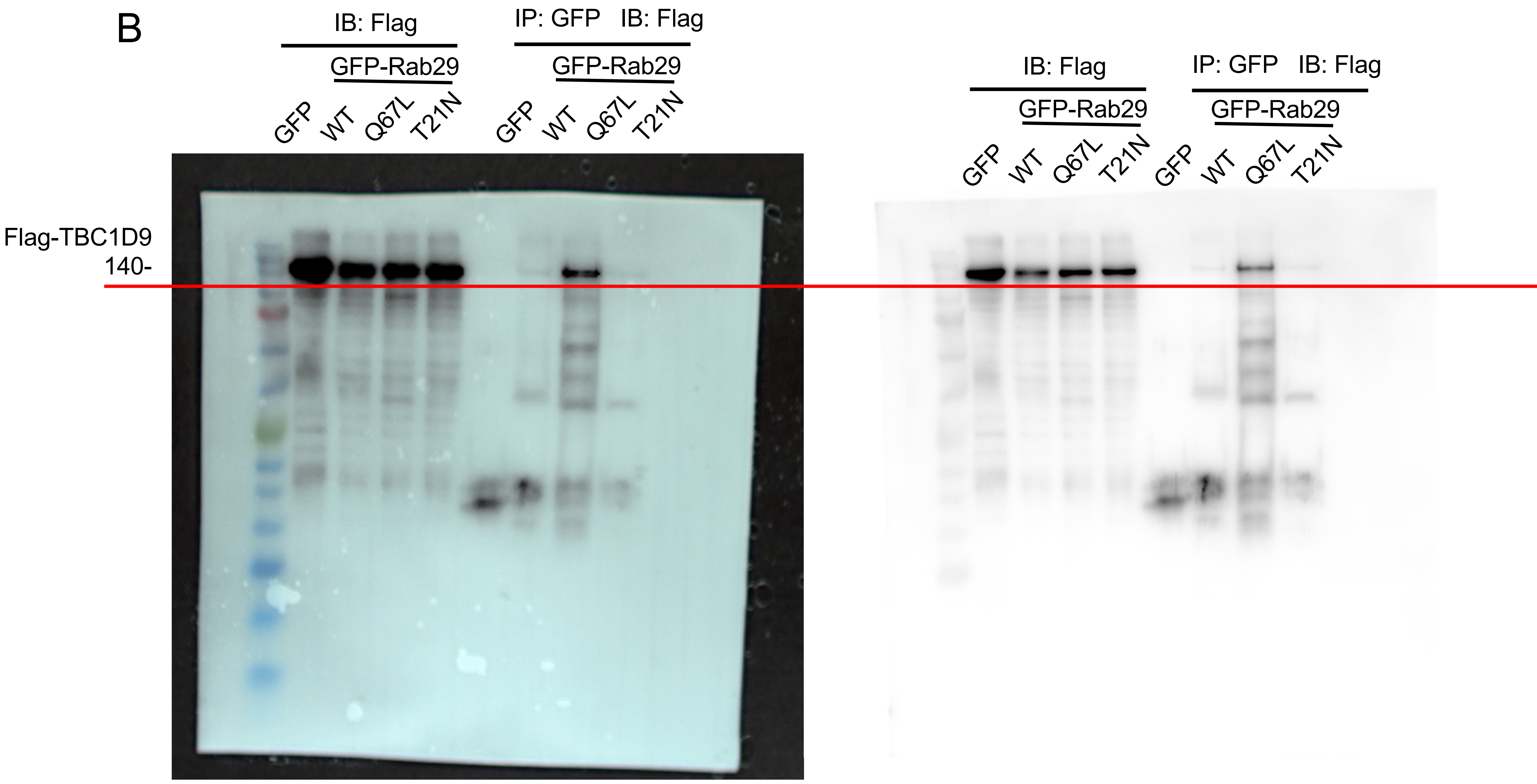

Supplementary Figure S6. Full-length blots for Figure 5C (main text).

Uncropped original Western blot images corresponding to Figure 5C. For each blot, the left panel shows the colorimetric scan with molecular weight marker, and the right panel shows the chemiluminescent exposure used for quantification.

Panel A: GFP and GFP-Rab29 blot, original membrane.

Panel B: Flag-TBC1D9 blot, original membrane.

Supplementary Figure S12

Figure S1A (main text)

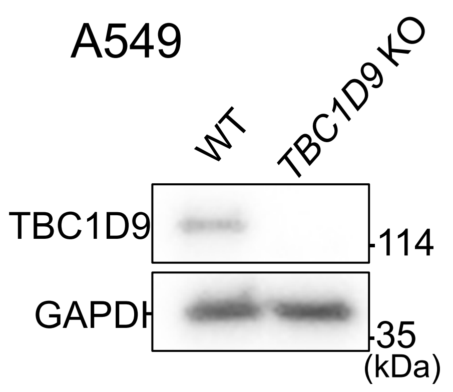

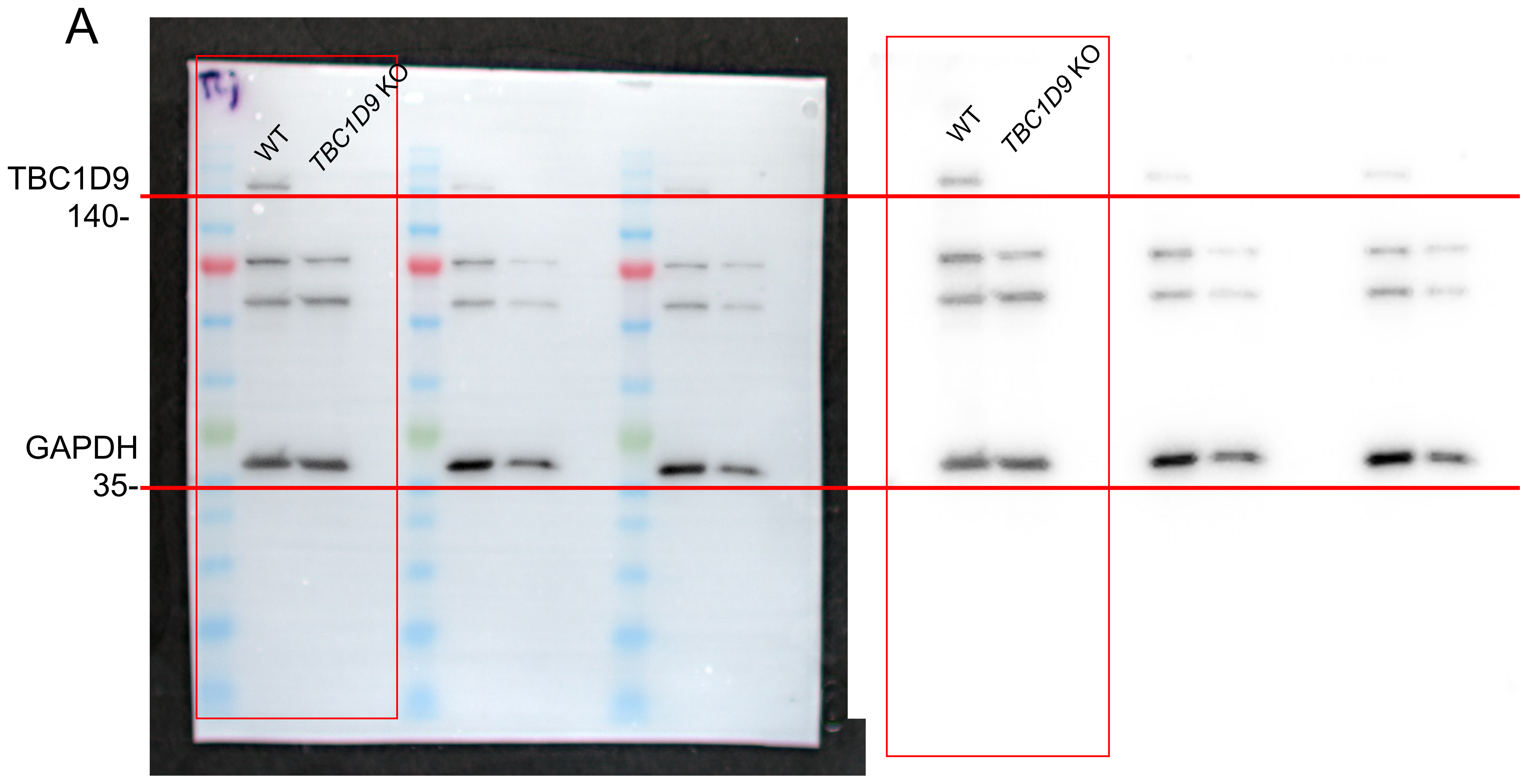

Supplementary Figure S7. Full-length blots for Figure S1A (main text).

Uncropped original Western blot images corresponding to Figure S1A. The left panel shows the colorimetric scan with molecular weight marker, and the right panel shows the chemiluminescent exposure used for quantification.

Panel A: TBC1D9 and GAPDH blot, original membrane.

Figure S1B (main text)

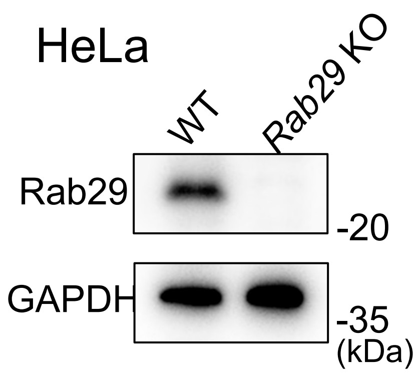

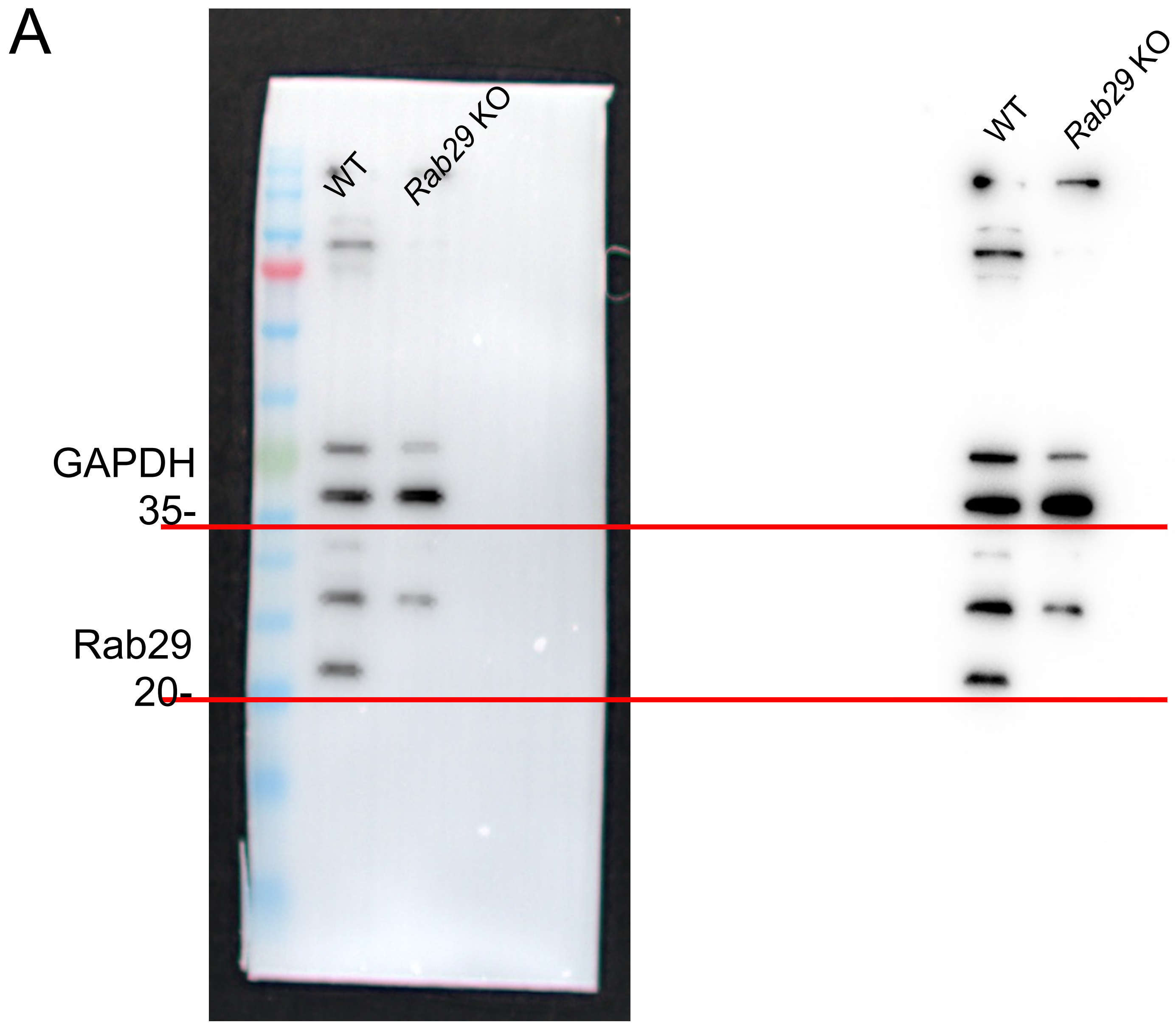

Supplementary Figure S8. Full-length blots for Figure S1B (main text).

Uncropped original Western blot images corresponding to Figure S1B. The left panel shows the colorimetric scan with molecular weight marker, and the right panel shows the chemiluminescent exposure used for quantification.

Panel A: Rab29 and GAPDH blot, original membrane.

Supplementary Figure S12

Figure S1C (main text)

C

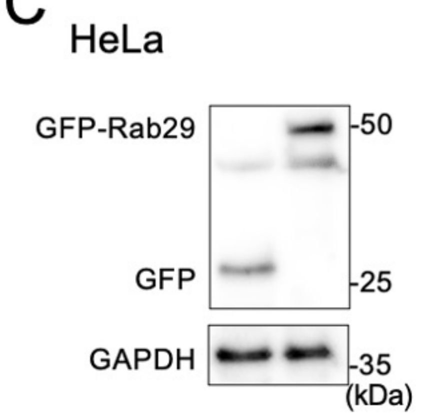

A

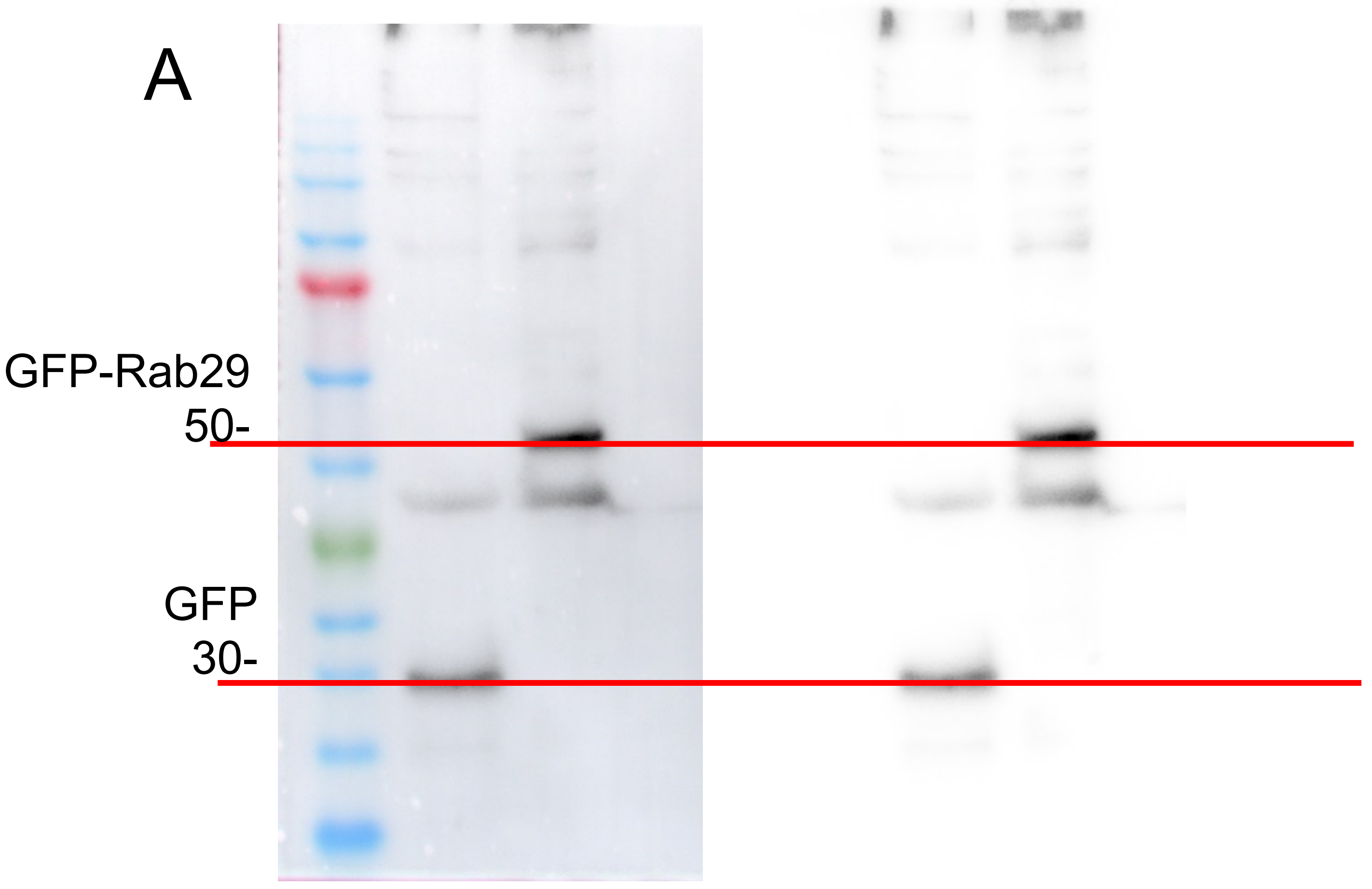

B

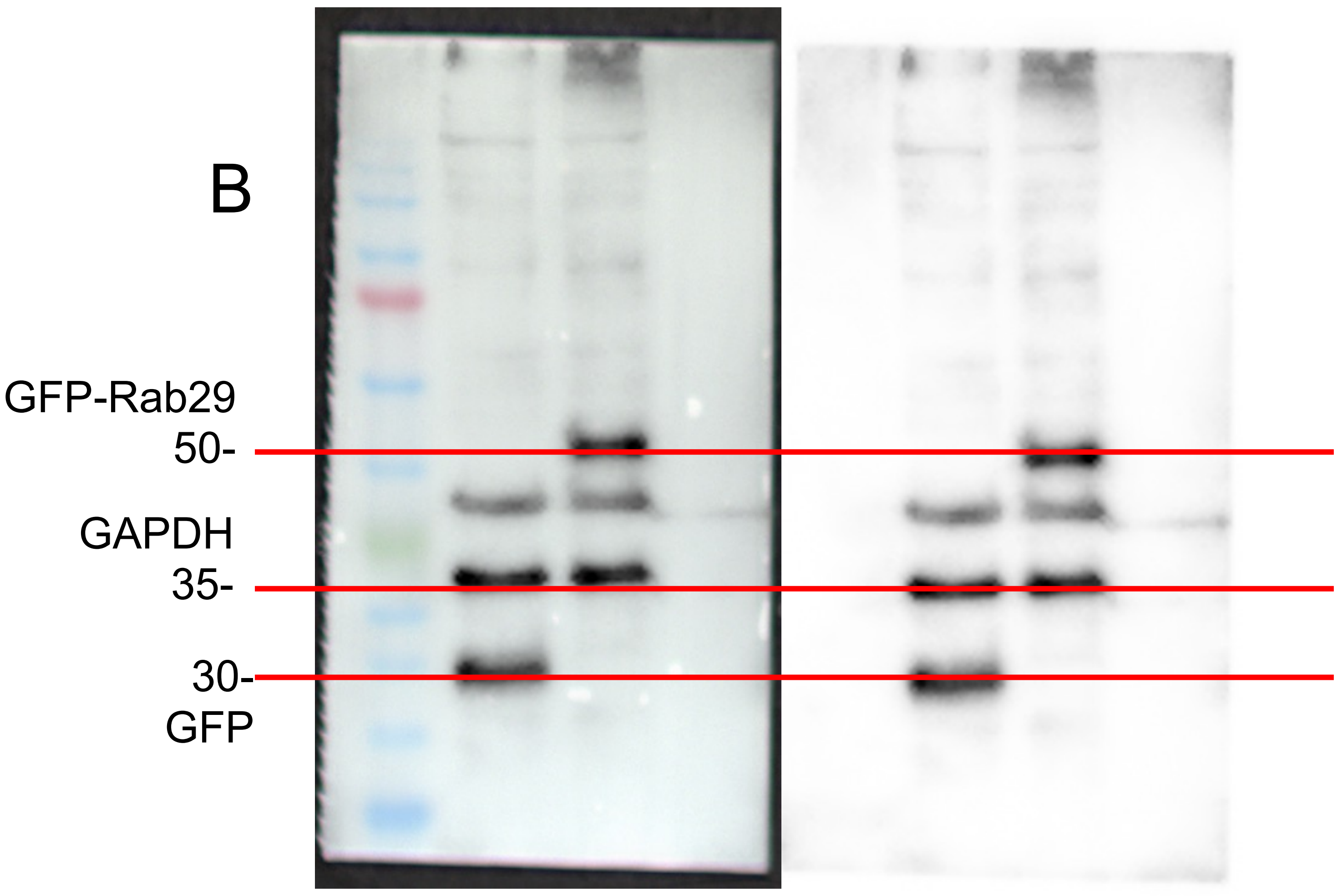

Supplementary Figure S12. Full-length blots for Figure S1A (main text).

Uncropped original Western blot images corresponding to Figure S1C. The left panel shows the colorimetric scan with molecular weight marker, and the right panel shows the chemiluminescent exposure used for quantification.

Panel A: GFP-Rab29 and GFP blot, original membrane.

Panel B: GAPDH blot, original membrane.

Supplementary Figure S13

Figure S4A (main text)

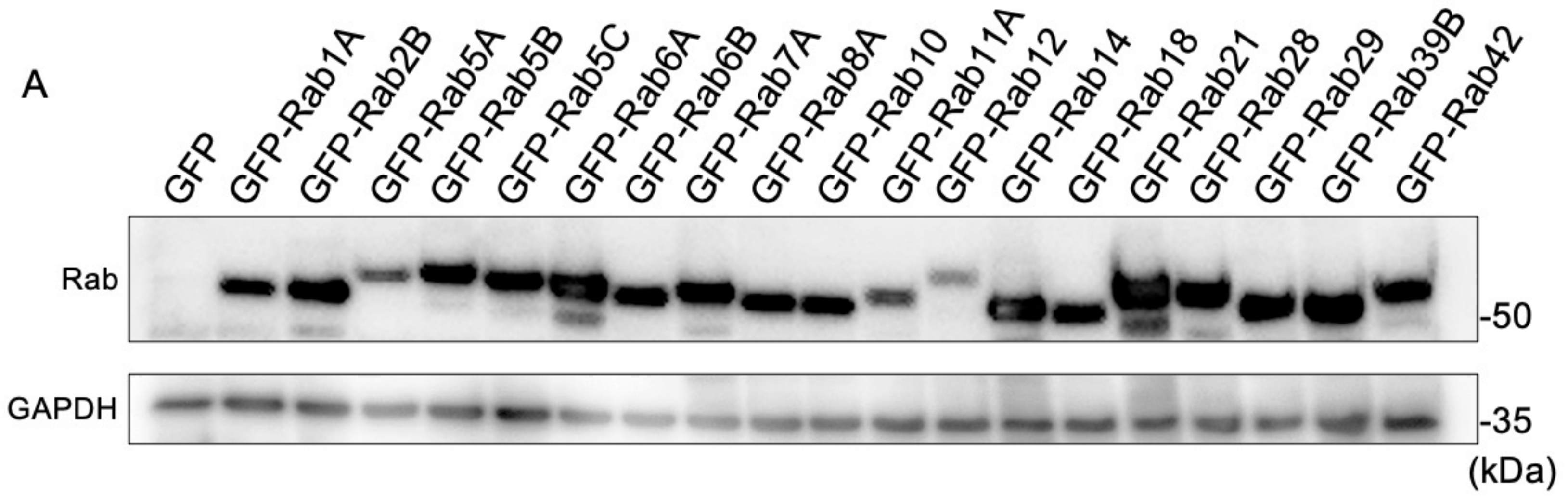

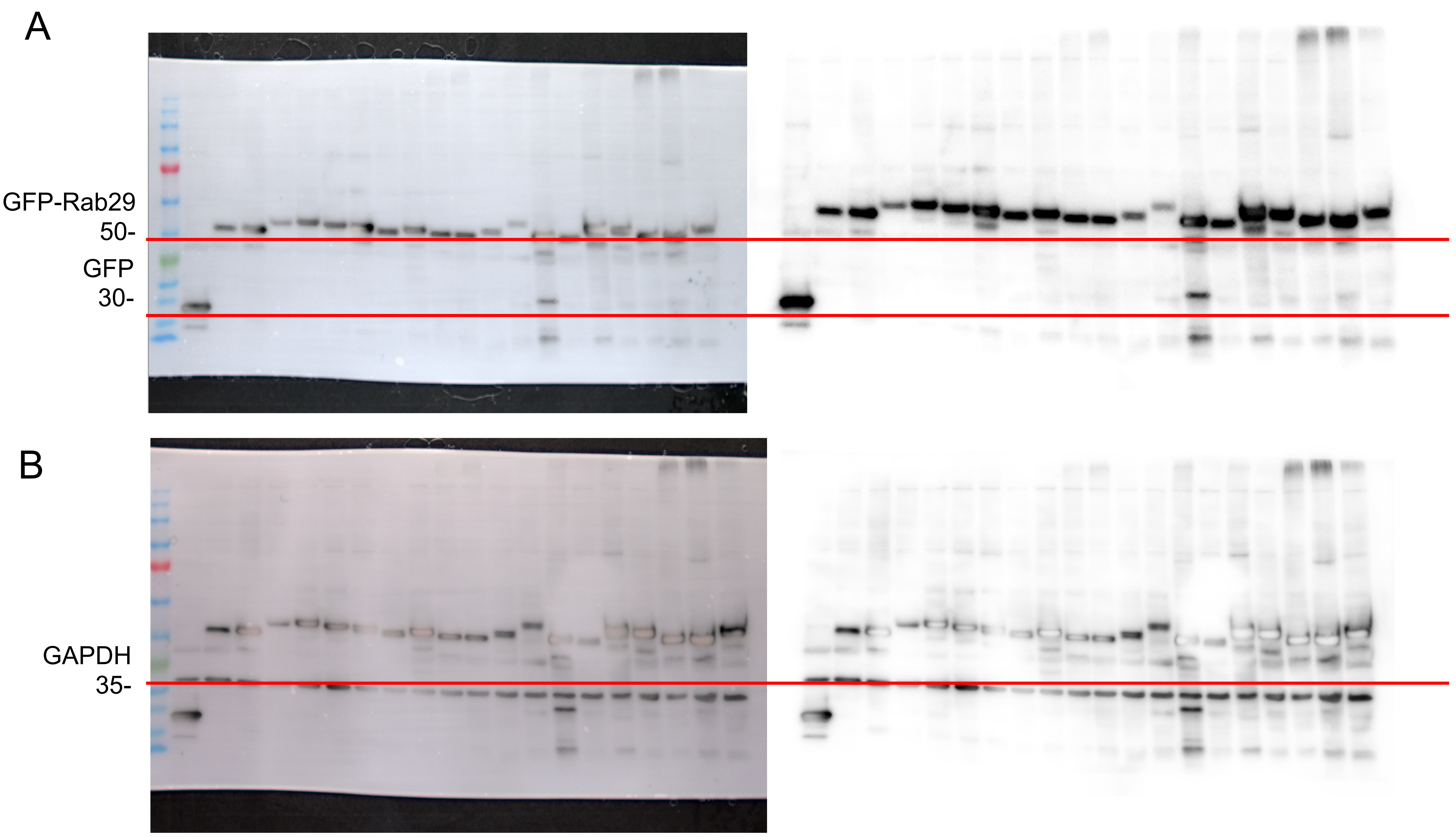

Supplementary Figure S13. Full-length blots for Figure S4A (main text).

Uncropped original Western blot images corresponding to Figure S4A. The left panel shows the colorimetric scan with molecular weight marker, and the right panel shows the chemiluminescent exposure used for quantification.

Panel A: GFP-Rab29 and GFP blot, original membrane.

Panel B: GAPDH blot, original membrane.

Supplementary Figure S14

Figure S6A (main text)

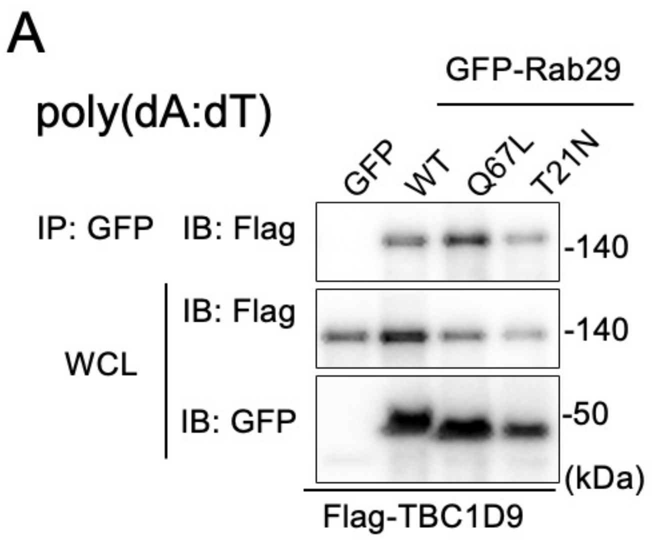

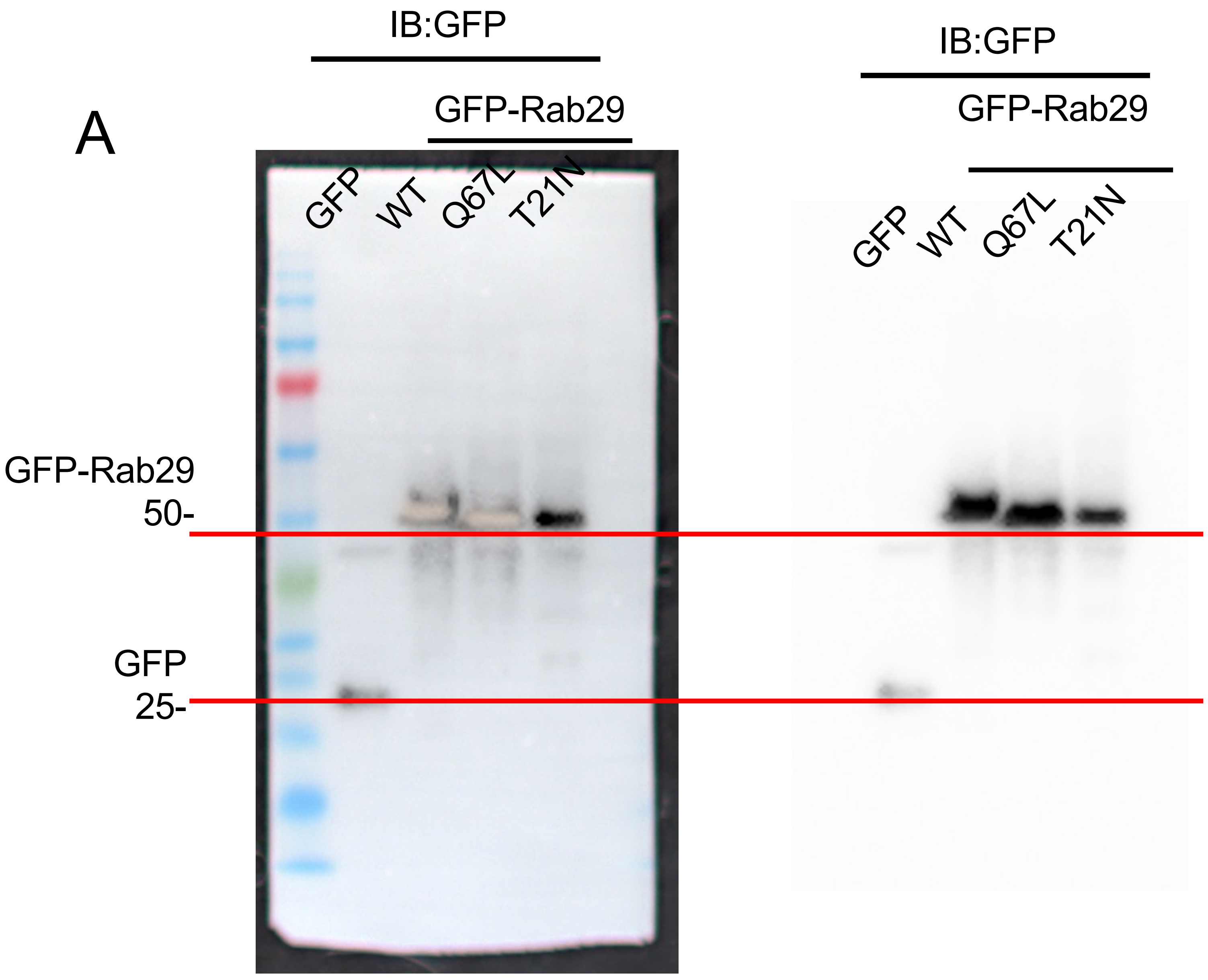

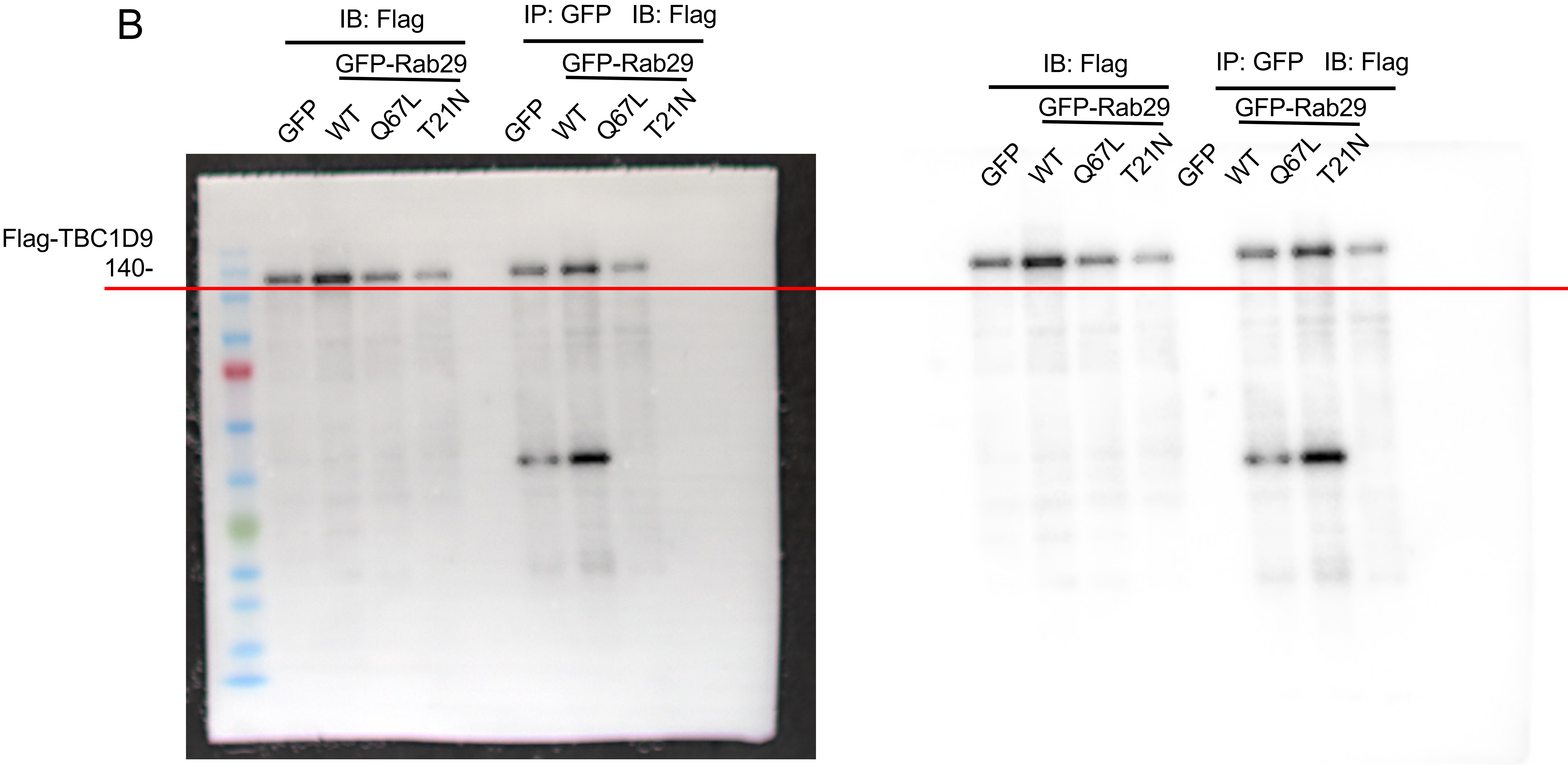

Supplementary Figure S14. Full-length blots for Figure S6A (main text).

Uncropped original Western blot images corresponding to Figure S6A. For each blot, the left panel shows the colorimetric scan with the molecular weight marker, and the right panel shows the chemiluminescent exposure used for quantification.

Panel A: GFP and GFP-Rab29 blot, original membrane.

Panel B: Flag-TBC1D9 blot, original membrane.

Supplementary Figure S15

Figure S6B (main text)

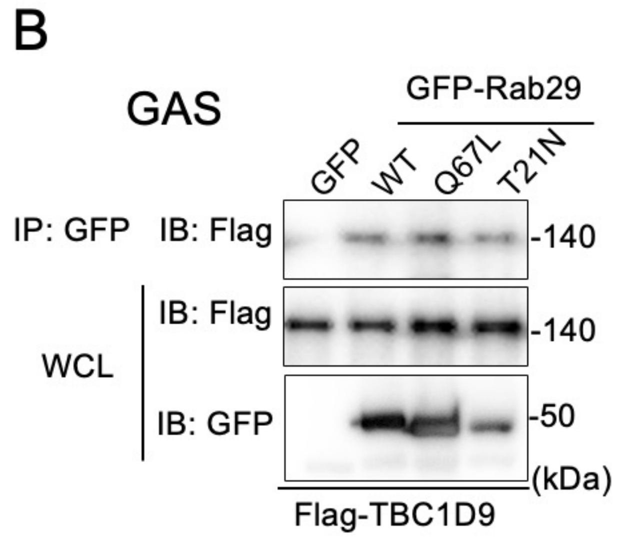

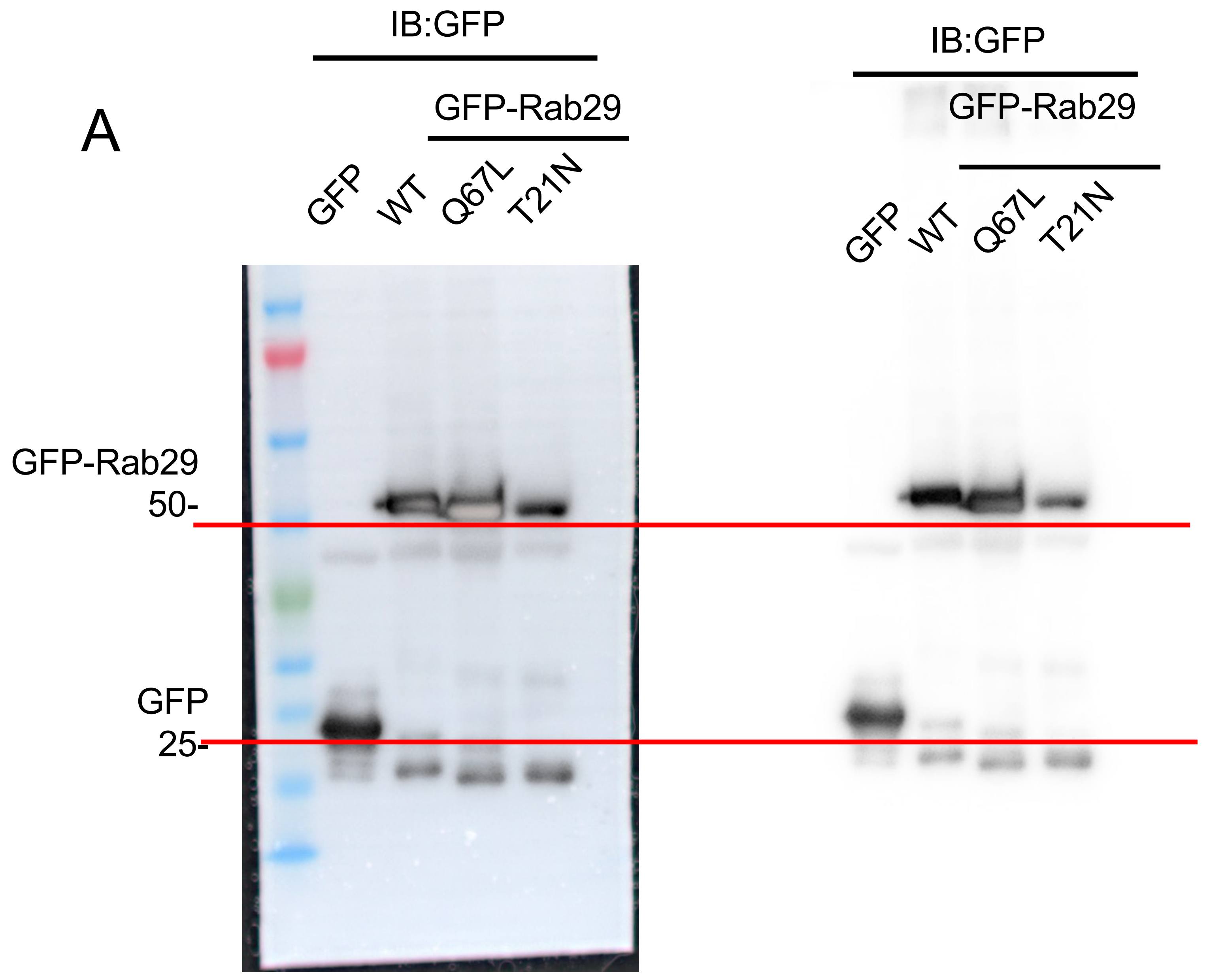

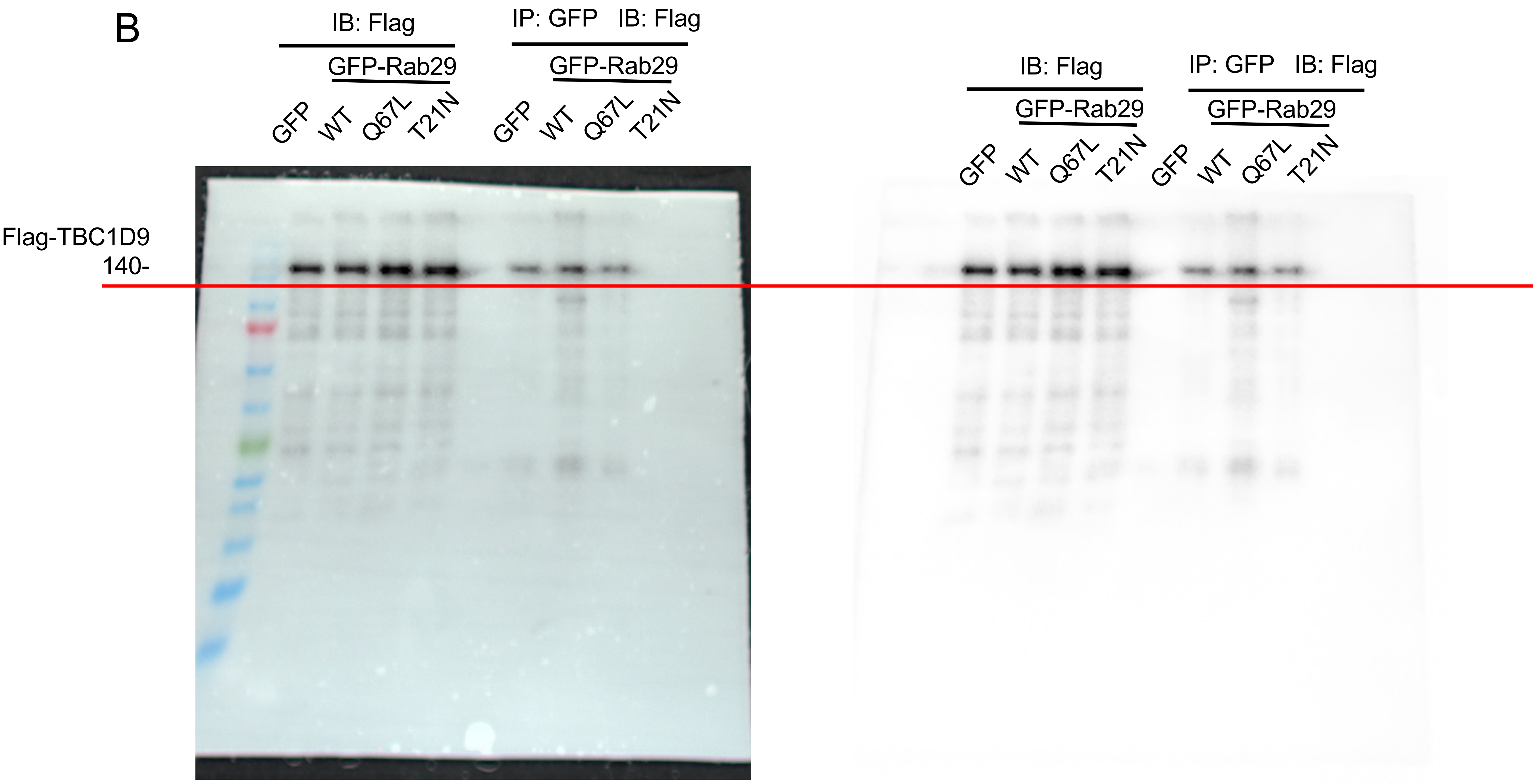

Supplementary Figure S15. Full-length blots for Figure S6B (main text).

Uncropped original Western blot images corresponding to Figure S6B. For each blot, the left panel shows the colorimetric scan with the molecular weight marker, and the right panel shows the chemiluminescent exposure used for quantification.

Panel A: GFP and GFP-Rab29 blot, original membrane.

Panel B: Flag-TBC1D9 blot, original membrane.
